# Supplementary material for: Evaluation of 3′-phosphate as a transient protecting group for controlled enzymatic synthesis of DNA and XNA oligonucleotides
Source: Commun Chem. 2022 Jun 1;5:68. doi: 10.1038/s42004-022-00685-5 (PMC9814670; doi:10.1038/s42004-022-00685-5)
Supplement: Supplementary file 1 — Supplementary Information [file 42004_2022_685_MOESM1_ESM.pdf]

Evaluation of 3'-phosphate as a transient protecting group for controlled enzymatic synthesis of DNA and XNA oligonucleotides

Marie Flamme,[a] Steven Hanlon,[b] Irene Marzuoli,[b] Kurt Püntener,[b] Filippo Sladojevich,[c] and Marcel Hollenstein[a,*]

[a] Institut Pasteur, Université de Paris Cité, Department of Structural Biology and Chemistry, Laboratory for Bioorganic Chemistry of Nucleic Acids, CNRS UMR3523, 28, rue du Docteur Roux, 75724 Paris Cedex 15, France

[b] Pharmaceutical Devision, Synthetic Molecules Technical Development, F. Hoffmann-La Roche Ltd, 4070 Basel, Switzerland

[c] Pharma Research and Early Development, Roche Innovation Center Basel, F. Hoffmann-La Roche Ltd, Grenzacherstrasse 124, 4070, Basel, Switzerland

E-mail: marcel.hollenstein@pasteur.fr

1. Additional gel images, LCMS of nucleotides, and docking experiments

2. LC-MS characterizations of products from PEX reactions

3. Oligonucleotides

4. Synthesis of nucleosides and nucleotides

5. Characterization of nucleosides and nucleotides

6. HDX-MS analysis of Klenow polymerase

1. Additional gel images, LCMS of nucleotides, and docking experiments

Template: 3'-G TAC CCG CCG TAC CCT GAC TCG AGT ACG ATC-5'
Primer: 5'-C ATG GGC GGC ATG GGA CTG AGC TCA TGC TAG-3'
15 bps

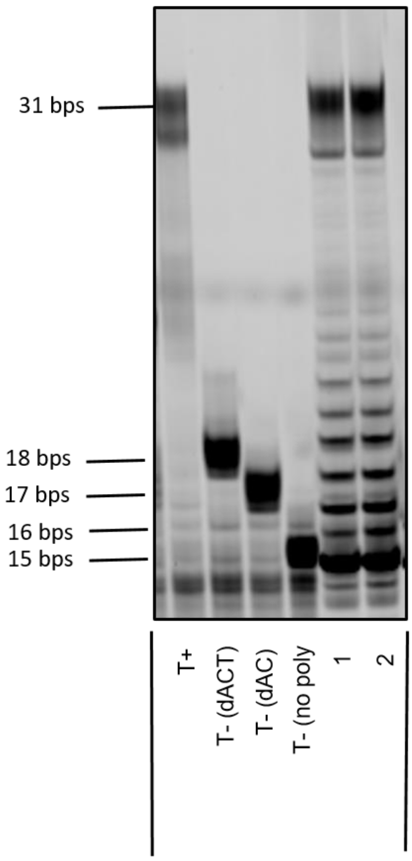

Supplementary Figure 1. Gel image (PAGE 20%) of the result of the treatment of primer **P1** with FastAP thermosensitive alkaline phosphatase prior to PEX reactions to evaluate the possibility of deblocking the 3'-phosphate group. Lanes: T+: PEX reaction with natural dNTPs and Taq polymerase using non-phosphorylated primer **P1**; T-(dACT): control reaction with non-phosphorylated, 5'-FAM-labelled primer **P1** and in the absence of dGTP; T-(dAC): control reaction with non-phosphorylated, 5'-FAM-labelled primer **P1** and in the absence of dGTP and dTTP; T-(no poly): control reaction with non-phosphorylated, 5'-FAM-labelled primer **P1** and in the absence of Taq polymerase; lane 1: treatment of 3'-phosphorylated, 5'-FAM-labelled primer **P1** with 10 U of FastAP for 1h at 37°C prior to PEX reaction; lane 2: treatment of 3'-phosphorylated, 5'-FAM-labelled primer **P1** with 30 U of FastAP for 1h at 37°C prior to PEX reaction. Conditions for PEX reactions: 50 pmoles of 5'-FAM-labelled primer **P1**-template **T1** duplex, 200 µM dNTPs, and Taq (5 U), 60°C, 1h.

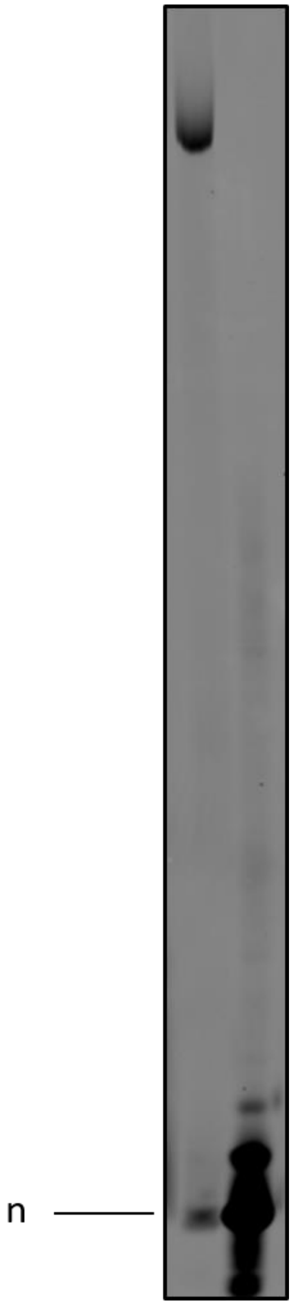

Supplementary Figure 2. Gel image (PAGE 20%) of the TdT-mediated tailing reactions with 3'-phosphorylated, 5'-FAM-labelled primer **P1** (50 pmol) after (first lane on the left) and before (second lane on the right) treatment with Quick CP (5 U, 1h at 37°C) in the presence of dTTP (200 µM). The TdT reactions were carried out at 37°C for 1h in the presence of 10 U of the polymerase and 0.25 mM Co^{2+} cofactor.

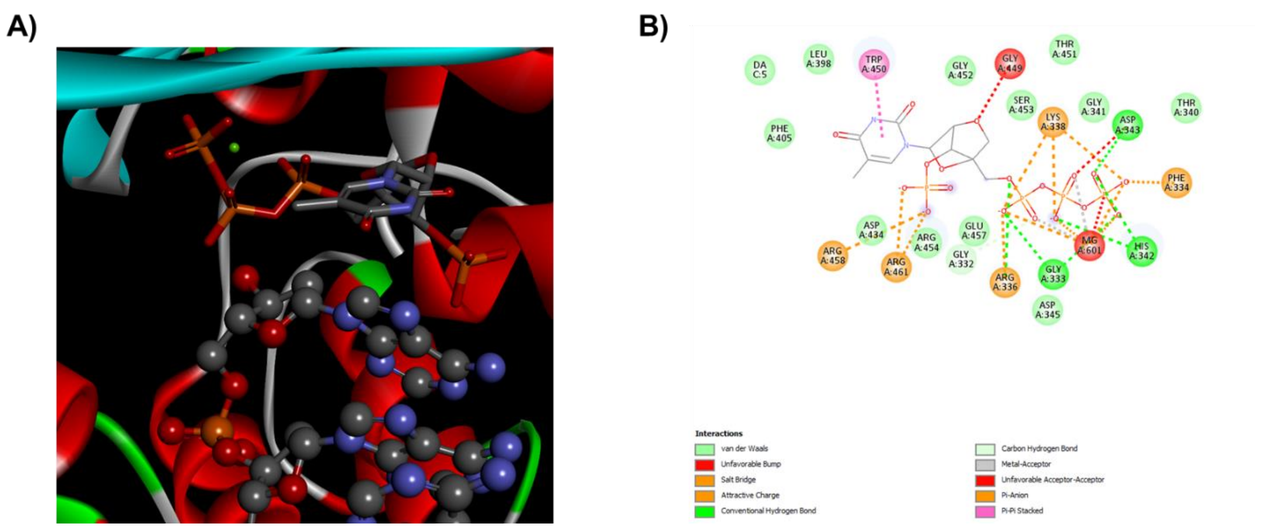

Supplementary Figure 3. A) Docking results obtained with 3′-phosphate-LNA-TTP **10** and the binary complex of mouse TdT and a ssDNA primer (PDB 4I27). B) 2D diagram of docking simulation showing the interactions between the 3′-phosphate-LNA-TTP **10** and the amino acids in the active site of the TdT polymerase.

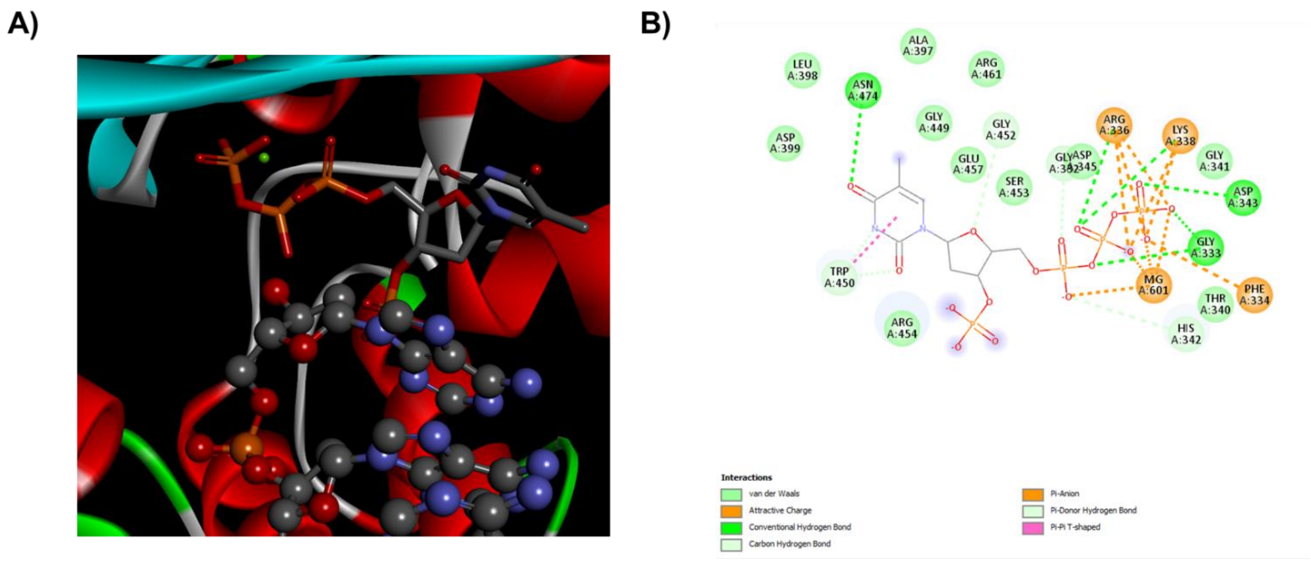

Supplementary Figure 4. A) Docking results obtained with 3′-phosphate-dTTP **5** and the binary complex of mouse TdT and a ssDNA primer (PDB 4I27). B) 2D diagram of docking simulation showing the interactions between 3’-phosphate-dTTP **5** and the amino acids in the active site of the TdT polymerase.

Template: 3’-G TAC CCG CCG TAC CCT GAC TCG AGT ACG ATC-5’
Primer: 5’-C ATG GGC GGC ATG GGA CTG AGC TCA TGC TAG-3’
15 bps

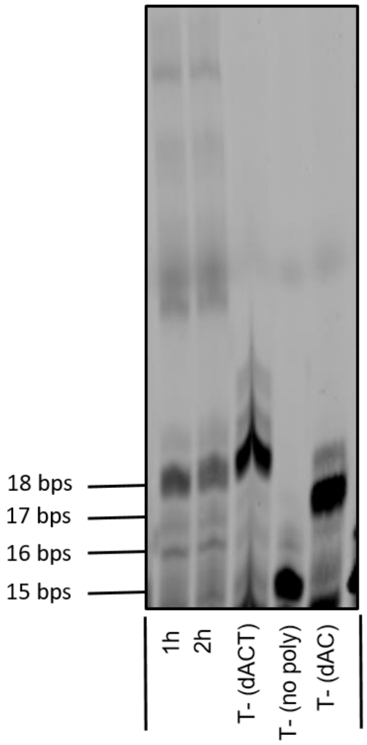

Supplementary Figure 5. Gel analysis (Page 20%) of PEX reaction with 3’-phos-dTTP **5** under optimized conditions with 5’-FAM-labelled primer **P1** (10 pmoles) and template **T1** (15 pmoles). All nucleoside triphosphates were at 200 µM final concentration. Reaction conditions: Taq (5 U), 60°C, 1h or 2h. Negative control reactions: only dATP and dCTP or dATP, dCTP, and dTTP only.

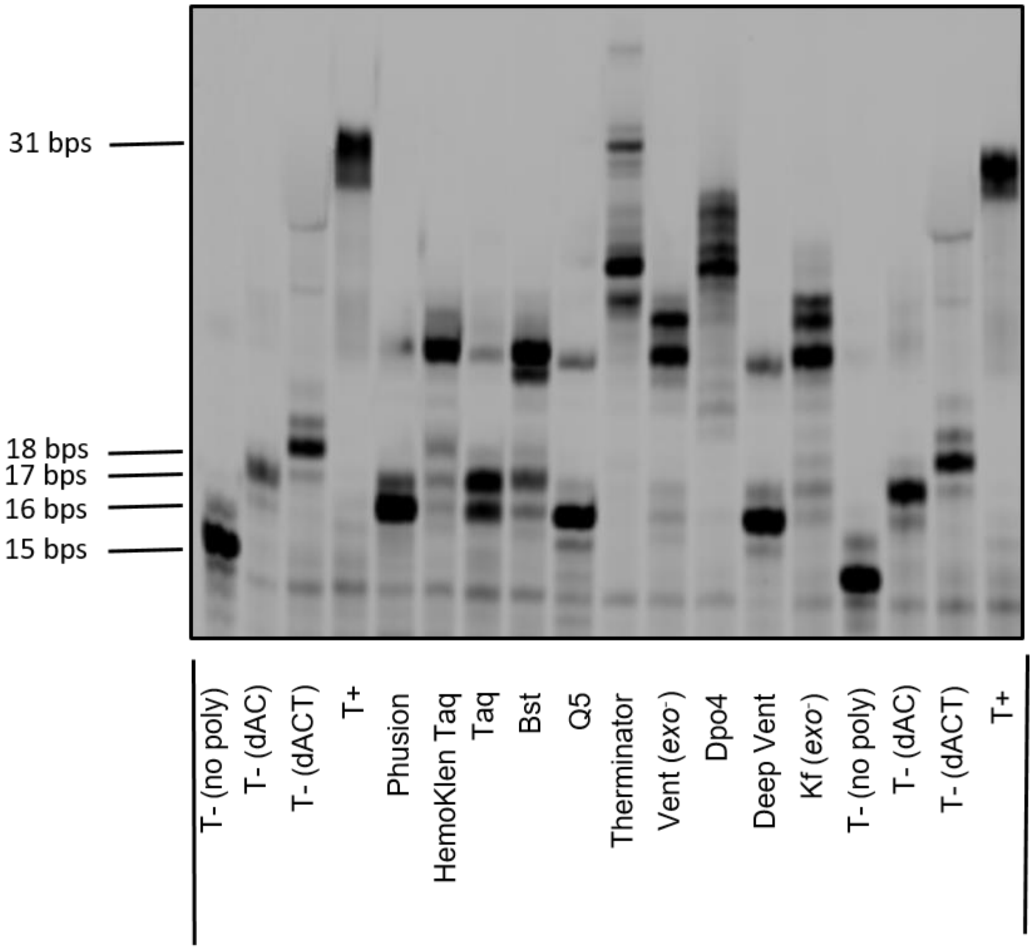

Supplementary Figure 6. Gel image (Page 20%) of PEX reactions conducted in the absence of dTTP. The three natural dNTPs were at 200 µM final concentrations and the **P1**/**T1** primer/template system was used. The following quantities of polymerases, reaction times, and temperatures were used: Phusion (2 U), Hemo Klen Taq (8 reactions), Taq (5 U), *Bst* (8 U), Q5 (2 U), Therminator (2 U), Vent (*exo*$^-$) (2 U): 60°C, 30 min; Dpo4 (2 U), Deep Vent (2 U): 55°C, 30 min; Kf (*exo*$^-$) (5 U): 37°C, 30 min. Negative control (T-): Reactions without polymerase or only dATP and dCTP or dATP, dCTP, and dTTP. Positive control (T+): reaction with all natural dNTPs and Taq polymerase. **P** represents unreacted, 5'-FAM-labelled primer.

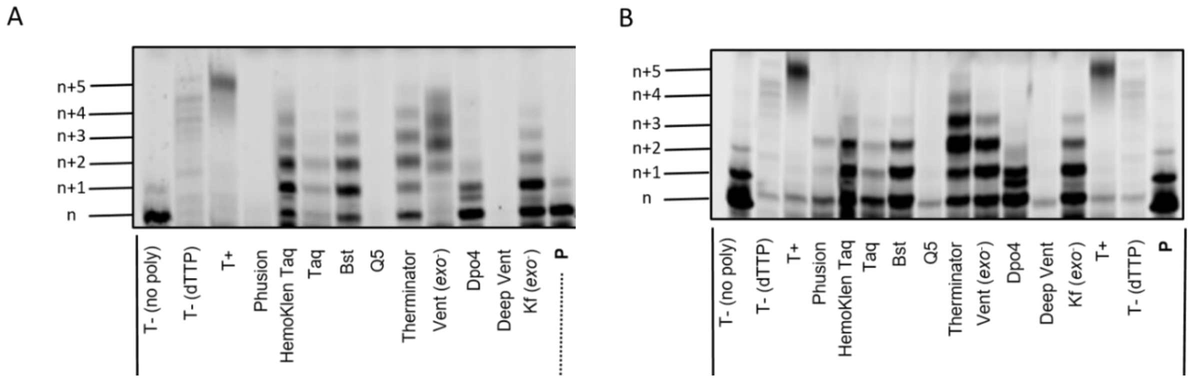

Supplementary Figure 7. Gel image (Page 20%) of PEX reactions conducted reactions with A) 3'-phos-LNA-TTP **10** and B) 3'-phos-dTTP **5** with 5'-FAM-labelled primer **P1** and template **T3**. All modified triphosphates were used at 200 µM concentrations and the following quantities of polymerases were used: Phusion (2 U), Hemo Klen Taq (8 reactions), Taq (5 U), *Bst* (8 U), Q5 (2 U), Therminator (2 U), Vent (*exo*-) (2 U): 60°C, 30 min; Dpo4 (2 U), Deep Vent (2 U): 55°C, 30 min; Kf (*exo*-) (5 U): 37°C, 30 min. Negative control (T-): Reaction without polymerase. Positive control (T+): reaction with all natural dNTPs and Taq polymerase. **P** represents unreacted, 5'-FAM-labelled primer.

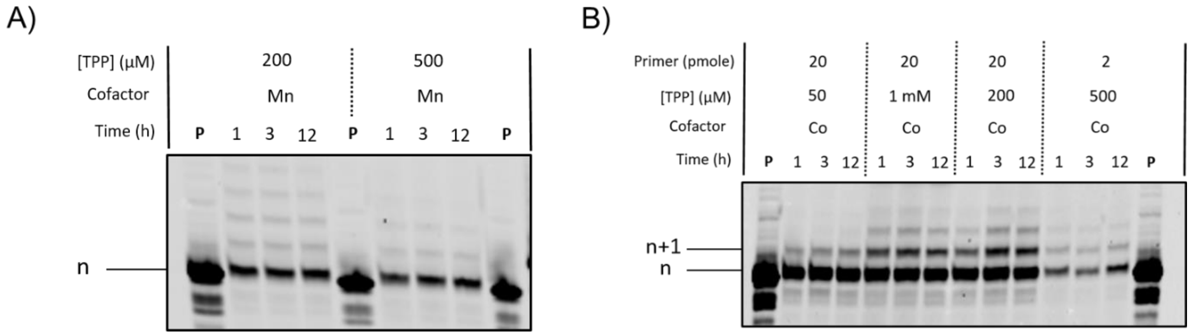

Supplementary Figure 8. Gel image (PAGE 20 %) of the TdT-mediated extension reactions with 3'-phos-LNA-TTP **10**, 5'-FAM-labelled primer **P2** (20 or 2 pmoles), and A) Mn^{2+} as cofactor and B) Co^{2+} as cofactor. Reaction mixtures contained TdT (10 U), triphosphate at given concentrations, metal cofactors at 1 mM concentration, and were incubated at 37°C for given reaction times. **P** represents unreacted, 5'-FAM-labelled primer.

A) B)

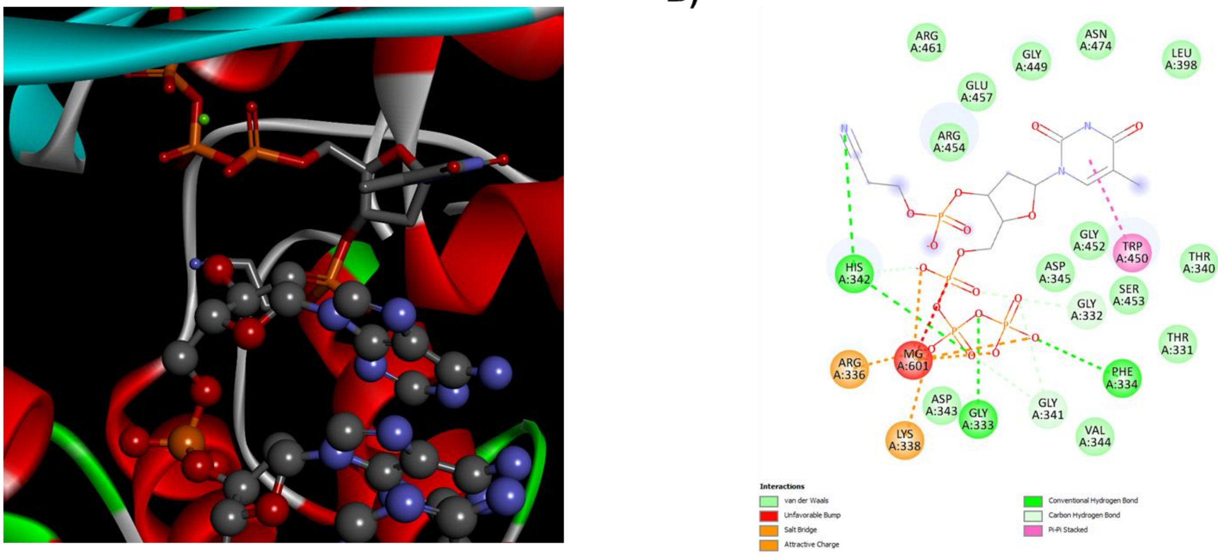

Supplementary Figure 9. A) Docking results obtained with 3′-cyanophosphate-dTTP **11** and the binary complex of mouse TdT and a ssDNA primer (PDB 4I27). B) 2D diagram of docking simulation showing the interactions between the 3′-cyanophosphate-dTTP **11** and the amino acids in the active site of the TdT polymerase.

A) B)

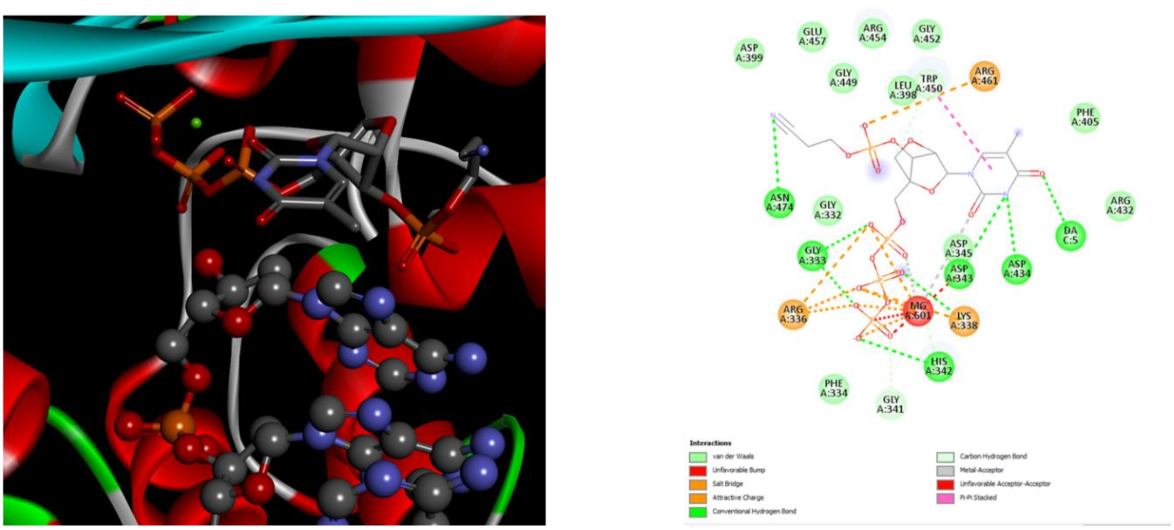

Supplementary Figure 10. A) Docking results obtained with 3′-cyanophosphate-LNA-TTP **12** and the binary complex of mouse TdT and a ssDNA primer (PDB 4I27). B) 2D diagram of docking simulation showing the interactions between the 3′-cyanophosphate-LNA-TTP **12** and the amino acids in the active site of the TdT polymerase.

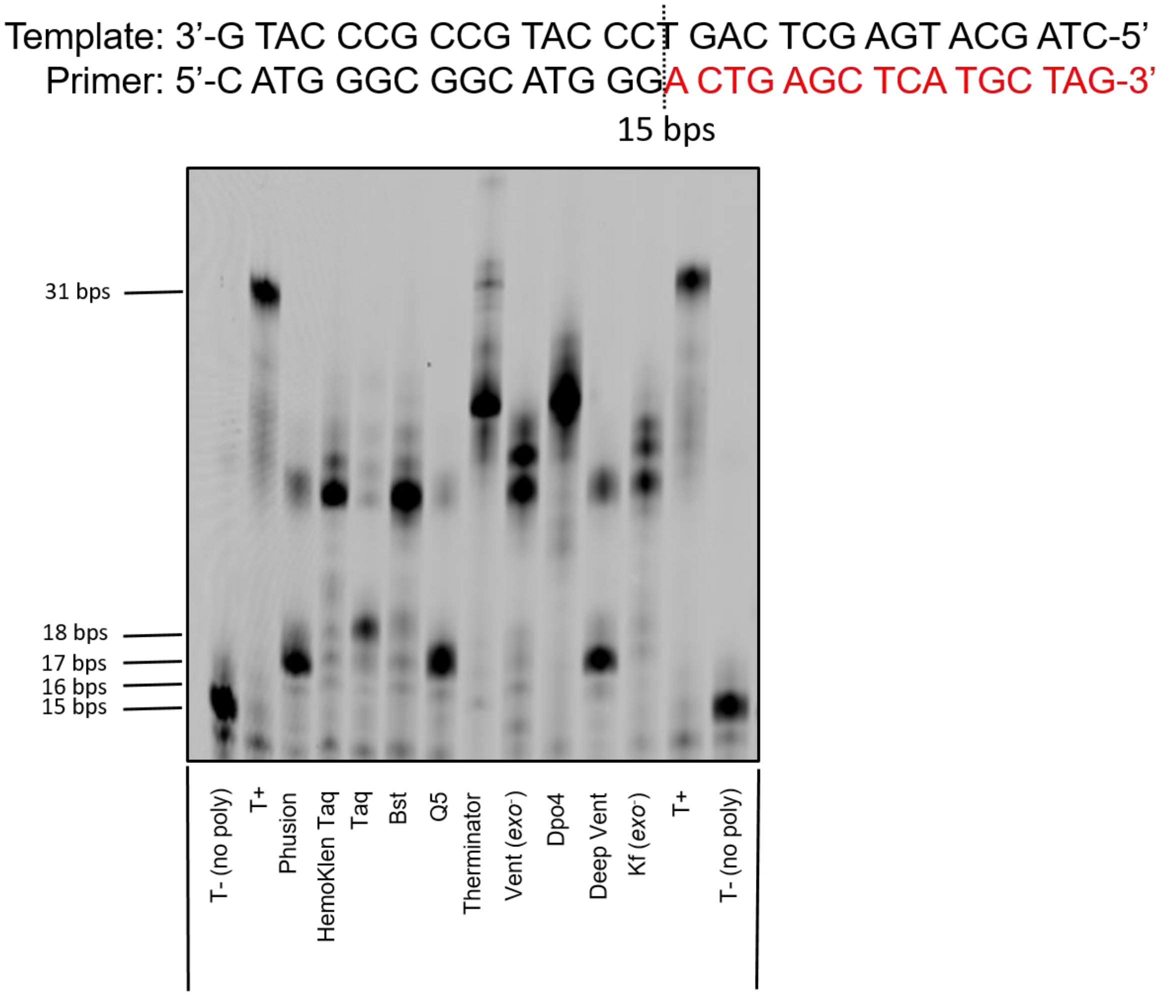

Supplementary Figure 11. Gel (PAGE 20%) analysis of PEX reactions carried out with 3'-β-cyanophosphate-dTTP **11** and primer/template **P1**/**T1**. All natural and modified triphosphates were used at 200 µM concentrations and the following quantities of polymerases were used: Phusion (2 U), Hemo Klen Taq (8 reactions), Taq (5 U), *Bst* (8 U), Q5 (2 U), Therminator (2 U), Vent (*exo*$^-$) (2 U): 60°C, 15 min; Dpo4 (2 U), Deep Vent (2 U): 55°C, 15 min; Kf (*exo*$^-$) (5 U): 37°C, 15 min. Negative control (T-): Reactions without polymerase. Positive control (T+): reaction with all natural dNTPs and Taq polymerase.

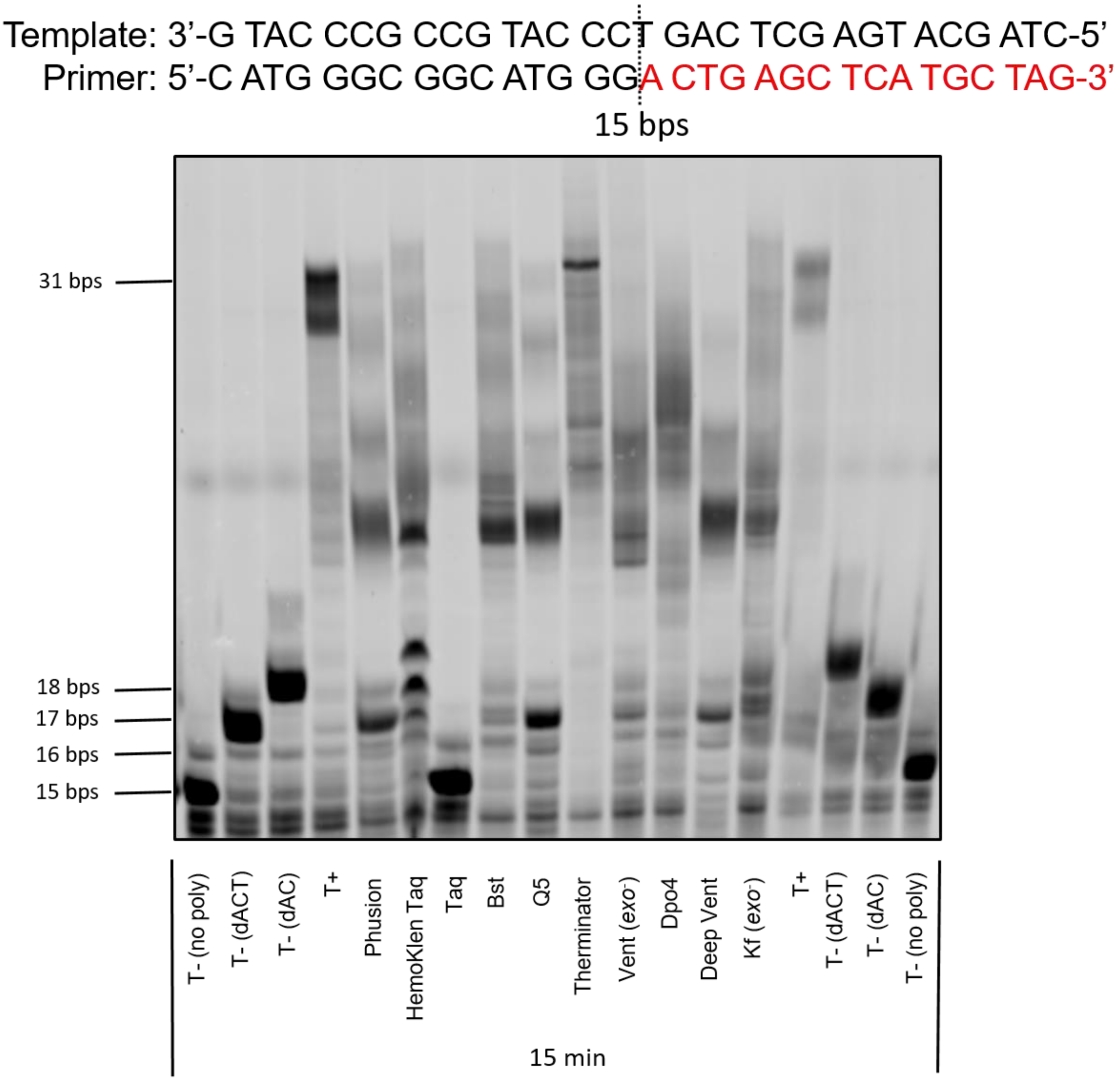

Supplementary Figure 12. Gel (PAGE 20%) analysis of PEX reactions carried out with 3'-β-cyanophosphate-LNA-TTP **12** and primer/template **P1**/**T1**. All natural and modified triphosphates were used at 200 µM concentrations and the following quantities of polymerases were used: Phusion (2 U), Hemo Klen Taq (8 reactions), Taq (5 U), *Bst* (8 U), Q5 (2 U), Therminator (2 U), Vent (*exo*$^-$) (2 U): 60°C, 30 min; Dpo4 (2 U), Deep Vent (2 U): 55°C, 30 min; Kf (*exo*$^-$) (5 U): 37°C, 30 min. Negative control(T-): Reactions without polymerase or only with dATP and dCTP or dATP, dCTP, and dTTP. Positive control (T+): reaction with all natural dNTPs and Taq polymerase.

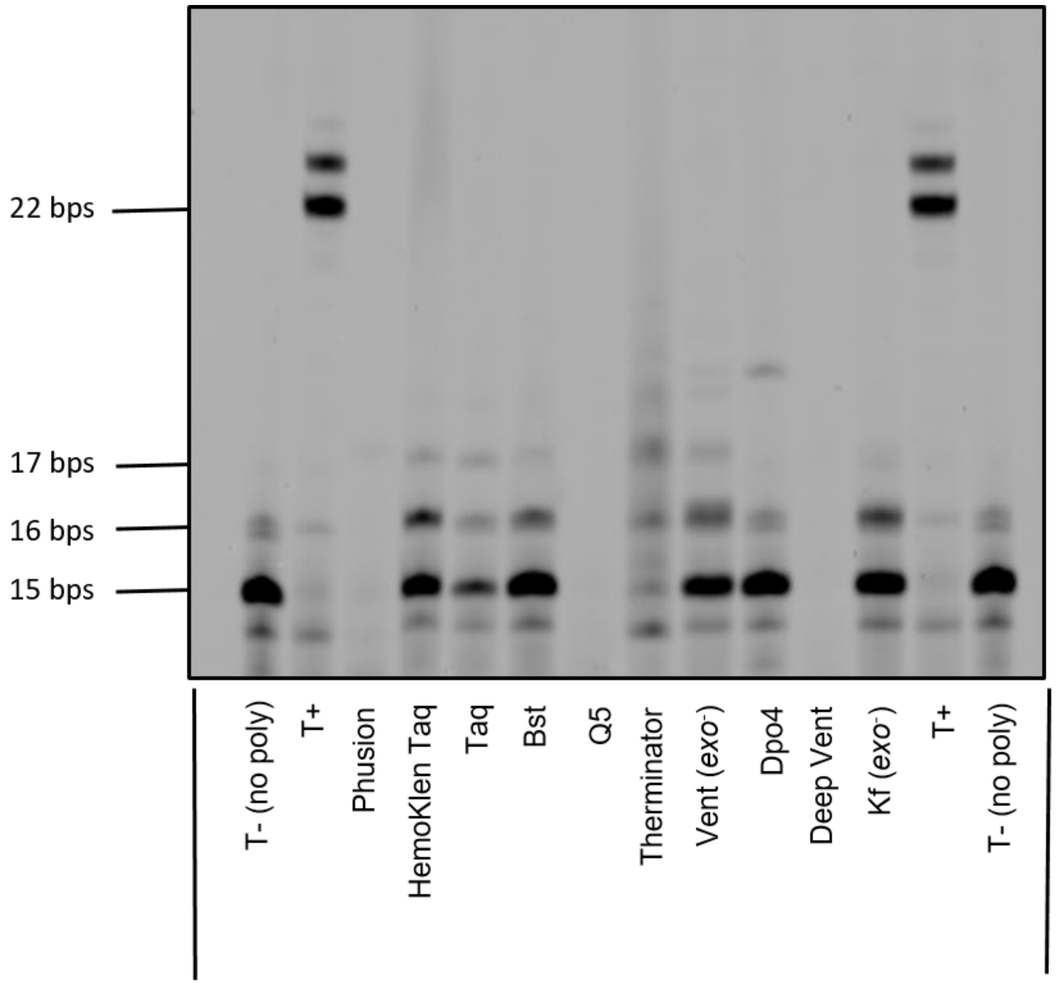

Supplementary Figure 13. Gel (PAGE 20%) analysis of PEX reactions carried out with 3'-β-cyanophosphate-dTTP **11** and primer/template **P1**/**T1**. Modified triphosphate was used at 200 µM concentrations and the following quantities of polymerases were used: Phusion (2 U), Hemo Klen Taq (8 reactions), Taq (5 U), *Bst* (8 U), Q5 (2 U), Therminator (2 U), Vent (*exo*$^{-}$) (2 U): 60°C, 60 min; Dpo4 (2 U), Deep Vent (2 U): 55°C, 60 min; Kf (*exo*$^{-}$) (5 U): 37°C, 60 min. Negative control(T-): Reactions without added polymerase. Positive control (T+): reaction with all natural dNTPs and Taq polymerase.

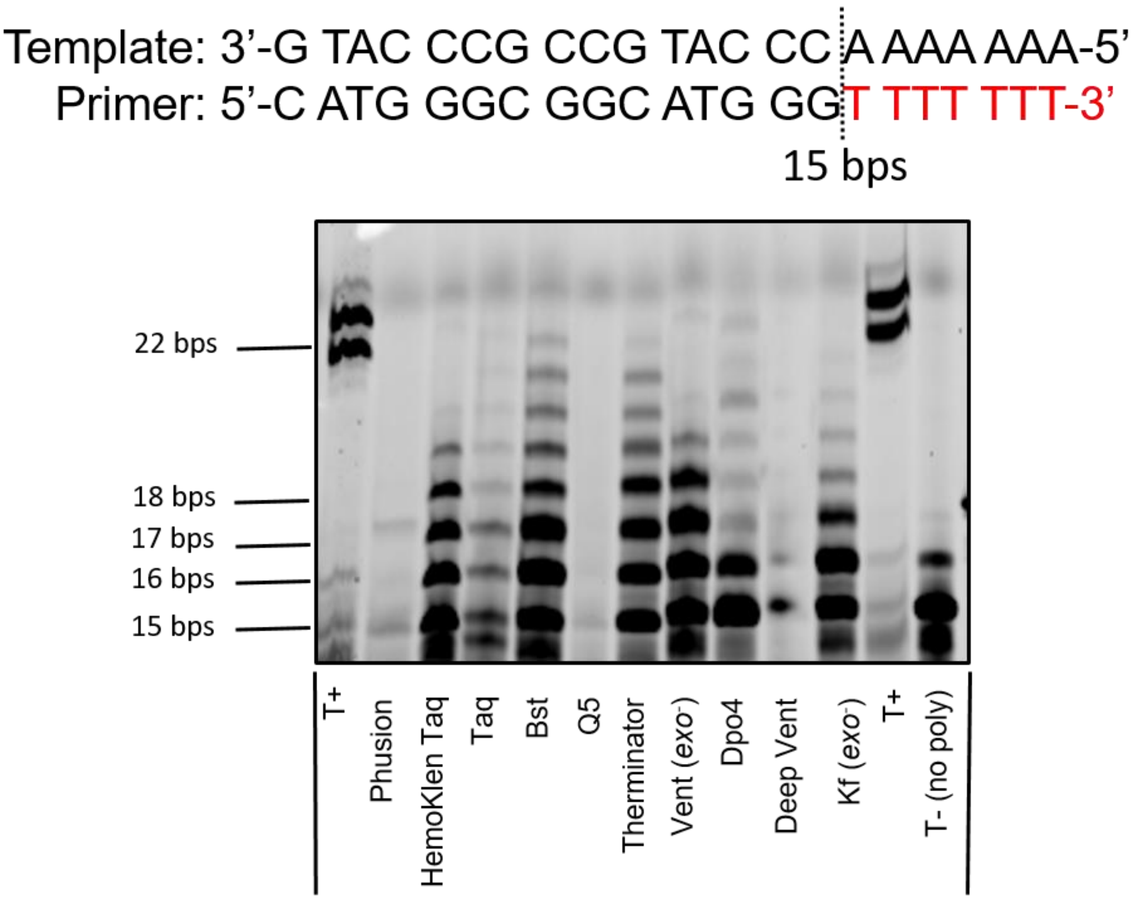

Supplementary Figure 14. Gel (PAGE 20%) analysis of PEX reactions carried out with 3'-β-cyanophosphate-LNA-TTP **12** and primer/template **P1**/**T2**. Modified triphosphate was used at 200 µM concentrations and the following quantities of polymerases were used: Phusion (2 U), Hemo Klen Taq (8 reactions), Taq (5 U), *Bst* (8 U), Q5 (2 U), Therminator (2 U), Vent (*exo*-) (2 U): 60°C, 60 min; Dpo4 (2 U), Deep Vent (2 U): 55°C, 60 min; Kf (*exo*-) (5 U): 37°C, 60 min. Negative control(T-): Reactions without added polymerase. Positive control (T+): reaction with all natural dNTPs and Taq polymerase.

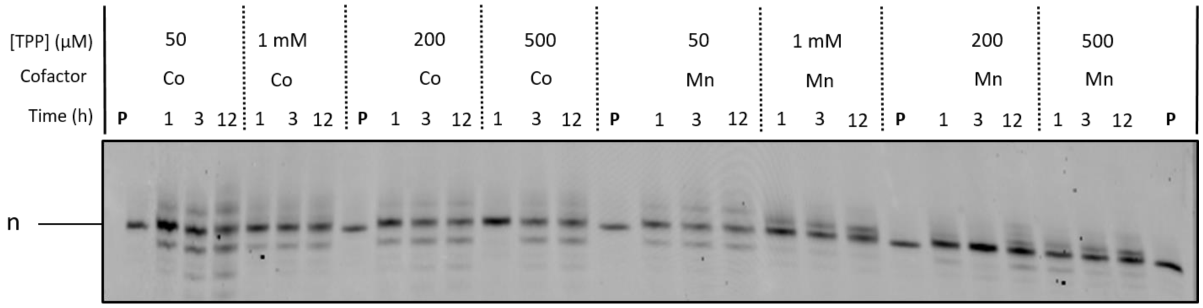

Supplementary Figure 15. Gel image (PAGE 20 %) of the TdT-mediated extension reactions with 3'-β-cyanophosphate-dTTP **11** and primer **P2** (20 pmoles), and Mn^{2+} (1 mM) or Co^{2+} (0.25 mM) as cofactors. Reaction mixtures contained TdT (10 U), triphosphate at given concentrations, and were incubated at 37°C for given reaction times. **P** represents unreacted, 5'-FAM-labelled primer.

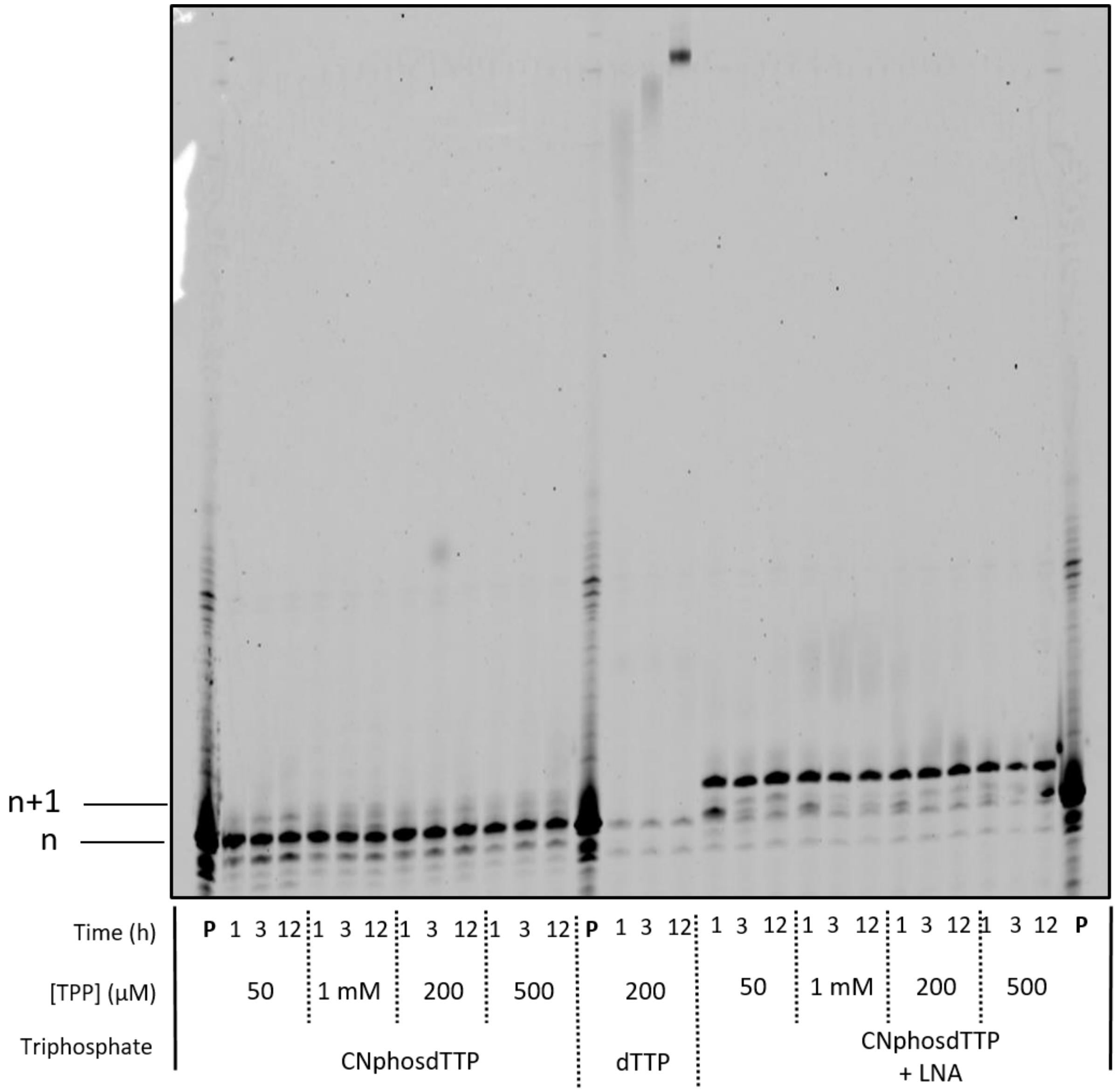

Supplementary Figure 16. Gel image (PAGE 20 %) of the TdT-mediated extension reactions with 3'-β-cyanophosphate-dTTP **11** (CNphosdTTP in the caption) alone and with added LNA-TTP (CNphosdTTP+LNA in the caption). Reaction mixtures contained given concentrations of nucleoside triphosphates, TdT (10 U), 20 pmoles of 5'-FAM-labelled primer **P2**, and were incubated at 37°C for given reaction times. **P** represents unreacted, 5'-FAM-labelled primer.

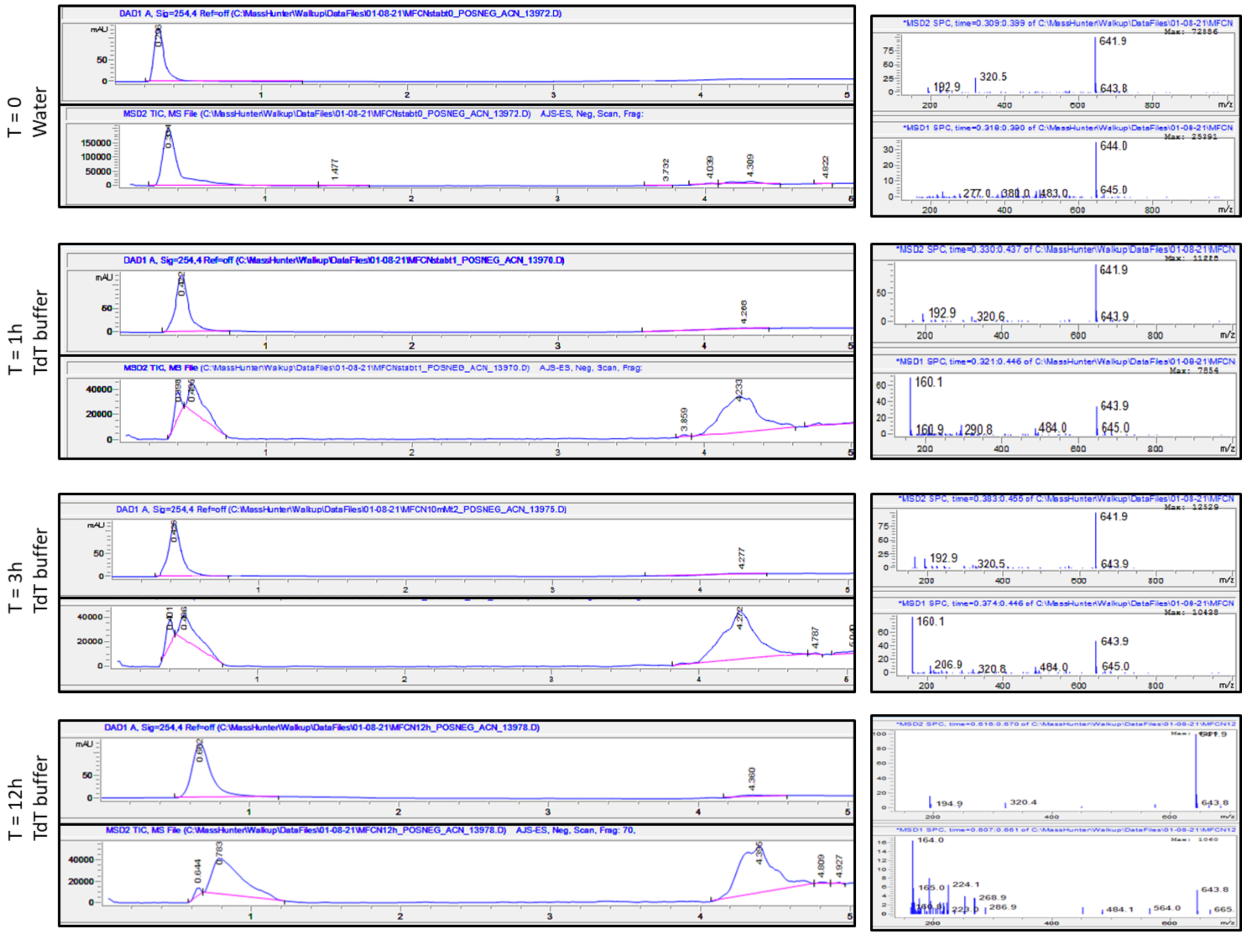

Supplementary Figure 17. LCMS analysis of the stability of nucleotide **11** in TdT buffer at 37°C. Nucleotide **11** (10 mM) was incubated in TdT buffer at 37°C for given amounts of time. Calculated *m*/*z* for **11**: 641.98.

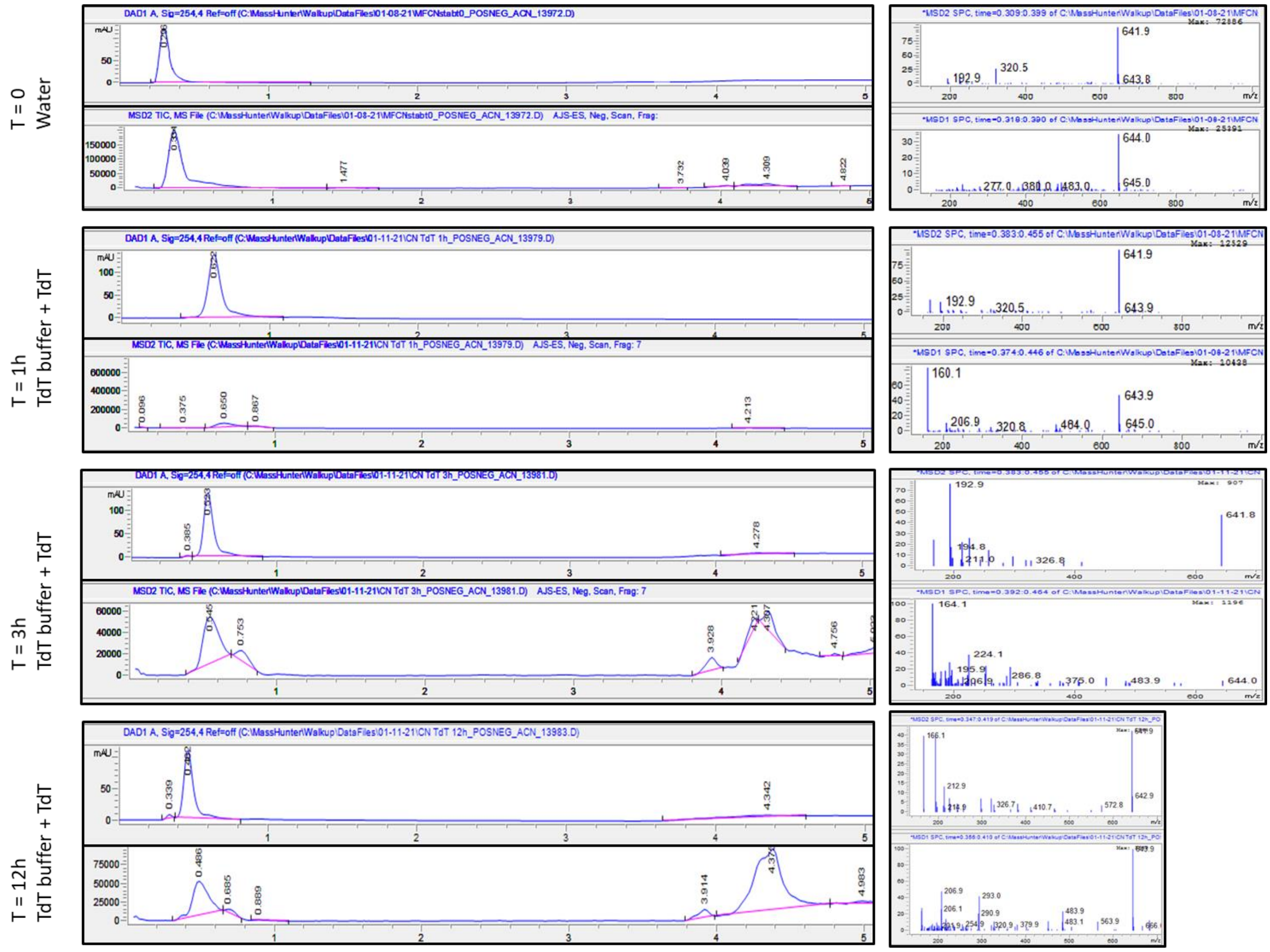

Supplementary Figure 18. LCMS analysis of the stability of nucleotide **11** in TdT buffer at 37°C. Nucleotide **11** (10 mM) was incubated with TdT (20 U) in TdT buffer at 37°C for given amounts of time. Calculated *m*/*z* for **11**: 641.98.

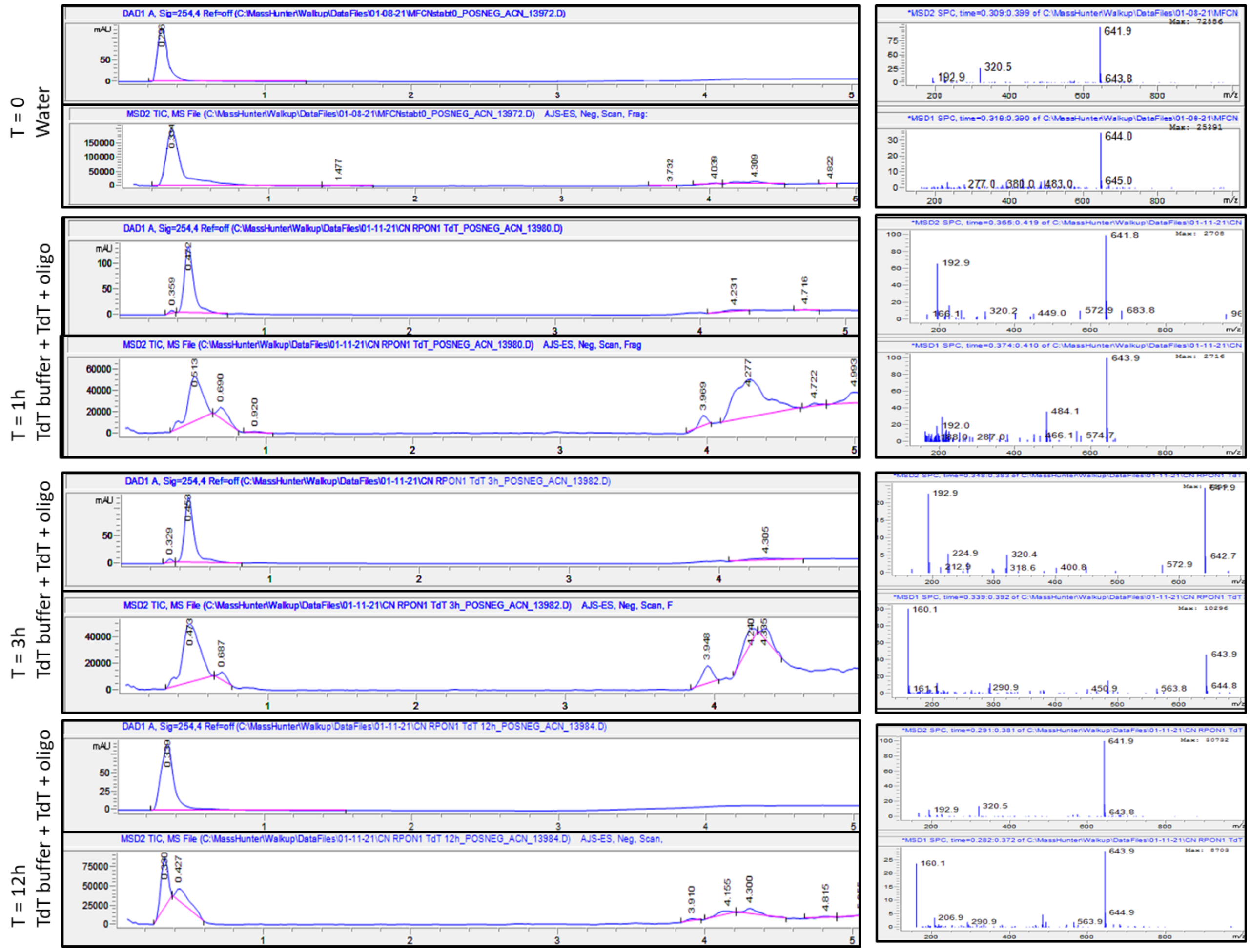

Supplementary Figure 19. LCMS analysis of the stability of nucleotide **11** in TdT buffer at 37°C. Nucleotide **11** (10 mM) was incubated with TdT (20 U), and primer **P2** (160 pmoles) in TdT buffer at 37°C for given amounts of time. Calculated *m*/*z* for **11**: 641.98.

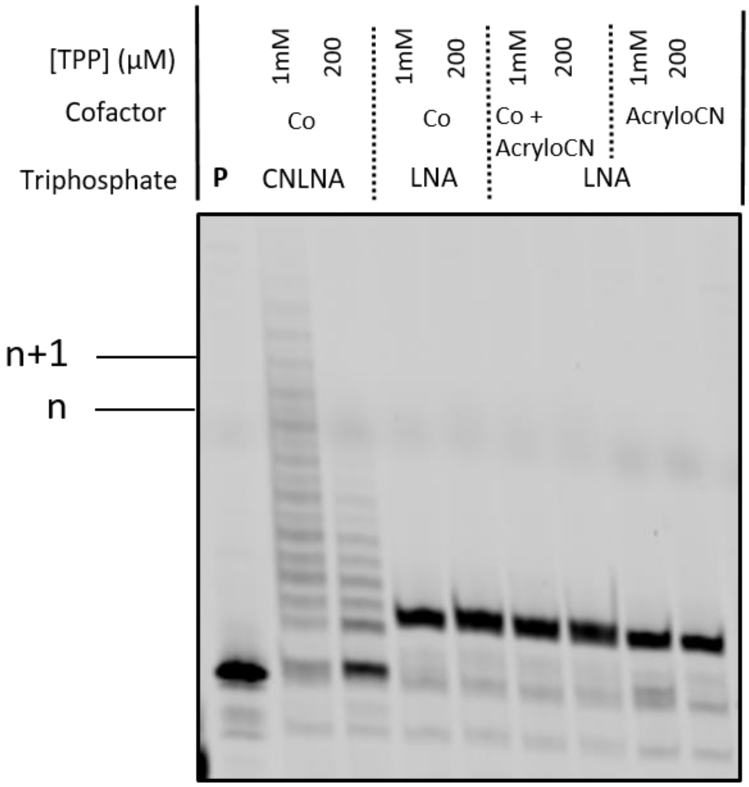

Supplementary Figure 20. Gel image (PAGE 20 %) of the TdT-mediated extension reactions with 3'-β-cyanophosphate-LNA-TTP **12** (CNLNA in the caption), LNA-TTP (LNA in the caption), and LNA-TTP treated with acrylonitrile prior to the reaction (AcryloCN in the caption). Reaction mixtures contained given concentrations of nucleoside triphosphates, TdT (10 U), 20 pmoles of primer **P2**, and were incubated at 37°C for 60 min. **P** represents unreacted, 5'-FAM-labelled primer.

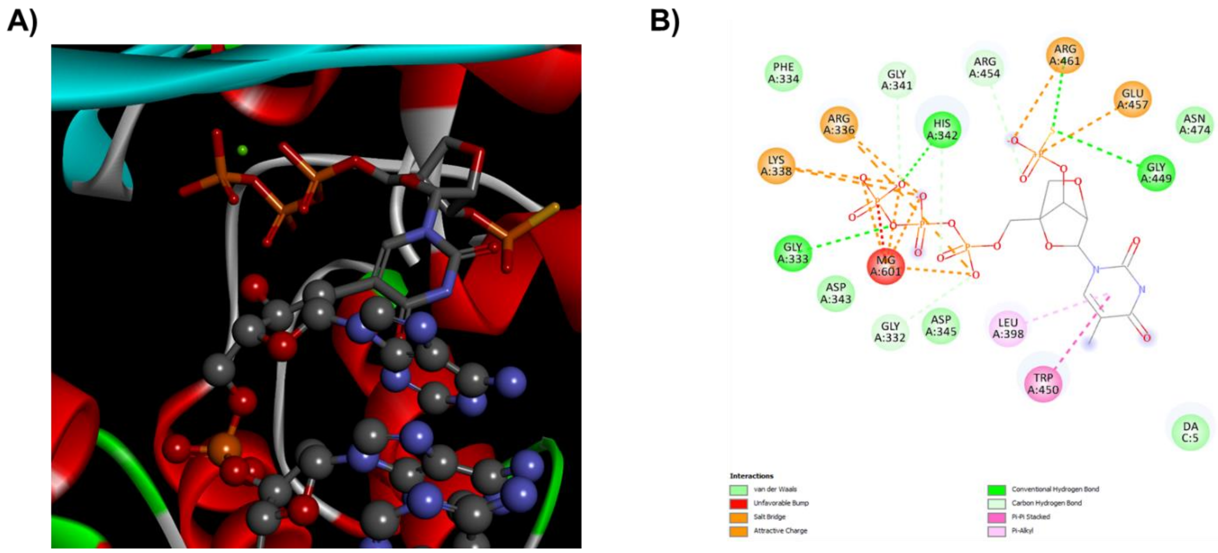

Supplementary Figure 21. A) Docking results obtained with 3′-thiophosphate-LNA-TTP **15** and the binary complex of mouse TdT and a ssDNA primer (PDB 4I27). B) 2D diagram of docking simulation showing the interactions between the 3′-thiophosphate-LNA-TTP **15** and the amino acids in the active site of the TdT polymerase.

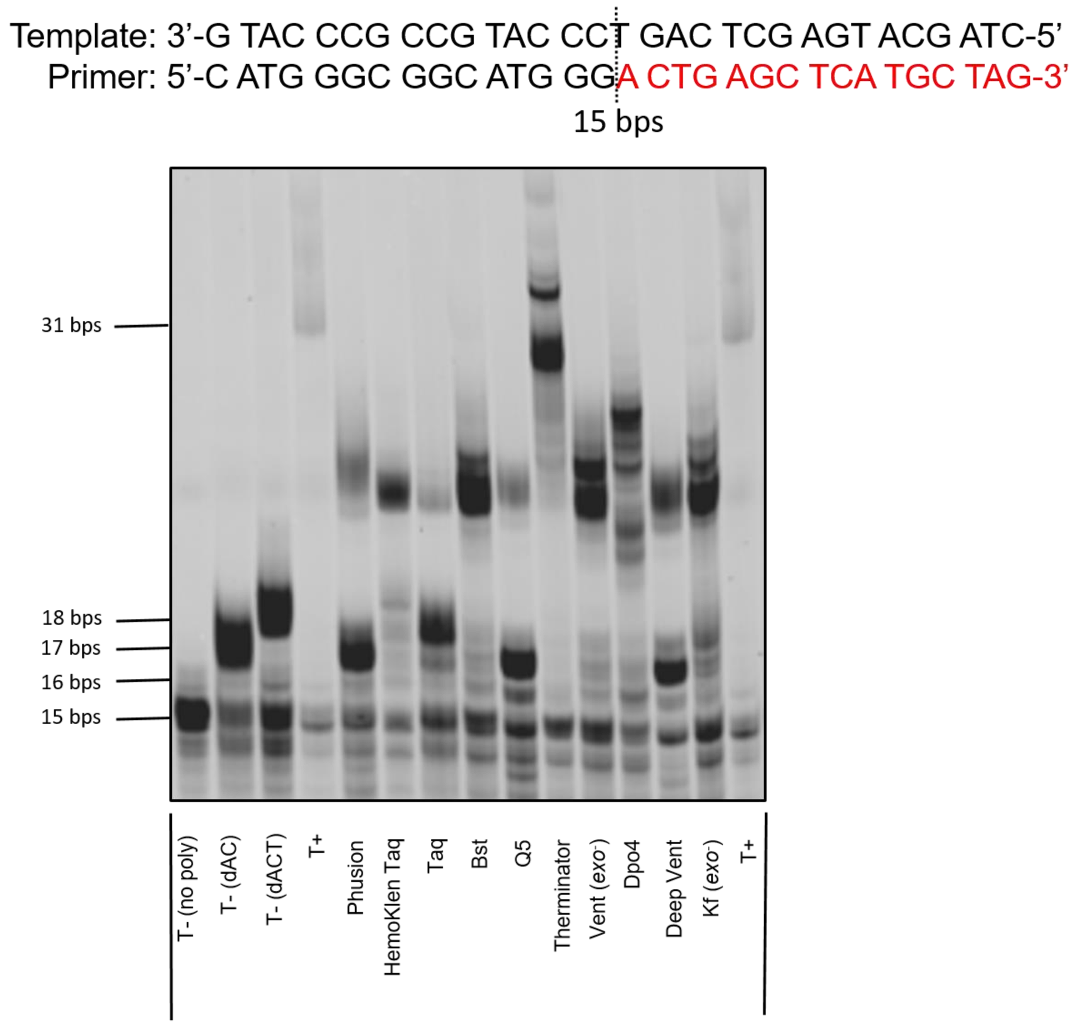

Supplementary Figure 22. Gel (PAGE 20%) analysis of PEX reactions carried out with 3′-thio-3'-β-cyanoethyl-phosphate-LNA-TTP **16** and primer/template **P1/T1**. Reactions contained natural and modified triphosphates at 200 µM. The following quantities of polymerases were used: Phusion (2 U), Hemo Klen Taq (8 reactions), Taq (5 U), Bst (8 U), Q5 (2 U), Therminator (2 U), Vent (exo-) (2 U): 60°C, 30 min; Dpo4 (2 U), Deep Vent (2 U): 55°C, 30 min; Kf (exo-) (5 U): 37°C, 30 min. Negative control (T-): No polymerase added to the mixtures or reactions with only dATP and dCTP or dATP, dCTP, and dTTP only. Positive control (T+): with all natural nucleotides and Taq polymerase. All reactions were incubated at adequate reaction temperatures for 1h. **P** represents unreacted, 5'-FAM-labelled primer.

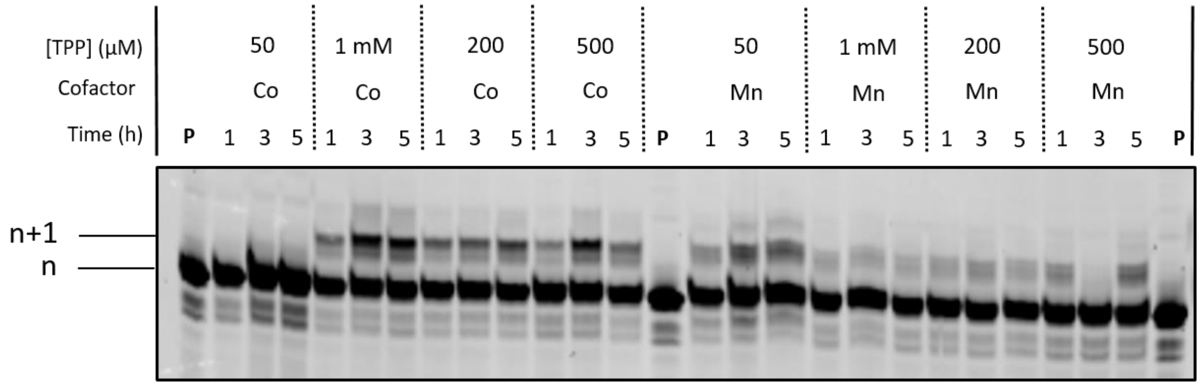

Supplementary Figure 23. Gel image (PAGE 20 %) of the TdT-mediated extension reactions with 3′-thio-3'-β-cyanoethyl-phosphate-LNA-TTP **16**. Reaction mixtures contained given

concentrations of nucleoside triphosphates, TdT (10 U), 20 pmoles of primer **P2**, and were incubated at 37°C for given reaction times. **P** represents unreacted, 5’-FAM-labelled primer.

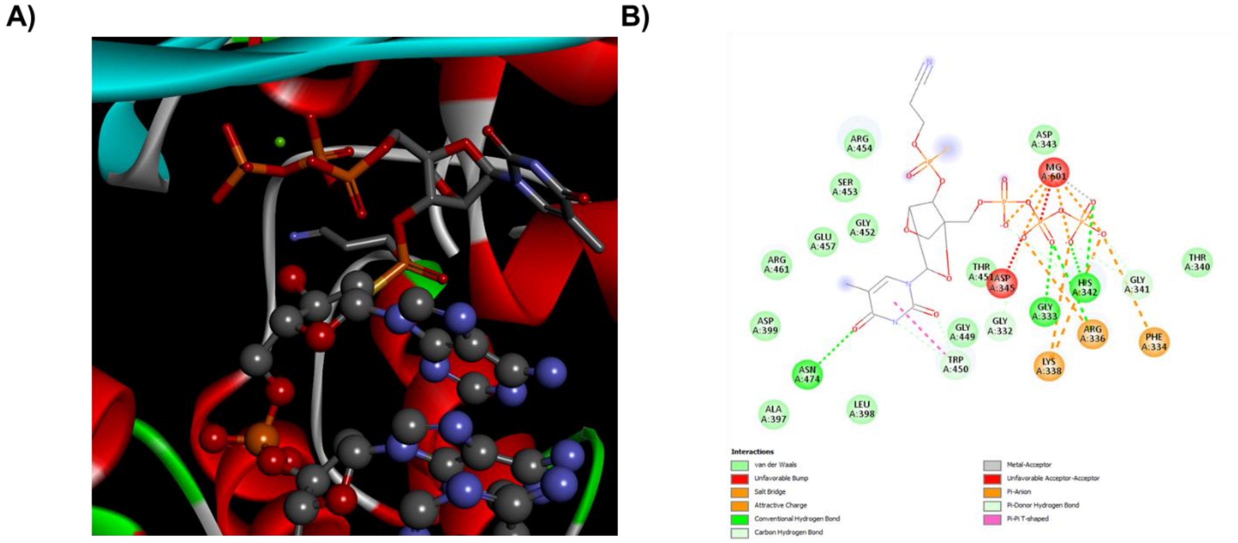

Supplementary Figure 24. A) Docking results obtained with 3′-thio-3’-β-cyanoethyl-phosphate-LNA-TTP **16** and the binary complex of mouse TdT and a ssDNA primer (PDB 4I27). B) 2D diagram of docking simulation showing the interactions between the 3′-thio-3’-β-cyanoethyl-phosphate-LNA-TTP **16** and the amino acids in the active site of the TdT polymerase.

2. LC-MS characterizations of products from PEX reactions

Supplementary Note 1:

Method description:

UHPLC system used: Thermo Vanquish UPLC system, binary pump, column oven and, DAD UV detector; column used: Waters Acquity Oligo BEH C18 50x2.1mm, 1.7 µm, 130Å Part No 186003949; column oven: 80°C.

Buffers and LC program:

Mobile Phase A: 200 mM Hexafluoroisopropanol (HFIP) + 10 mM hexylamine (HA) in water (21.1 ml HFIP + 1.324 ml HA + 980ml water; mobile Phase B: 50 % methanol + 50 % acetonitrile (v/v); flow rate: 0.4 ml/min

Gradient table:

Time (min)	Mobile Phase A (%)	Mobile Phase B (%)
0	91	9
1	91	9
1.5	83	17
23	68	32
28	5	95
28.5	91	9
3	91	9

Injection volume: 1 µL

Detection: UV 260 nm, BW 4 nm

Mass Spectrometer: Thermo Lumos Fusion Orbitrap Negative Ion Mode (*m*/*z*) 600-2000; resolution 120000 H-ESI source, spray voltage -3000V, ion transfer tube temp 375°C, vaporizer temp 200°C, sheat gas 40, aux gas 5, and sweep gas 2.

Sample preparation: The sample, if solid, is diluted to a conc. of 1 mg/ml. Diluent: with 90 % water + 10 % methanol/acetonitrile (9/1). In case the sample is submitted in solution the sample dilution is depending the estimated sample concentrationand the injection volume is adapted accordingly. The injection volume may range from 0.1 to 20µL max.

Data evaluation: Thermo Foundation Xcalibur QualBrowser and Novatia LLC ProMass HR for Xcalibur. Listing (printouts) and peak annotation as given by ProMass HR. Please refer to https://www.enovatia.com/

a) Characterization of PEX products stemming from the reaction obtained with the Taq polymerase and 3’-phos-dTTP **5** on the **P1/T1** system (see Table 1 of manuscript):

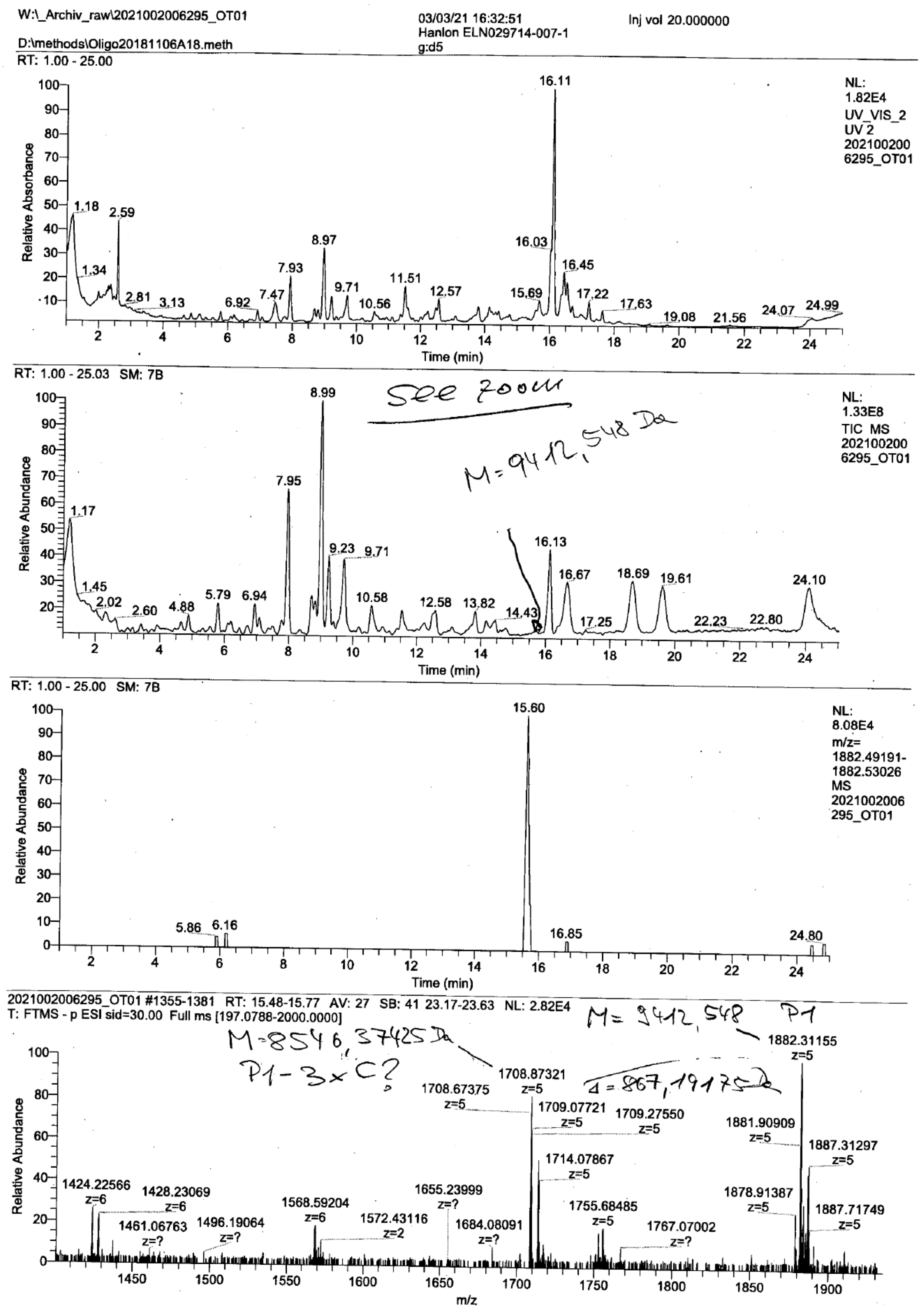

Supplementary Figure 25. LCMS profiles and ESI-MS analysis.

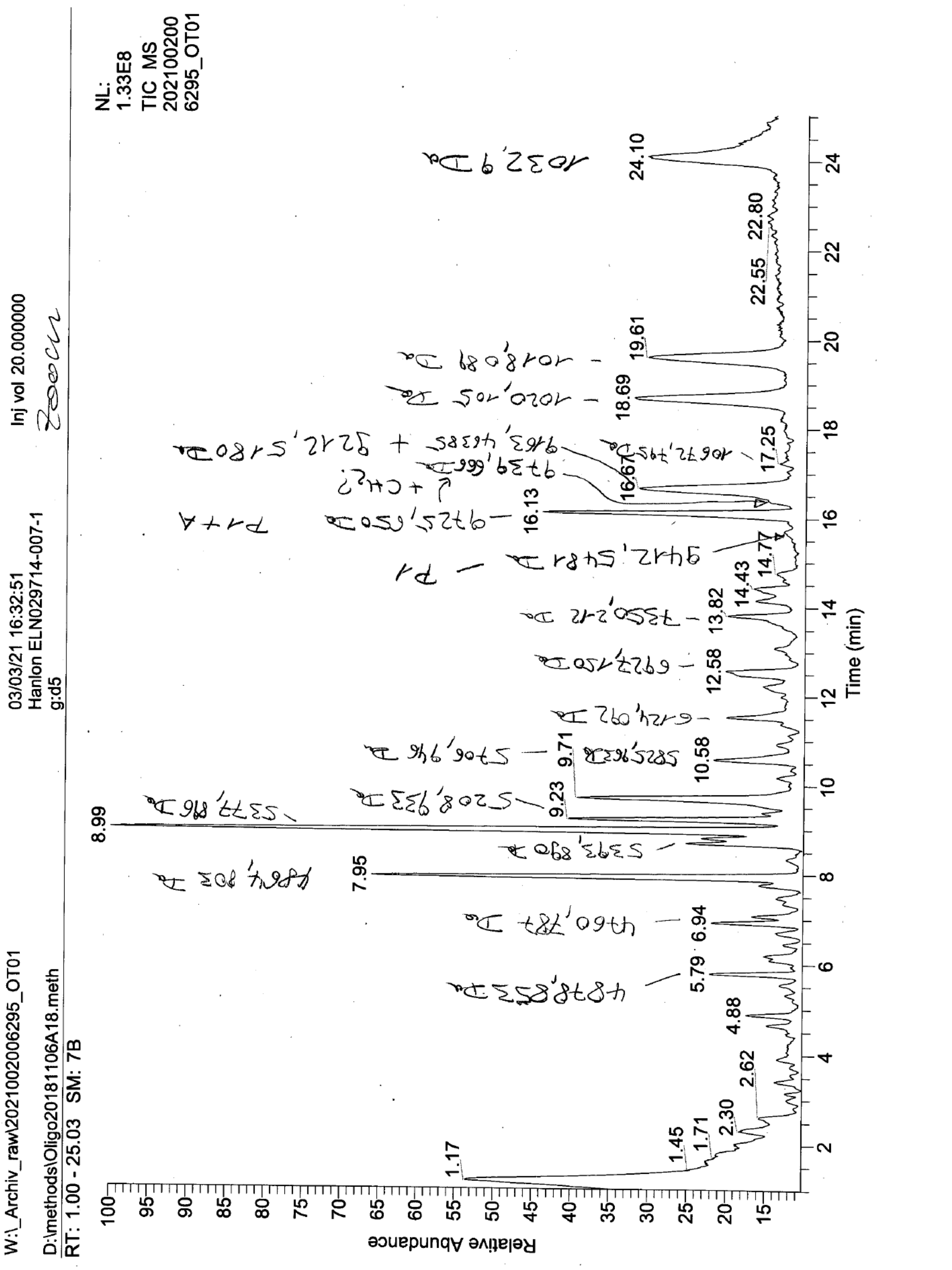

Supplementary Figure 26. Close-up view of the LCMS analysis.

In the range of 1-14 minutes, only compounds with masses up to 7350 Da elute.

Retention time	Mass	Proposal
15.80 min	9412.548 Da	Template Strand
16.13 min	9725.650 Da	Template Strand + A
16.30 min	9739.666 Da	?
16.67 min	9163.464 Da	?
16.67 min	9212.518 Da	?
17.25 min	10672.795 Da	?

In the area after 18 minutes, only compounds with masses below 1100 Da elute.

Supplementary Figure 27. Summary of results

b) Characterization of PEX products stemming from the reaction obtained with the Therminator and 3'-phos-LNA-TTP **10** on the **P1**/**T1** system (see Table 1 of manuscript):

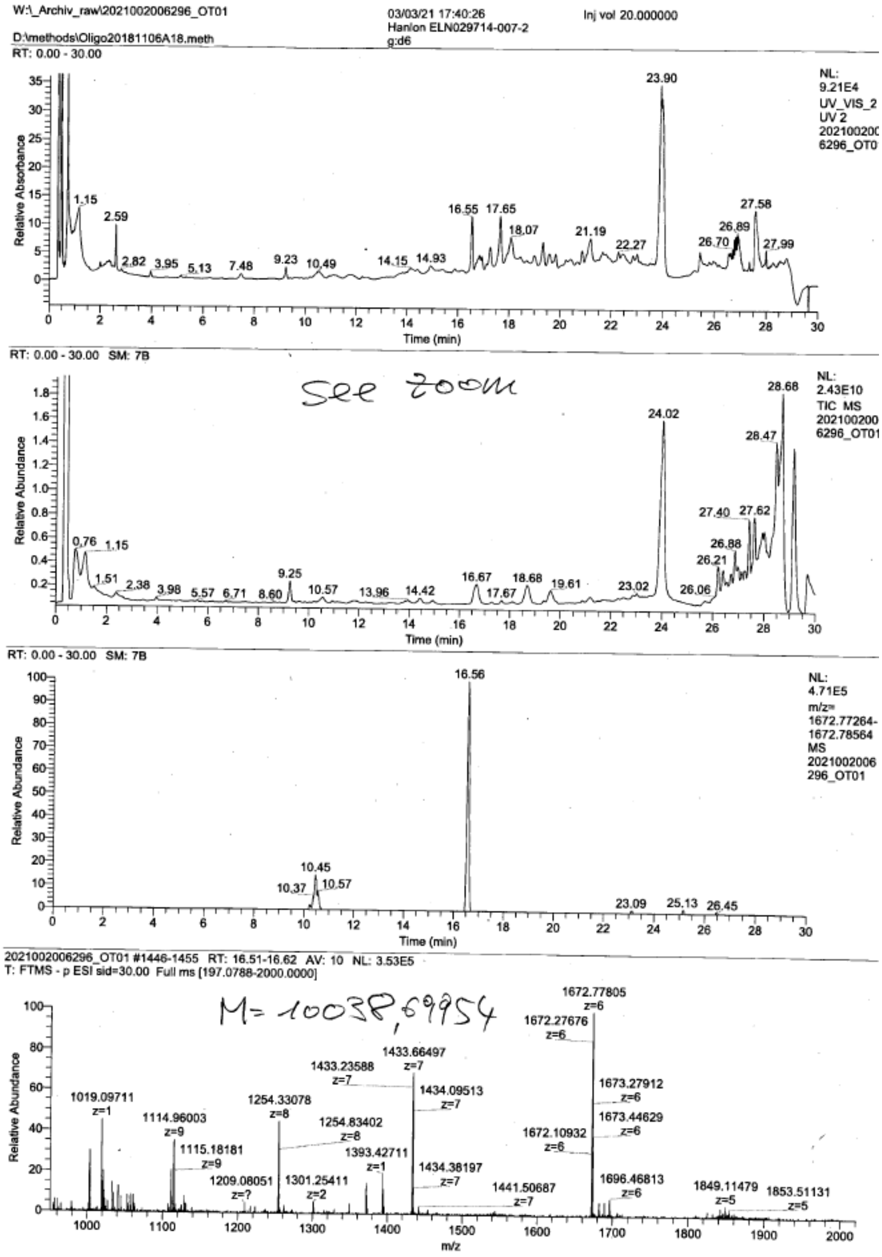

Supplementary Figure 28. LCMS profiles and ESI-MS analysis.

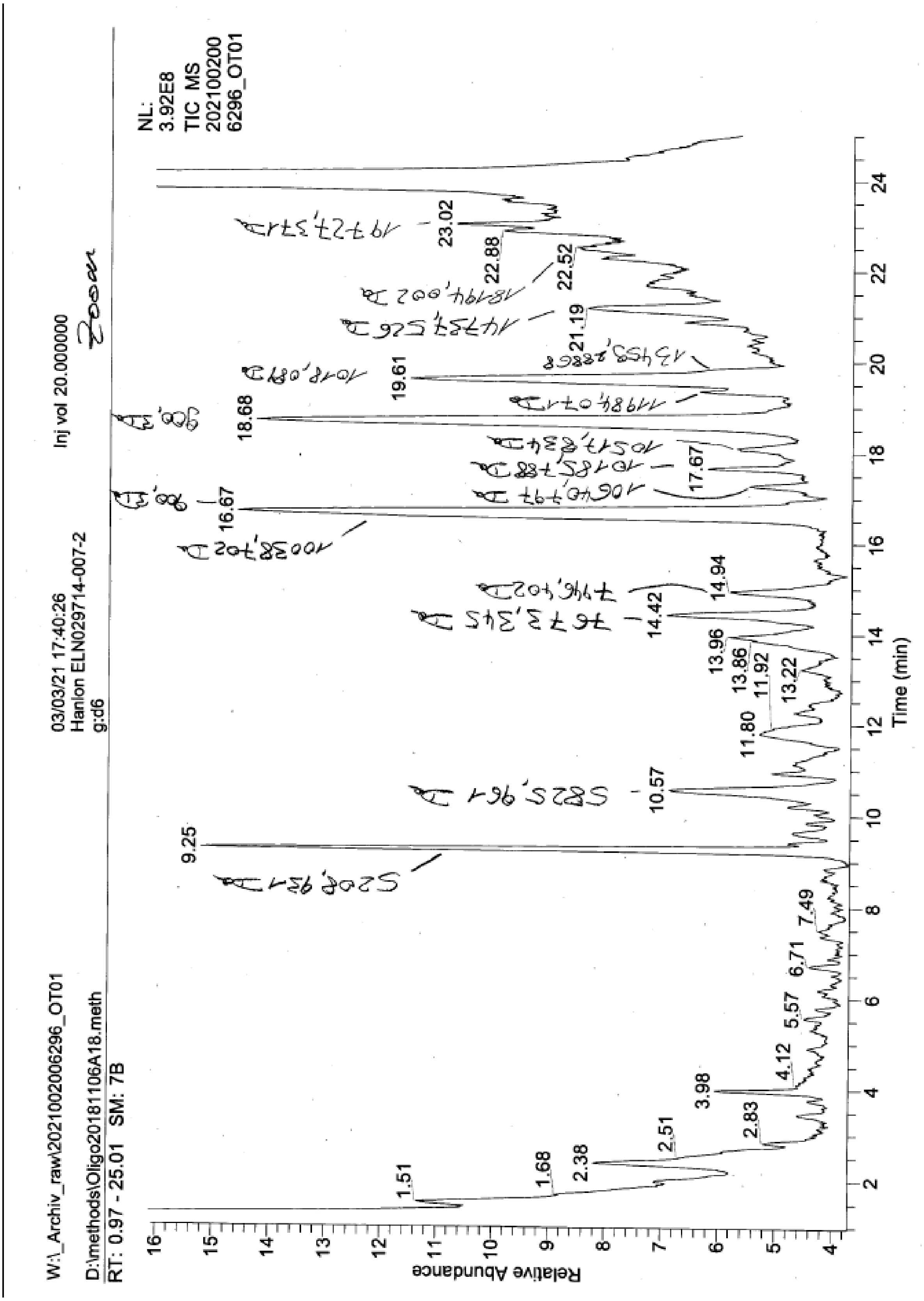

Supplementary Figure 29. Close-up view of the LCMS analysis.

In the range of 1-14 minutes, only compounds with masses up to 7350 Da elute.

Retention time	Mass	
14.42 min	7673.345 Da	
14.94 min	7946.402 Da	
16.58 min	10038.702 Da	
16.67 min	900.3 Da	
17.28 min	10640.797 Da	
17.67 min	10185.788 Da	Differenz = 332.046 fits for LNA T
18.11 min	10517.834 Da	
18.68 min	900.3 Da	
19.36 min	11984.071 Da	
19.61 min	1018.089 Da	
19.84 min	13459.289 Da	
21.19 min	14737.526 Da	
22.52 min	18194.002 Da	
23.02 min	19727.371 Da	

Supplementary Figure 30. Summary of results.

c) Characterization of PEX products stemming from the reaction obtained with the 3'-phos-dTTP **5** and Vent (*exo*$^{-}$) on the **P1**/**T3** system (see Table 2 of manuscript):

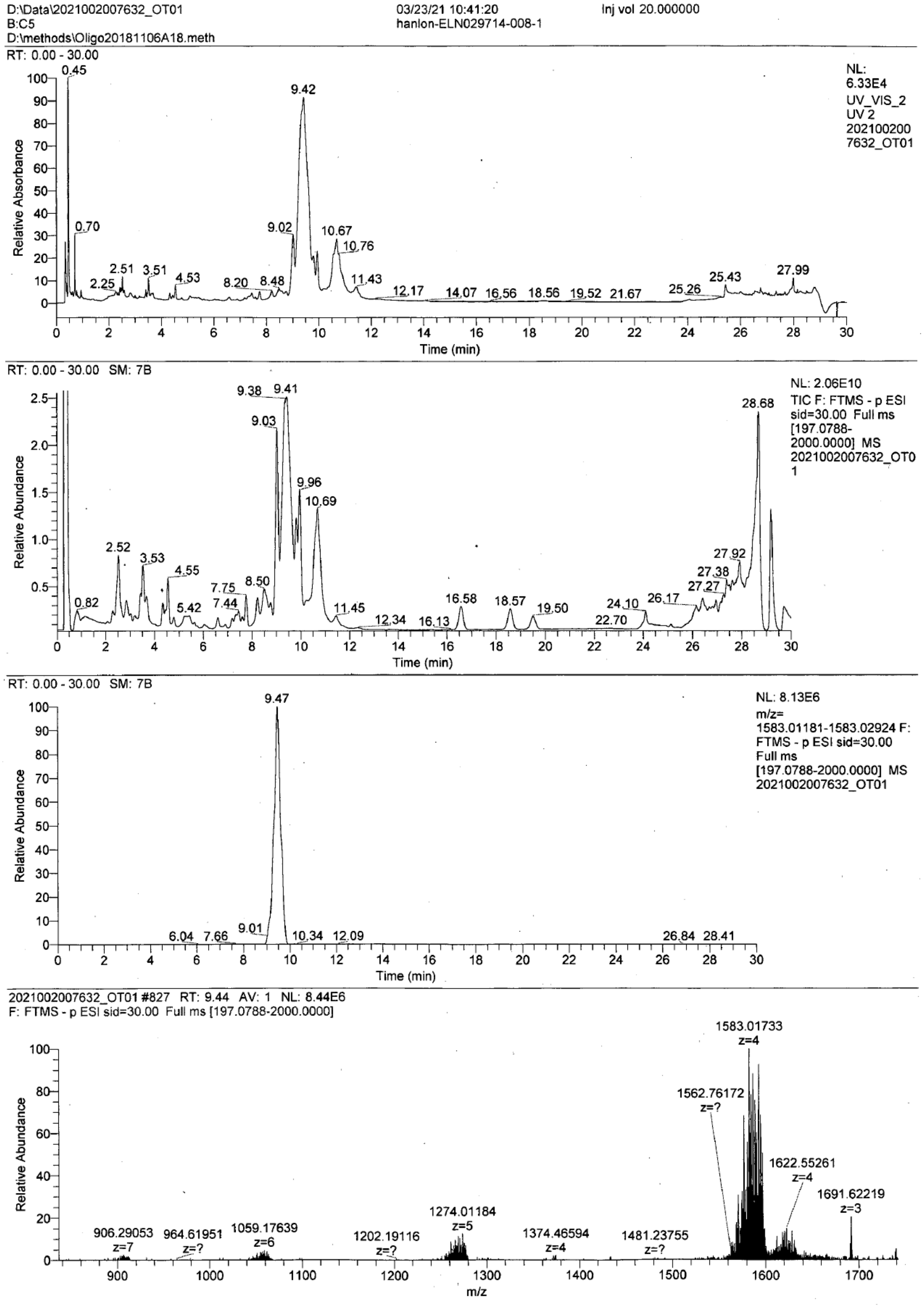

Supplementary Figure 31. LCMS profiles and ESI-MS analysis.

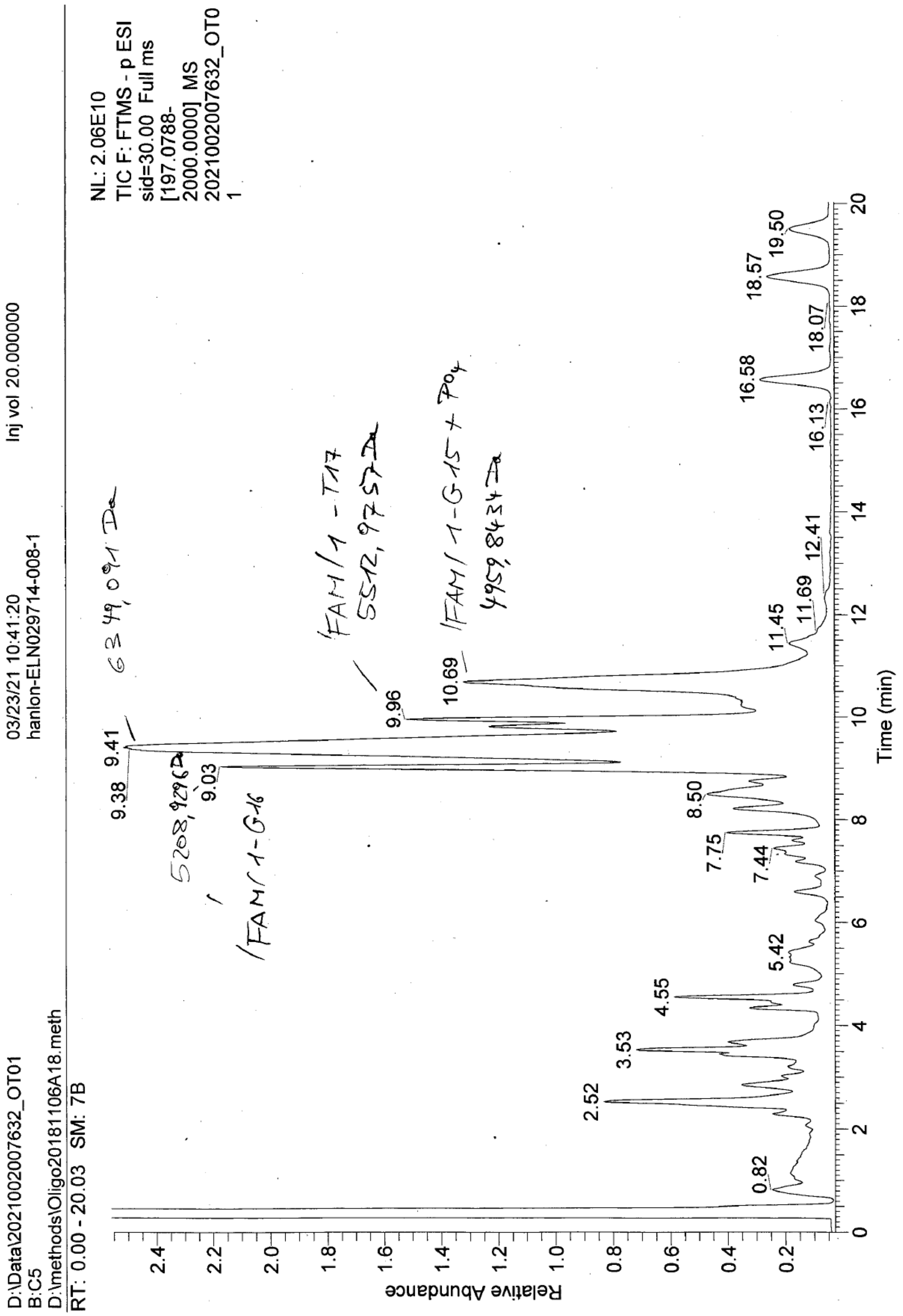

Supplementary Figure 32. Close-up view of the LCMS analysis.

RT (min)	Calculated Mass (Da)	Observed Mass (Da)	Mass Error	Intensity	Sequence
			-0.0006 Da (-0.1 ppm)		
7.456	4840.7927	4840.792	-0.0007 Da (-0.1 ppm)	2.12E+007	Nuc1:N2-C17 +3'Phos, Nuc1:N2-C17 +5'Phos
7.753	1792.2949	1792.306	0.0111 Da (6.2 ppm)	6.33E+004	Nuc1:C12-C17 +3'Phos, Nuc1:C12-C17 +5'Phos
7.753	5149.0957	5149.099	0.0033 Da (0.6 ppm)	1.26E+006	Nuc1:N3-T19 +3'Phos, Nuc1:N3-T19 +5'Phos
7.753	5706.9825	5706.976	-0.0065 Da (-1.1 ppm)	5.78E+005	Nuc1:N2-G20
8.221	4013.7057	4013.675	-0.0307 Da (-7.6 ppm)	1.54E+005	Nuc2:C2-G14
8.221	4879.8771	4879.876	-0.0011 Da (-0.2 ppm)	5.84E+007	Nuc2:/FAM/1-G15
8.496	5153.8503	5153.852	0.0017 Da (0.3 ppm)	2.24E+007	Nuc1:N2-A18 +3'Phos, Nuc1:N2-A18 +5'Phos
8.496	5478.1482	5478.143	-0.0052 Da (-0.9 ppm)	1.25E+006	Nuc1:N3-G20 +5'Phos
8.759	1767.3344	1767.329	-0.0054 Da (-3.1 ppm)	1.03E+005	Nuc1:T10-C15
9.033	5208.9296	5208.933	0.0034 Da (0.7 ppm)	3.81E+008	Nuc2:/FAM/1-G16
9.432	4301.7384	4301.737	-0.0014 Da (-0.3 ppm)	2.21E+007	Nuc2:/FAM/1-T13 +3'Phos
9.432	5073.8840	5073.894	0.0100 Da (2.0 ppm)	3.60E+005	Nuc1:N2-A18
9.432	5135.7894	5135.819	0.0296 Da (5.8 ppm)	1.02E+005	Nuc2:C2-/3Phos/18 +5'Phos
9.432	5457.8963	5457.896	-0.0003 Da (-0.1 ppm)	6.60E+006	Nuc1:N2-T19 +3'Phos, Nuc1:N2-T19 +5'Phos
9.957	4630.7909	4630.789	-0.0019 Da (-0.4 ppm)	3.19E+007	Nuc2:/FAM/1-G14 +3'Phos
9.957	5512.9757	5512.978	0.0023 Da (0.4 ppm)	1.25E+008	Nuc2:/FAM/1-T17
10.690	4959.8434	4959.845	0.0016 Da (0.3 ppm)	1.99E+007	Nuc2:/FAM/1-G15 +3'Phos
10.690	4975.8568	4975.874	0.0172 Da (3.5 ppm)	7.01E+004	Nuc2:C2-T17
10.690	5786.9488	5786.951	0.0022 Da (0.4 ppm)	5.97E+006	Nuc1:N2-G20 +5'Phos
11.445	5288.8959	5288.897		2.34E+006	Nuc2:/FAM/1-G16 +3'Phos

Supplementary Figure 33. Summary of results.

d) Characterization of PEX products stemming from the reaction obtained with the 3'-phos-dTTP **5** and Kf (*exo*⁻) on the **P1/T3** system (see Table 2 of manuscript):

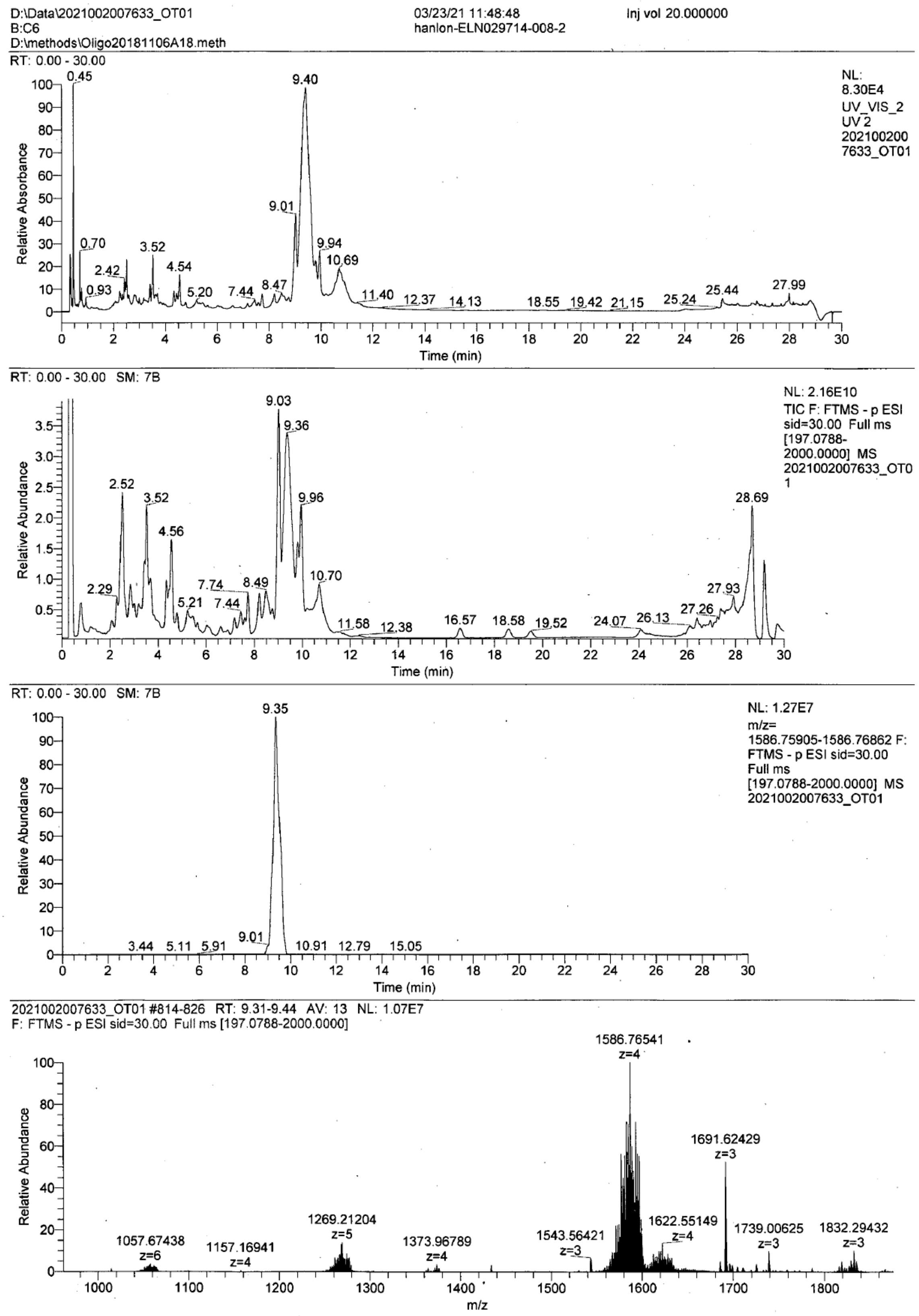

Supplementary Figure 34. LCMS profiles and ESI-MS analysis.

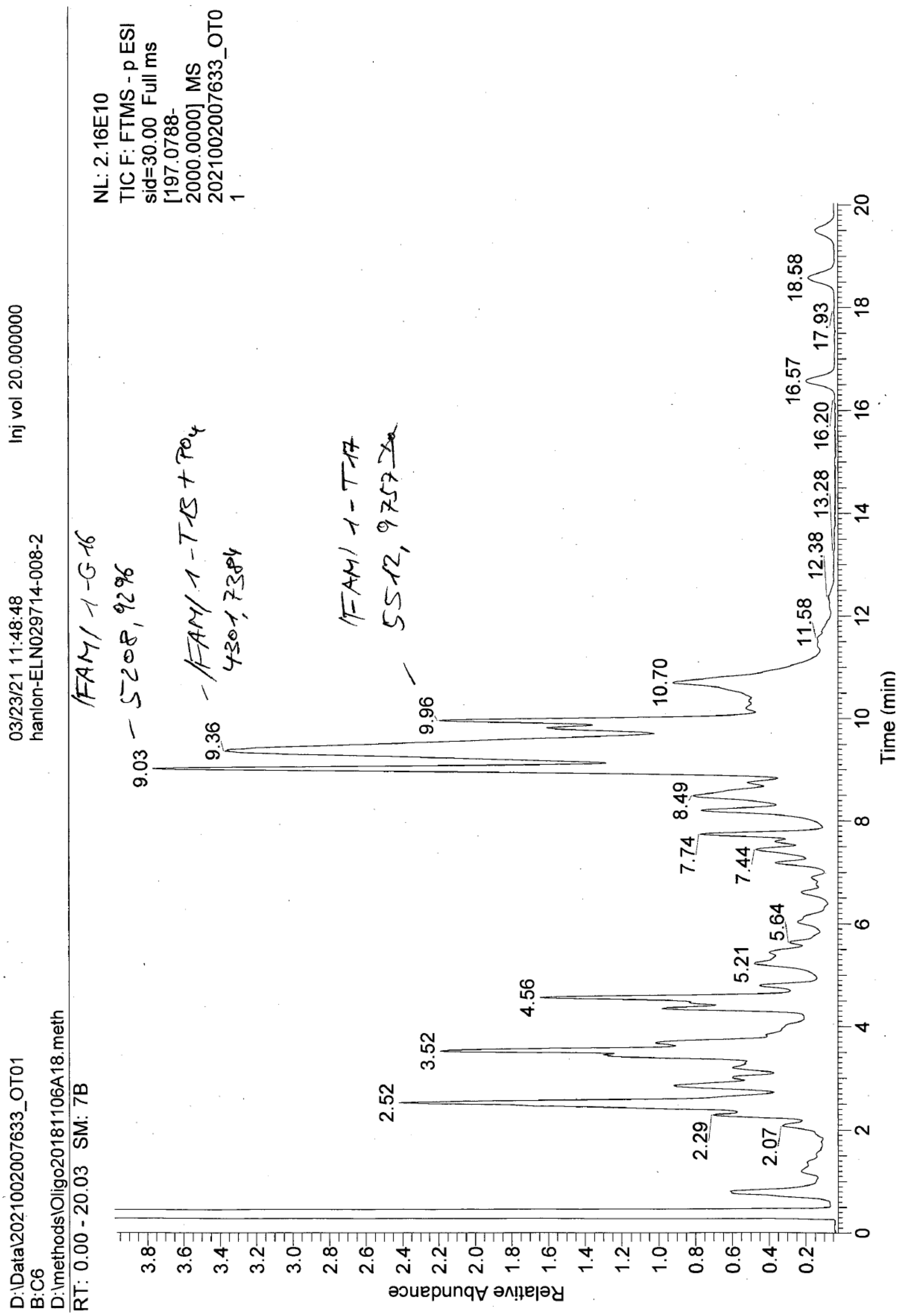

Supplementary Figure 35. Close-up view of the LCMS analysis.

RT (min)	Calculated Mass (Da)	Observed Mass (Da)	Mass Error	Intensity	Sequence
			-0.0433 Da (-9.1 ppm)		
9.031	5208.9296	5208.935	0.0054 Da (1.0 ppm)	7.85E+008	Nuc2:/FAM/1-G16
9.351	4301.7384	4301.737	-0.0014 Da (-0.3 ppm)	4.23E+007	Nuc2:/FAM/1-T13 +3'Phos
9.351	5073.8840	5073.896	0.0120 Da (2.4 ppm)	6.09E+005	Nuc1:N2-A18
9.351	5457.8963	5457.896	-0.0003 Da (-0.1 ppm)	1.04E+007	Nuc1:N2-T19 +5'Phos, Nuc1:N2-T19 +3'Phos
9.957	4630.7909	4630.790	-0.0009 Da (-0.2 ppm)	5.34E+007	Nuc2:/FAM/1-G14 +3'Phos
9.957	5512.9757	5512.979	0.0033 Da (0.6 ppm)	2.19E+008 ←	Nuc2:/FAM/1-T17 — Phos
9.957	5592.9420	5592.908	-0.0340 Da (-6.1 ppm)	5.78E+005 ←	Nuc2:/FAM/1-/3Phos/18, Nuc2:/FAM/1-T17 +3'Phos
9.957	5786.9488	5786.951	0.0022 Da (0.4 ppm)	8.30E+006	Nuc1:N2-G20 +5'Phos
10.713	4959.8434	4959.846	0.0026 Da (0.5 ppm)	2.59E+007	Nuc2:/FAM/1-G15 +3'Phos
10.713	4975.8568	4975.873	0.0162 Da (3.3 ppm)	1.35E+005	Nuc2:C2-T17
11.576	1543.2547	1543.269	0.0143 Da (9.3 ppm)	5.96E+004	Nuc1:G11-C15 +5'Phos, Nuc1:G11-C15 +3'Phos
11.576	5288.8959	5288.898	0.0021 Da (0.4 ppm)	2.21E+006	Nuc2:/FAM/1-G16 +3'Phos

Chromatogram Summary

Supplementary Figure 36. Summary of results.

e) Characterization of PEX products stemming from the reaction obtained with the 3'-phos-LNA-TTP **10** and Vent (*exo*⁻) on the **P1**/**T3** system (see Table 2 of manuscript):

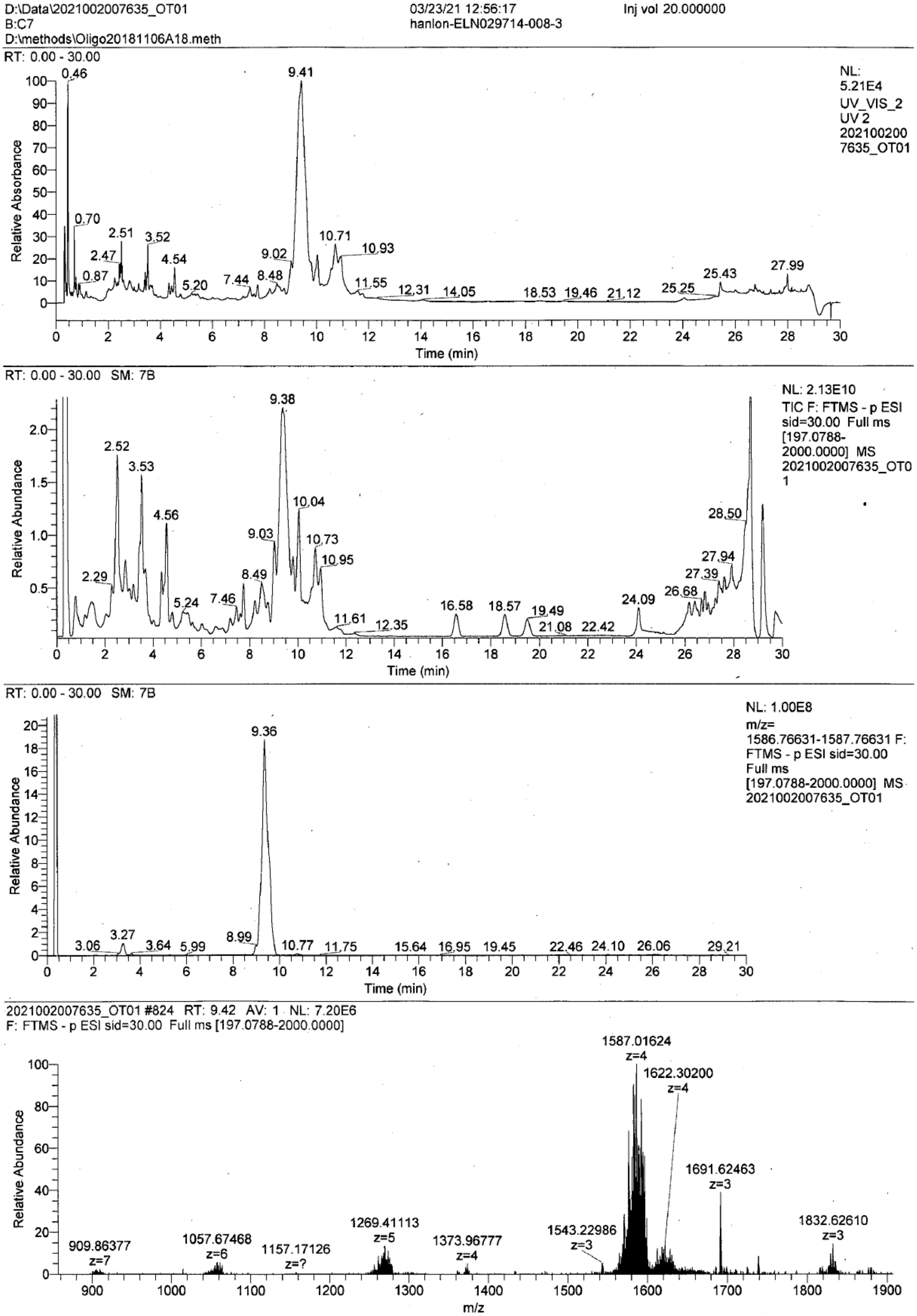

Supplementary Figure 37. LCMS profiles and ESI-MS analysis.

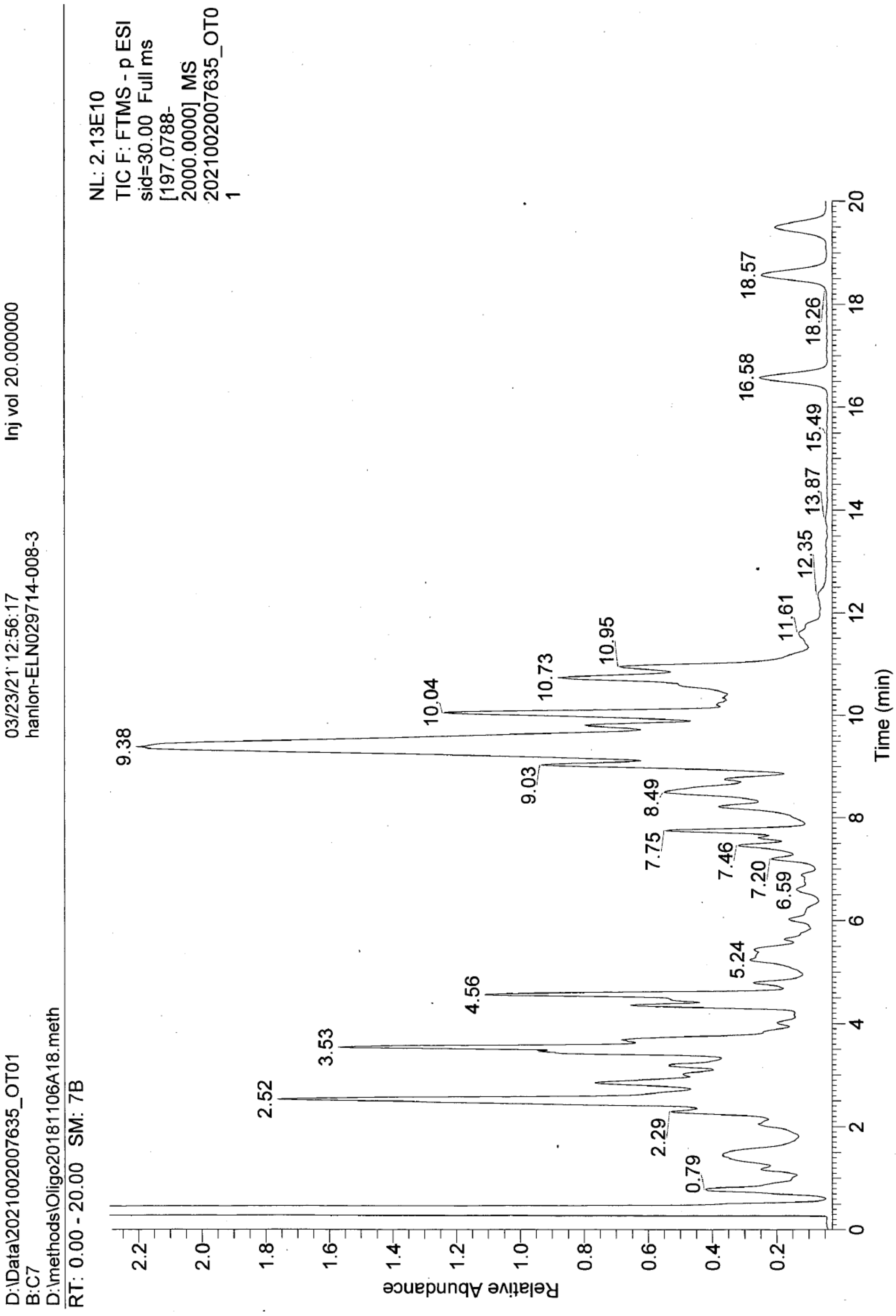

Supplementary Figure 38. Close-up view of the LCMS analysis.

Chromatogram Summary

RT (min)	Base Peak Mass (Da)	Intensity	Spectral Quality	LC/MS Peak Area	LC/MS Area Percent
0.787	1002.118	9.21E+005	ok	9.01E+008	2.71
1.175	1309.213	5.87E+006	ok	2.41E+008	0.73
1.449	1309.213	1.23E+006	ok	1.10E+009	3.31
2.020	1019.967	6.07E+006	ok	9.23E+007	0.28
2.283	1019.967	6.61E+006	ok	3.45E+008	1.04
2.534	2144.359	7.11E+007	ok	3.00E+009	9.04
2.842	2473.411	1.20E+007	ok	9.34E+008	2.81
3.186	2858.466	3.10E+007	ok	2.30E+008	0.69
3.539	3027.494	9.79E+007	ok	2.24E+009	6.75
3.699	2119.381	2.91E+007	ok	9.21E+008	2.78
4.008	2853.527	1.62E+007	ok	5.74E+007	0.17
4.351	3660.595	1.68E+008	ok	6.18E+008	1.86
4.557	3684.606	1.01E+008	ok	1.64E+009	4.95
4.797	2737.482	2.73E+007	ok	2.43E+008	0.73
5.231	3973.652	1.24E+007	ok	4.79E+008	1.44
5.448	3066.534	1.59E+007	ok	4.36E+008	1.31
6.021	3800.680	8.00E+006	ok	1.94E+008	0.58
6.614	4551.744	6.00E+006	ok	1.30E+008	0.39
6.900	3864.642	9.43E+006	ok	1.13E+008	0.34
7.197	3684.634	4.52E+007	ok	1.79E+008	0.54
7.460	4840.792	4.69E+007	ok	4.25E+008	1.28
7.746	4864.802	4.59E+007	ok	7.88E+008	2.37
8.215	4879.877	5.33E+007	ok	3.96E+008	1.19
8.490	5153.852	2.83E+007	ok	1.17E+009	3.53
8.764	5168.852	3.38E+007	ok	2.86E+008	0.86
9.039	5208.933	1.74E+008	ok	1.15E+009	3.45
9.359	6349.092	8.05E+007	ok	8.72E+009	26.29
9.804	5537.985	1.68E+008	ok	5.67E+008	1.71
10.044	5522.963	2.13E+008	ok	1.36E+009	4.10
10.731	5852.014	6.43E+007	ok	1.51E+009	4.55
10.948	5836.994	6.95E+007	ok	9.55E+008	2.88
11.614	6166.045	7.56E+006	ok	1.81E+008	0.55
16.569	1372.453	7.95E+005	ok	5.54E+008	1.67
18.580	1372.453	7.37E+005	ok	5.67E+008	1.71
19.506	1233.628	8.68E+005	ok	4.64E+008	1.40

Supplementary Figure 39. Summary of results.

f) Characterization of PEX products stemming from the reaction obtained with the 3'-phos-LNA-TTP 10 and Kf (*exo*⁻) on the **P1/T3** system (see Table 2 of manuscript):

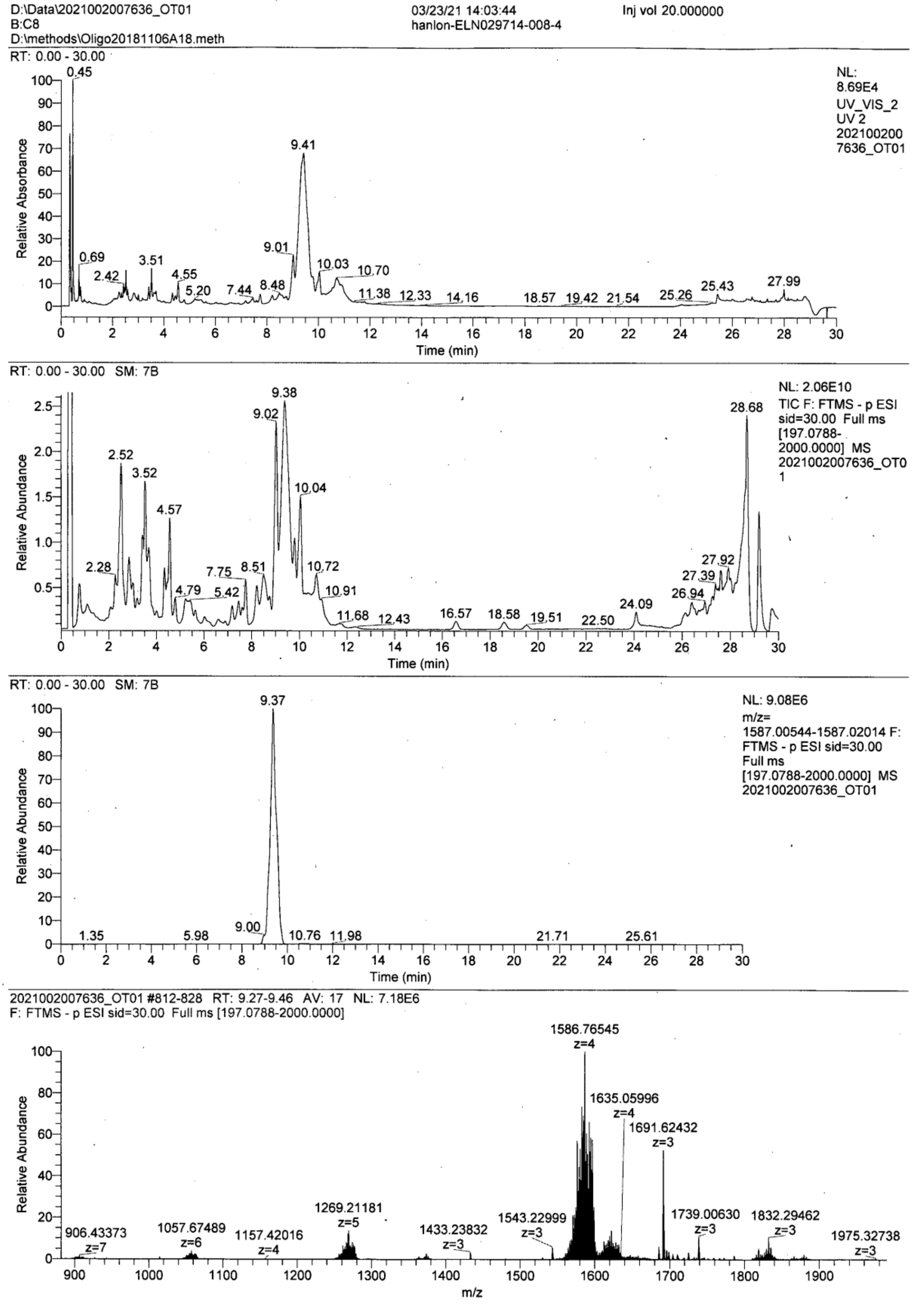

Supplementary Figure 40. LCMS profiles and ESI-MS analysis.

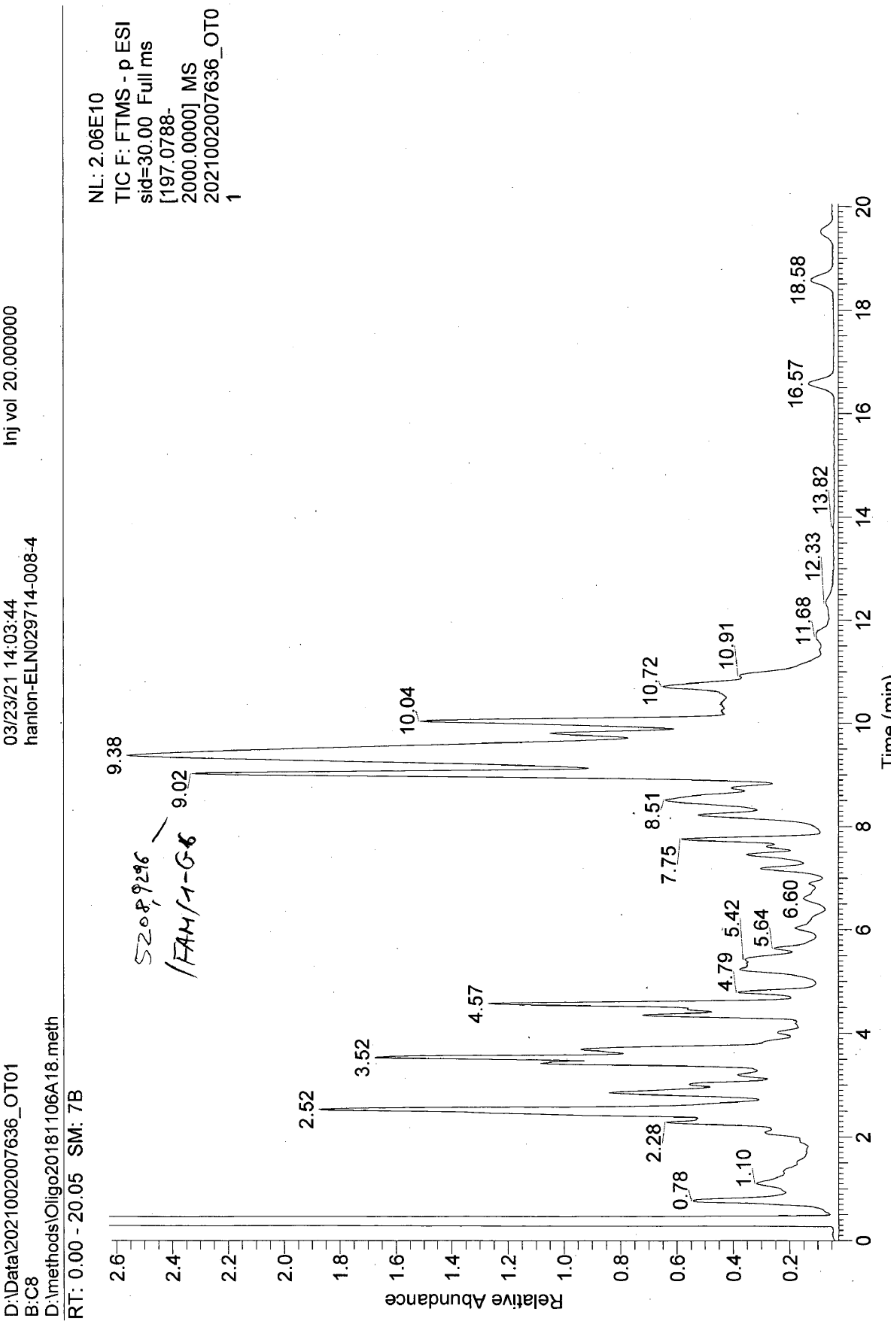

Supplementary Figure 41. Close-up view of the LCMS analysis.

RT (min)	Base Peak Mass (Da)	Intensity	Spectral Quality	LC/MS Peak Area	LC/MS Area Percent
3.185	2858.466	1.49E+007	ok	1.61E+008	0.43
3.527	3027.495	1.11E+008	ok	2.67E+009	7.10
3.698	2119.382	5.47E+007	ok	1.49E+009	3.96
4.018	2853.528	1.90E+007	ok	7.45E+007	0.20
4.349	3660.595	1.08E+008	ok	6.93E+008	1.84
4.566	3675.596	1.06E+008	ok	1.83E+009	4.87
4.795	2737.482	4.89E+007	ok	3.70E+008	0.98
5.229	3973.652	1.71E+007	ok	4.69E+008	1.25
5.423	3957.656	1.78E+007	ok	7.23E+008	1.92
5.640	3066.534	4.03E+007	ok	2.22E+008	0.59
6.028	3800.680	1.01E+007	ok	2.30E+008	0.61
6.622	4551.746	4.90E+006	ok	1.94E+008	0.51
6.897	4575.755	1.10E+007	ok	6.29E+007	0.17
7.182	3684.634	6.20E+007	ok	3.32E+008	0.88
7.456	4840.791	5.49E+007	ok	5.04E+008	1.34
7.754	4864.802	4.86E+007	ok	9.14E+008	2.43
8.211	4879.876	7.94E+007	ok	6.42E+008	1.71
8.497	5153.852	2.65E+007	ok	1.58E+009	4.20
9.023	5208.933	4.41E+008	ok	3.40E+009	9.02
9.366	6349.091	8.80E+007	ok	9.35E+009	24.84
9.801	5537.984	2.34E+008	ok	6.88E+008	1.83
10.053	5522.963	2.34E+008	ok	1.68E+009	4.45
10.705	4959.846	2.27E+007	ok	1.55E+009	4.11
11.763	6151.020	4.03E+006	ok	8.48E+007	0.23
16.582	1237.660	2.85E+005	ok	2.04E+008	0.54
18.576	1237.660	2.52E+005	ok	2.03E+008	0.54
19.517	1233.628	2.18E+005	ok	1.05E+008	0.28

Supplementary Figure 42. Summary of results.

g) LCMS analysis of reaction products obtained with the TdT, primer **P2**, Co^{2+}, and 3h of reaction at 37°C

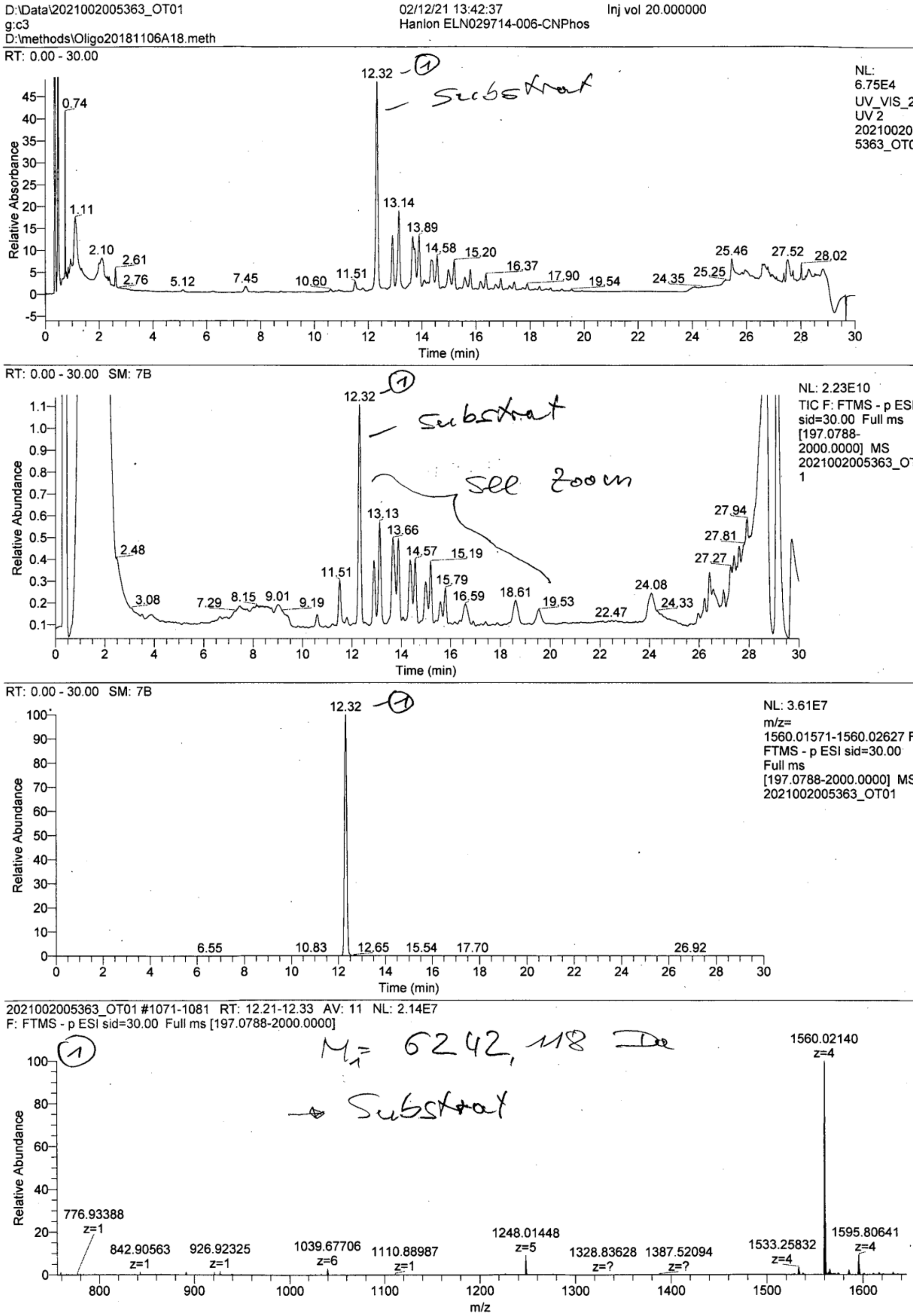

Supplementary Figure 43. LCMS profiles and ESI-MS analysis.

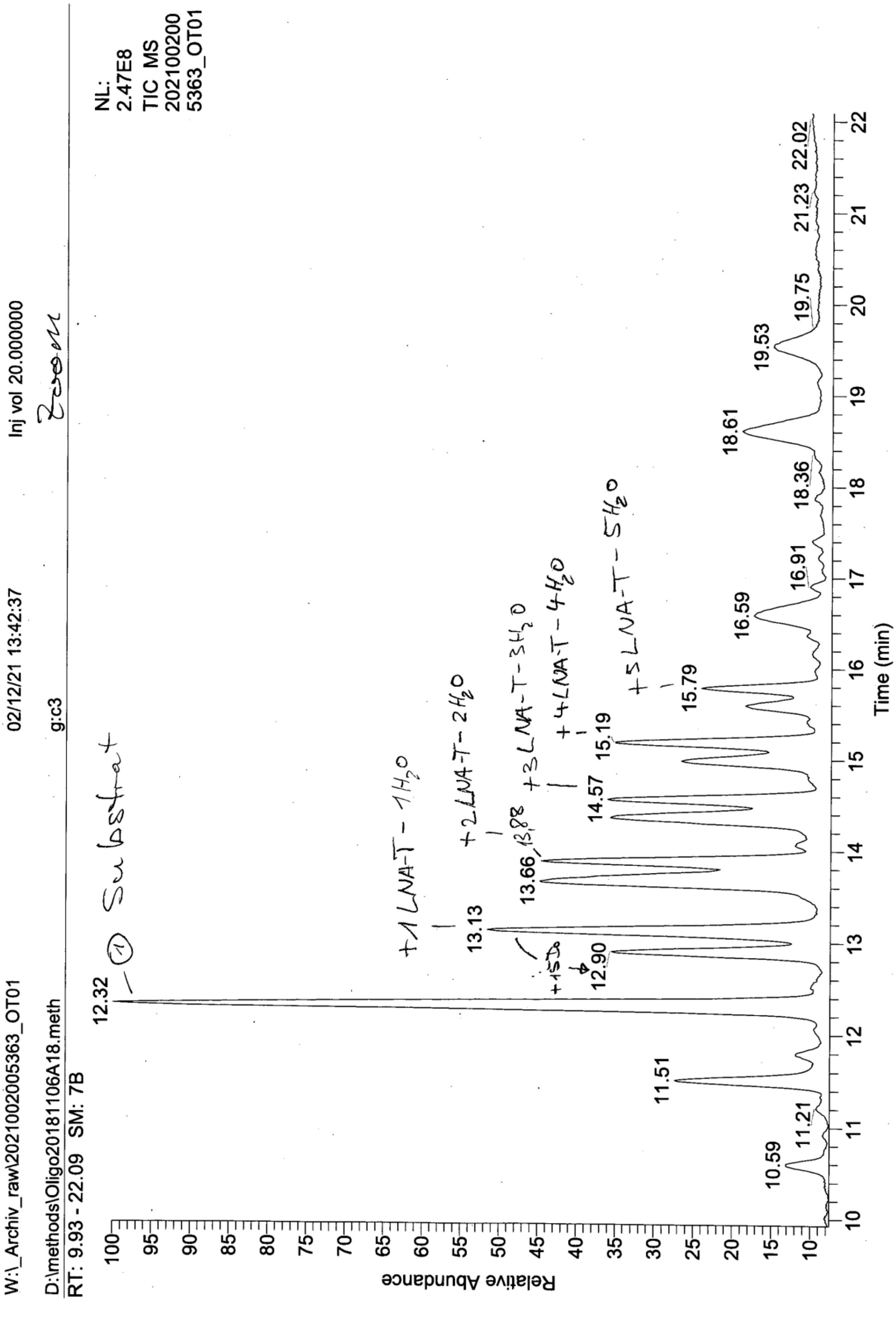

Supplementary Figure 44. Close-up view of the LCMS analysis.

RT (min)	Base Peak Mass (Da)	Difference (Da)	LC/MS Area Percent	Proposal
10.59	4'508.7790	-1'733.3390	1.1	
11.21	4'855.8420	-1'386.2760	0.2	
11.51	4'821.8380	-1'420.2800	4.2	
11.79	5'953.0620	-289.0560	0.6	
12.32	6'242.1180	0.0000	19.7	Substrate
12.90	6'571.1710	329.0530	5.4	+ 1LNA-T - 1H_2O + 15 Da
13.13	6'556.1480	314.0300	9.9	+ 1LNA-T - 1H_2O
13.65	6'885.2000	643.0820	11.4	+ 2LNA-T - 2H_2O + 15 Da
13.88	6'870.1770	628.0590	7.7	+ 2LNA-T - 2H_2O
14.37	7'199.2290	957.1110	8.2	+ 3LNA-T - 3H_2O + 15 Da
14.57	7'184.2070	942.0890	5.7	+ 3LNA-T - 3H_2O
14.99	7'513.2590	1'271.1410	4.5	+ 4LNA-T - 4H_2O + 15 Da
15.19	7'498.2390	1'256.1210	5.5	+ 4LNA-T - 4H_2O
15.42	737.9490	-5'504.1690	0.1	
15.59	7'827.2870	1'585.1690	2.2	+ 5LNA-T - 5H_2O + 15 Da
15.79	7'812.2670	1'570.1490	2.9	+ 5LNA-T - 5H_2O
16.59	640.1400	-5'601.9780	3.8	
16.91	8'440.3220	2'198.2040	0.3	
17.41	8'754.3550	2'512.2370	0.2	
17.89	9'068.3870	2'826.2690	0.1	
18.61	640.1400	-5'601.9780	4.3	
19.53	638.1240	-5'603.9940	2.3	

Supplementary Figure 45. Summary of results.

3. Oligonucleotides used for enzymatic synthesis:

Primer **P1**: 5‘-FAM-CAT GGG CGG CAT GGG-3’

Template **T1**: 5‘-CTA GCA TGA GCT CAG TCC CAT GCC GCC CAT G-3’

Template **T2**: 5‘-AAA AAA ACC CAT GCC GCC CAT G-3’

Template **T3**: 5‘-NNN NNC CCA TGC CGC CCA TG-3’

Primer **P2**: 5‘-FAM-TAC GAC TCA CTA TAG CCT C-3’

All templates and primers were purchased HPLC-purified from Integrated DNA Technologies (IDT).

4. Synthesis of nucleosides and nucleotides

Materials and methods

All reactions were performed under argon in flame-dried glassware. Anhydrous solvents for reactions were obtained from Sigma Aldrich. Flash chromatography was performed on a Reveleris Prep system from Büchi. Thin layer chromatography was carried out on pre-coated glass-backed plates of silica gel (0.25 mm, UV_{254}) from Macherey-Nagel. All chemicals and solvents used were purchased from Sigma-Aldrich and Alfa Aesar unless stated otherwise.

NMR spectra were recorded on a Bruker Avance 400 spectrometer (400.1 MHz for ^{1}H, 100.6 MHz for ^{13}C, and 161.6 MHz for ^{31}P) or on an Agilent DirectDrive 500 MHz spectrometer (Agilent Technologies, Santa Clara) with a proton resonating frequency of 499.8 MHz (125.7 MHz for ^{13}C and 201.9 for ^{31}P). All spectra were referenced to the signals of the corresponding solvent. Chemical shifts are given in ppm (δ scale) and coupling constants (*J*) in Hz. Assignation of the NMR signals was performed by using a combination of $^{1}H/^{1}H$-COSY, ^{13}C-DEPT-135, and $^{13}C/^{1}H$-HMBC experiments.

Electrospray ionization mass spectrometry (ESI-MS): Experiments were carried out using an LTQ-Orbitrap XL from Thermo Scientific (Thermo Fisher Scientific, Courtaboeuf, France) and operated in positive ionization mode, with a spray voltage of 3.6 kV. No sheath and auxiliary gay were used. Applied voltages were 40 and 100 V for the ion transfer capillary and the tube lens, respectively. The ion transfer capillary was held at 275°C. Detection was achieved in the Orbitrap with a resolution set to 100.000 (at m/z 400) and a m/z range between 150–2000 in profile mode. Spectrum was analyzed using the acquisition software XCalibur 2.1 (Thermo Fisher Scientific, Courtaboeuf, France). The automatic gain control (AGC) allowed the accumulation of up to 21×10^{5} ions for FTMS scans, maximum injection time was set to 300 ms and 1 µscan was acquired. 10 µl was injecting using a Thermo Finnigan Surveyor HPLC system (Thermo Fisher Scientific, Courtaboeuf, France) with a continuous infusion of methanol (MeOH) at 100 $\mu l min^{-1}$.

HPLC purification was performed using an Äkta™ pure system (GE Healthcare) equipped with Thermo Scientific™ DNAPac™ PA100 semi-preparative ion exchange column. All the DNA polymerases (Phusion, Hemo KlenTaq, Taq, *Bst*, Q5, Therminator, Vent (*exo*$^{-}$), Dpo4, Deep Vent, Phi29, and Kf *exo*$^{-}$) were purchased from New England Biolabs as well as the natural dNTPs. Acrylamide/bisacrylamide (29:1, 40%) was obtained from Fisher Scientific. Visualization of PAGE gels was performed by fluorescence imaging using a using a Storm 860 or a Typhoon Trio phosphorimager with the ImageQuant software (both from GE Healthcare).

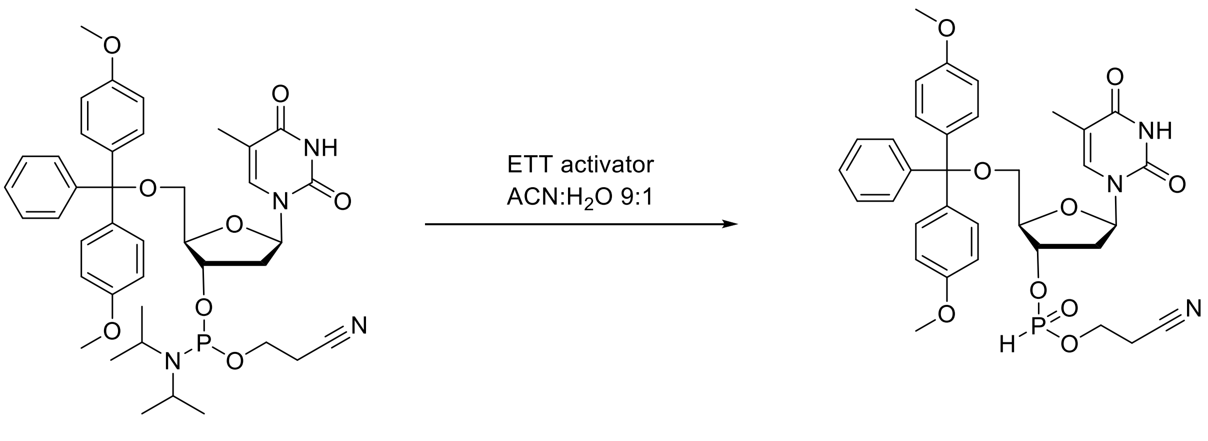

1 **2**

Commercially available phosphoramidite **1** (40 mg, 0.054 mmoles, 1 eq) was dissolved in acetonitrile (0.9 mL). 0.4 mL of a solution of 0.25 M 5-ethylthiotetrazole in acetonitrile was added (0.108 mmoles, 2 eq) with 0.1 mL of water. The mixture was stirred at room temperature for 10 min. The mixture was then diluted with $CHCl_3$. The $CHCl_3$ layer was washed three times with a solution of sat. $NaHCO_3$, dried over $MgSO_4$ and concentrated under reduced pressure. Nucleotide **2** was used without further purification.

^{31}P NMR (161.62 MHz, $CDCl_3$) δ 3.88 (s, 1P), 3.90 (s, 1P). ESI-MS: $[M+Na]^+$: calcd: 684.2081 ; found: 684.2713.

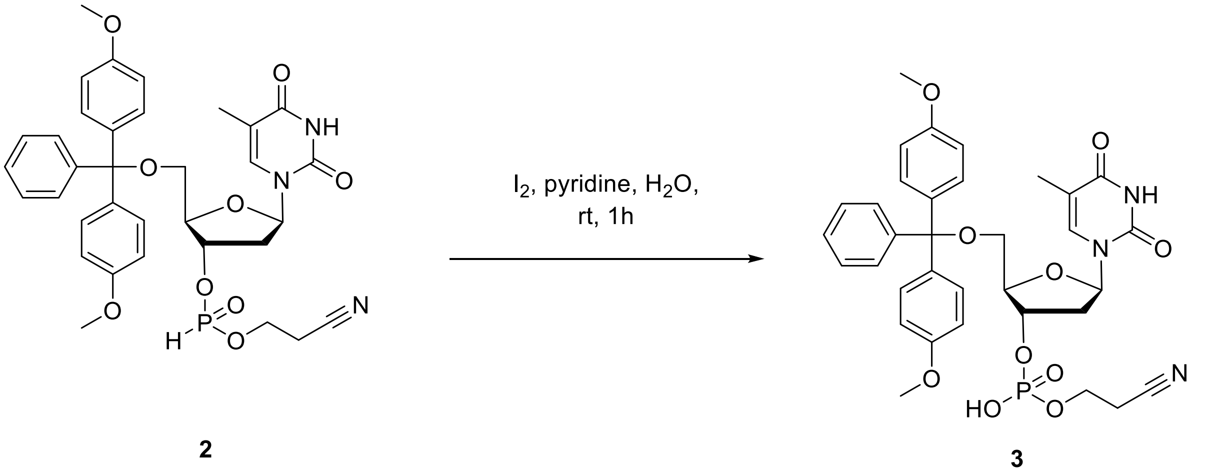

2 **3**

To a solution of *H*-phosphonate **2** (35 mg, 0.053 mmoles, 1 eq) in pyridine/H_2O (9 :1 v/v, 3 mL) was added 34 mg of iodine (0.133 mmoles, 2.5 eq). The mixture was stirred at room temperature for 5 min. It was diluted with $CHCl_3$ and a solution of $Na_2S_2O_3$ in water was added. The $CHCl_3$ layer was washed three times with a solution of sat. $NaHCO_3$, dried over $MgSO_4$ and concentrated under reduced pressure. Nucleotide **3** was used without further purification.

^{31}P NMR (161.62 MHz, $CDCl_3$) δ -4.44 (s, 1P).

HRMS (ESI) for $[C_{34}H_{35}N_3O_{10}P]^-$ m/z $[M-H]^-$: calcd: 676.2066 ; found: 676.2062.

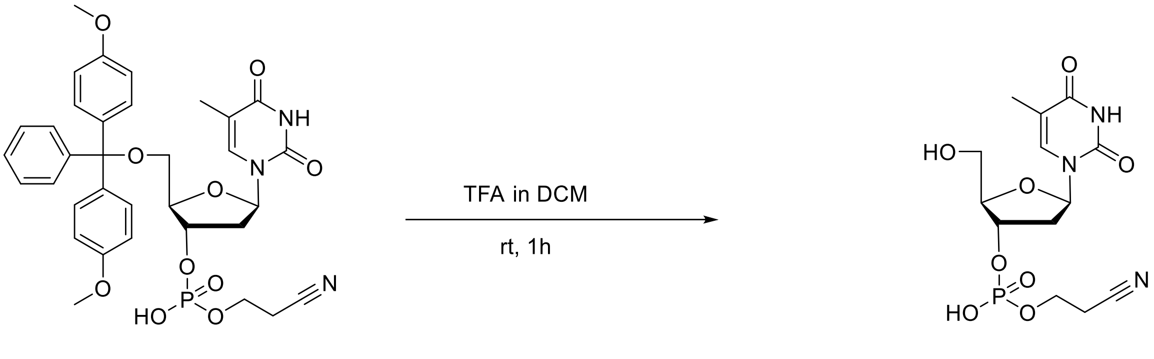

3 **4**

To a stirred solution of nucleotide **3** (150 mg, 0.221 mmol, 1 eq) in anhydrous DCM (3 mL) under Argon was added TCA (0.3 mL). The reaction mixture was stirred for 30 min at room temperature. The solvent was removed in vacuo. The residue purified by RP-HPLC (TEAA 20 mM/ MeCN/TEAA 20 mM 70:30) (70 mg, 84%). R_T = 19', 60%. Yield: 84%.

^{1}H NMR (500.13 MHz, MeOD) δ 1.87 (s, 3H), 2.27-2.50 (m, 2H), 2.80 (t, *J* = 6.50 Hz, 2H), 3.80 (d, *J* = 3.50 Hz, 2H), 4.04 (q, *J* = 6.00, 7.00 Hz, 2H), 4.14-4.15 (m, 1H), 4.83-4.86 (m, 1H), 6.30 (t, *J* = 5.50 Hz, 1H), 7.84 (s, 1H).

^{13}C NMR (100.62 MHz, MeOD) δ 13.7, 21.6, 41.3, 62.9, 64.1, 78.2, 87.3, 89.1, 112.8, 120.6, 139.4, 153.6, 167.6.

^{31}P NMR (161.62 MHz, MeOD) δ -4.46 (s, 1P).

HRMS (ESI) for $C_{13}H_{17}N_3O_8P^-$ m/z calcd: 374.0759; found: 374.0744.

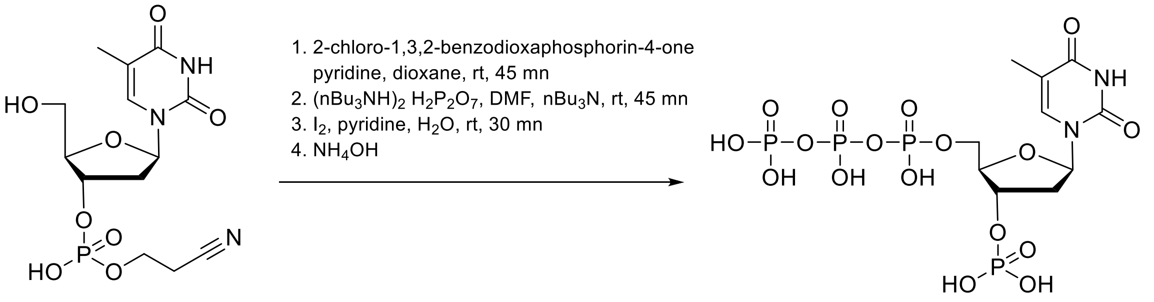

4 **5**

Starting material was coevaporated twice with pyridine and dried under reduced pressure overnight before the reaction. Tributylammonium pyrophosphate was dried under reduced pressure overnight before the reaction.

Nucleotide **4** (70 mg, 0.187 mmol, 1 eq) was dissolved in dry pyridine (0.3 mL) and dried dioxane (0.4 mL) at room temperature under inert atmosphere. To this solution, 2-chloro-1,3,2-benzodioxaphosphorin-4-one (54 mg, 0.261, 1.4 eq) was added and the reaction mixture was stirred for 45 min. A solution of tributylammonium pyrophosphate (133 mg, 0.243 mmol, 1.3 eq), in dry DMF (0.3 mL) and tributylamine (0.15 mL) was added dropwise and the reaction mixture was stirred for another 45 min. It was then oxidized by the addition of iodine (76 mg, 0.299 mmol, 1.6 eq) in pyridine (1 mL) and H_2O (0.3 mL). After 30 min of stirring, the excess of iodine was quenched with a sodium thiosulfate solution (10% w/v in water) and the solution

was concentrated under reduced pressure at 30°C. The residue was treated with aqueous ammonia and methylamine (7 mL, 1:1) for 2h. The suspension was then concentrated under reduced pressure at 30°C. The residue was dissolved in H_2O and precipitated by the addition of $NaClO_4$ 2% in acetone. The crude product was purified by HPLC. Buffer A: 10 mM TEAB, Buffer B: 1mM TEAB. R_T = 18', 46%. 13 mg of desired product were isolated (12% yield).

^{1}H NMR (400.13 MHz, D_2O) δ 1.79 (s, 3H), 2.25-2.40 (m, 2H), 4.09 (s, 2H), 4.26 (s, 1H), 4.80 (t, *J* = 7.00 Hz, 1H), 6.25 (t, *J* = 5.50 Hz, 1H), 7.64 (s, 1H).

^{31}P NMR (161.62 MHz, D_2O) δ -25.5 (t, 1H), -14.04 (bs, 2H), -3.65 (d, 1H).

HRMS (ESI) for $C_{10}H_{17}N_2O_{17}P_4^-$ m/z calcd: 560.9483; found: 560.9483.

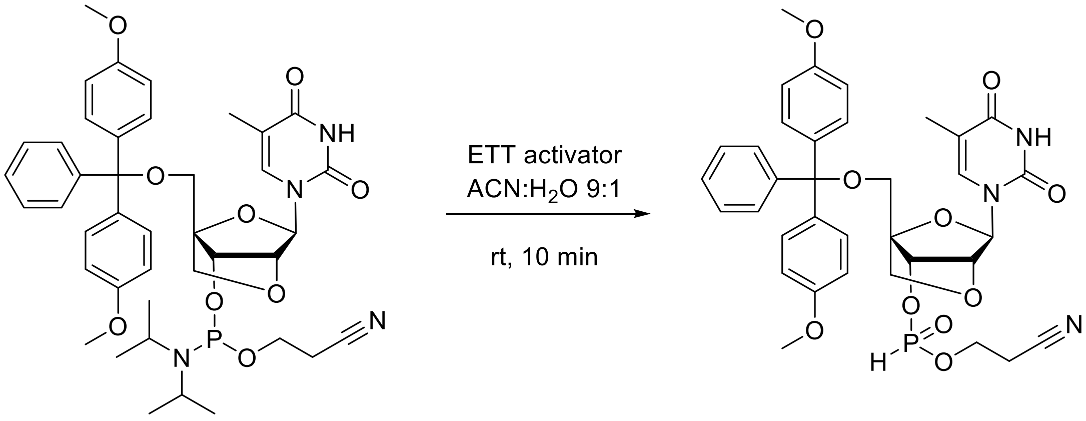

6 **7**

LNA phosphoramidite **6** (150 mg, 0.194 mmoles, 1 eq) was dissolved in acetonitrile (2 mL). 1.2 mL of a solution of 0.25 M 5-ethylthiotetrazole in acetonitrile was added (0.388 mmoles, 2 eq) with 0.2 mL of water. The mixture was stirred at room temperature for 10 min. The mixture was then diluted with $CHCl_3$. The $CHCl_3$ layer was washed three times with a solution of sat. $NaHCO_3$, dried over $MgSO_4$ and concentrated under reduced pressure. Nucleotide **7** was used without further purification.

^{31}P NMR (161.62 MHz, $CDCl_3$) δ 4.18 (s, 1P), 4.67 (s, 1P).

HRMS (ESI): $[C_{35}H_{36}N_3O_{10}P+Na]^+$: calcd: 712.2031; found: 712.2021.

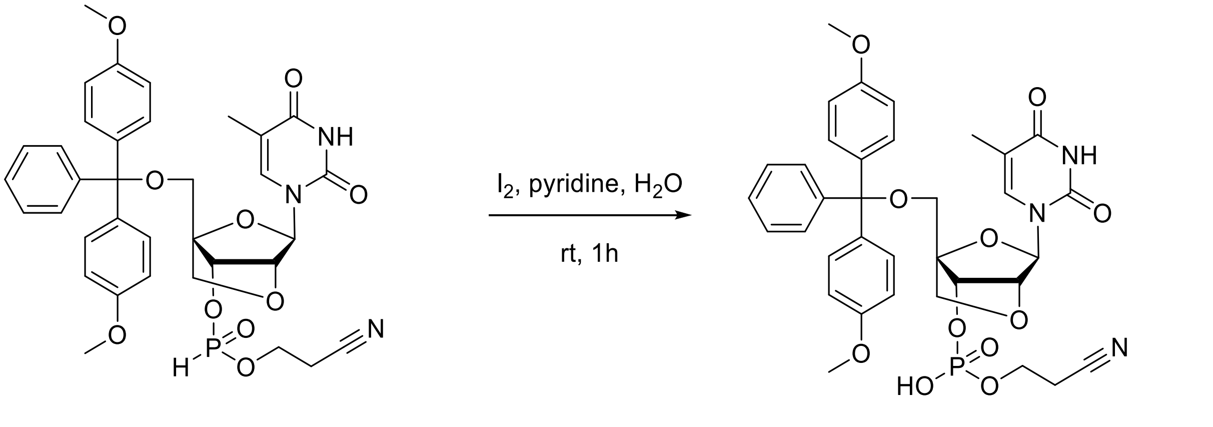

7 **8**

To a solution of *H*-phosphonate **7** (135 mg, 0.196 mmoles, 1 eq) in pyridine/H_2O (9 :1 v/v, 7 mL) was added 124 mg of iodine (0.490 mmoles, 2.5 eq). The mixture was stirred at room temperature for 5 min. It was diluted with $CHCl_3$ and a solution of $Na_2S_2O_3$ in water was added. The $CHCl_3$ layer was washed three times with a solution of sat. $NaHCO_3$, dried over $MgSO_4$ and concentrated under reduced pressure. Nucleotide **8** was used without further purification.

^{1}H NMR (500.13 MHz, $CDCl_3$) δ 1.64 (s, 3H), 2.59 (s, 2H), 3.64 (s, 2H), 3.72 (bs, 8H), 3.84 (bs, 2H), 4.25 (s, 1H), 4.51 (s, 1H), 5.53 (s, 1H), 6.79 (bs, 4H), 7.17-7.58 (m, 9H), 8.58 (s, 1H).

^{31}P NMR (161.62 MHz, $CDCl_3$) δ -4.62.

HRMS (ESI) for $[C_{35}H_{35}N_3O_{11}P]^-$ m/z $[M-H]^-$: calcd: 704.2015; found: 704.2019.

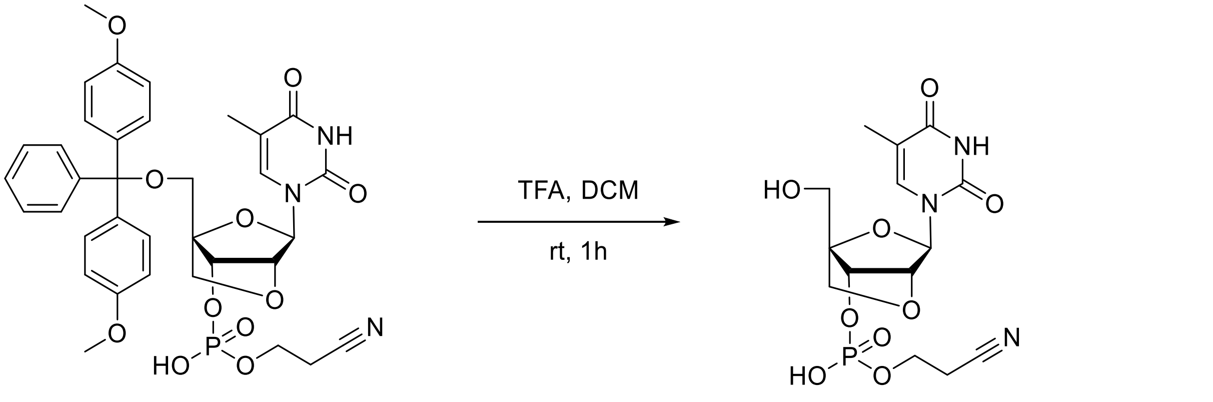

8 **9**

To a stirred solution of nucleotide **8** (150 mg, 0.221 mmol, 1 eq) in anhydrous DCM (3 mL) under Argon was added TCA (0.3 mL). The reaction mixture was stirred for 30 min at room temperature. The solvent was removed in vacuo. The crude was dissolved in water and washed with DCM. 75 mg of nucleotide **9** were isolated (yield of 97%).

^{1}H NMR (500.13 MHz, MeOD) δ 1.88 (s, 3H), 2.01 (t, *J* = 6.00 Hz, 2H), 3.82 (d, *J* = 8.00 Hz, 1H), 3.92-4.03 (, 4H), 4.05-4.09 (m, 2H), 4.47 (d, *J* = 6.00 Hz, 1H), 5.59 (s, 1H), 7.72 (s, 1H).

^{13}C NMR (100.62 MHz, MeOD) δ 13.9, 21.4, 21.5, 58.8, 63.4, 74.0, 74.7, 80.9, 81.9, 89.6, 112.4, 120.7, 137.8 ,153.3, 167.7.

^{31}P NMR (161.62 MHz, MeOD) δ -4.59.

HRMS (ESI) for $C_{14}H_{17}N_3O_9P^-$ m/z calcd: 402.0708; found: 402.0704.

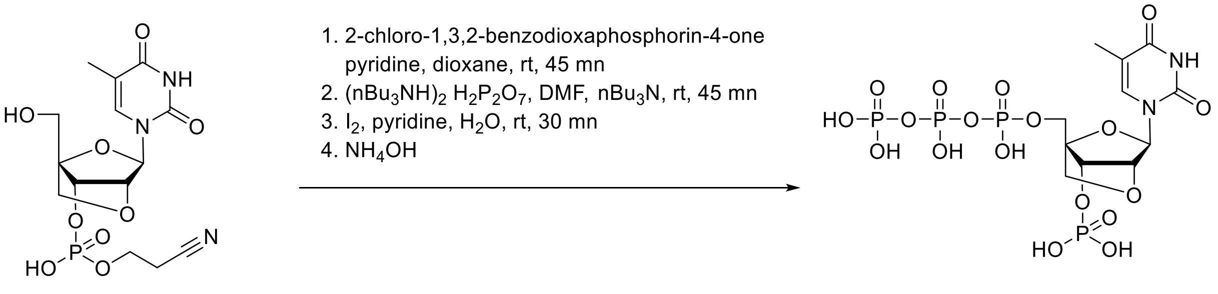

9 10

Starting material was coevaporated twice with pyridine and dried under reduced pressure overnight before the reaction. Tributylammonium pyrophosphate was dried under reduced pressure overnight before the reaction. Nucleotide **9** (75 mg, 0.186 mmol, 1 eq) was dissolved in dry pyridine (0.3 mL) and dried dioxane (0.4 mL) at room temperature under inert atmosphere. To this solution, 2-chloro-1,3,2-benzodioxaphosphorin-4-one (54 mg, 0.260, 1.4 eq) was added and the reaction mixture was stirred for 45 min. A solution of tributylammonium pyrophosphate (132 mg, 0.242 mmol, 1.3 eq), in dry DMF (0.3 mL) and tributylamine (0.15 mL) was added dropwise and the reaction mixture was stirred for another 45 min. It was then oxidized by the addition of iodine (75 mg, 0.297 mmol, 1.6 eq) in pyridine (1 mL) and H_2O (0.3 mL). After 30 min of stirring, the excess of iodine was quenched with a sodium thiosulfate solution (10% w/v in water) and the solution was concentrated under reduced pressure at 30°C. The residue was treated with aqueous ammonia and methylamine (7 mL, 1:1) for 2h. The suspension was then concentrated under reduced pressure at 30°C. The residue was dissolved in H_2O and precipitated by the addition of $NaClO_4$ 2% in acetone. The crude product was purified by HPLC. Buffer A: 10 mM TEAB, Buffer B: 1mM TEAB. R_T = 19', 47%. 26 mg of nucleotide **10** were isolated (yield of 12%).

^{1}H NMR (400.13 MHz, D_2O) δ 1.80 (s, 3H), 2.83-2.96 (m, 4H), 3.93 (s, 1H), 4.01 (s, 1H), 5.59 (s, 1H), 7.63 (s, 1H).

^{31}P NMR (161.62 MHz, D_2O) δ -26.53 (t, 1P), -14.65 (bs, 1P), -13.88 (d, 1P), -3.74 (d, 1P).

HRMS (ESI) for $C_{11}H_{17}N_2O_{18}P_4^-$ m/z calcd: 588.9432; found: 588.9435.

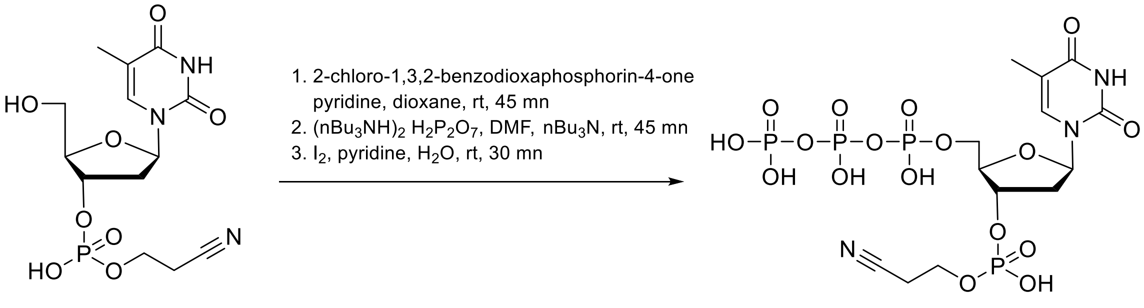

4 11

Starting material was coevaporated twice with pyridine and dried under reduced pressure overnight before the reaction. Tributylammonium pyrophosphate was dried under reduced pressure overnight before the reaction. Nucleotide **4** (75 mg, 0.187 mmol, 1 eq) was dissolved

in dry pyridine (0.5 mL) and dried dioxane (0.6 mL) at room temperature under inert atmosphere. To this solution, 2-chloro-1,3,2-benzodioxaphosphorin-4-one (55 mg, 0.262, 1.4 eq) was added and the reaction mixture was stirred for 45 min. A solution of tributylammonium pyrophosphate (134 mg, 0.243 mmol, 1.3 eq), in dry DMF (0.5 mL) and tributylamine (0.3 mL) was added dropwise and the reaction mixture was stirred for another 45 min. It was then oxidized by the addition of iodine (75 mg, 0.299 mmol, 1.6 eq) in pyridine (1 mL) and H_2O (0.4 mL). After 30 min of stirring, the excess of iodine was quenched with a sodium thiosulfate solution (10% w/v in water) and the solution was concentrated under reduced pressure at 30°C. The residue was dissolved in H_2O and precipitated by the addition of $NaClO_4$ 2% in acetone. The crude product was purified by HPLC. Buffer A: 10 mM TEAB, Buffer B: 1mM TEAB. R_T = 20', 48%. 18 mg of nucleotide **11** were isolated (yield of 16%).

^{1}H NMR (400.13 MHz, D_2O) δ 1.81 (s, 3H), 2.25-2.44 (m, 2H), 2.74-2.76 (m, 2H), 3.96-4.00 (m, 2H), 4.08-4.10 (m, 2H), 4.28 (bs, 1H), 4.80 (t, J = 5.50 Hz, 1H), 6.27 (t, J = 5.50 Hz, 1H), 7.66 (s, 1H).

^{31}P NMR (161.62 MHz, D_2O) δ -26.18 (t, J = 15.60 Hz, 1P), -14.75 (d, J = 15.80 Hz, 1P), -12.86 (bs, 1P), -4.42 (s, 1P).

HRMS (ESI) for $C_{13}H_{20}N_3O_{17}P_4^-$ m/z calcd: 613.9749; found: 613.9737.

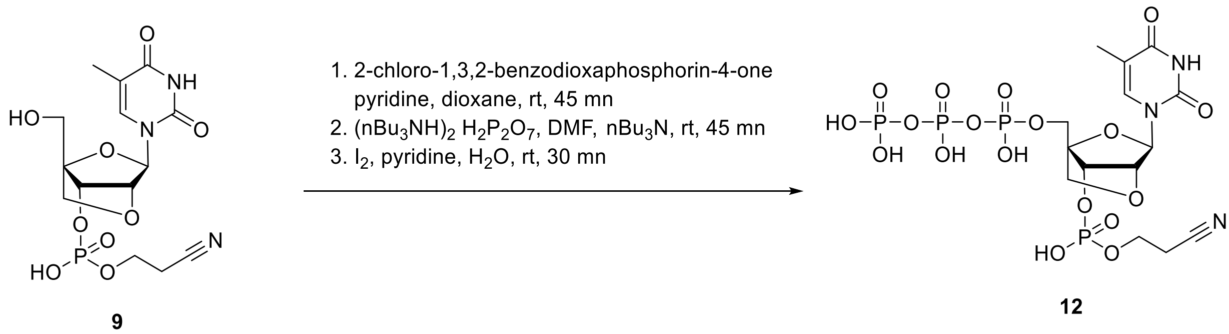

9 12

Starting material was coevaporated twice with pyridine and dried under reduced pressure overnight before the reaction. Tributylammonium pyrophosphate was dried under reduced pressure overnight before the reaction. Nucleotide **9** (77 mg, 0.191 mmol, 1 eq) was dissolved in dry pyridine (0.4 mL) and dried dioxane (0.5 mL) at room temperature under inert atmosphere. To this solution, 2-chloro-1,3,2-benzodioxaphosphorin-4-one (56 mg, 0.267, 1.4 eq) was added and the reaction mixture was stirred for 45 min. A solution of tributylammonium pyrophosphate (136 mg, 0.248 mmol, 1.3 eq), in dry DMF (0.4 mL) and tributylamine (0.2 mL) was added dropwise and the reaction mixture was stirred for another 45 min. It was then oxidized by the addition of iodine (78 mg, 0.306 mmol, 1.6 eq) in pyridine (1 mL) and H_2O (0.3 mL). After 30 min of stirring, the excess of iodine was quenched with a sodium thiosulfate solution (10% w/v in water) and the solution was concentrated under reduced pressure at 30°C. The residue was dissolved in H_2O and precipitated by the addition of $NaClO_4$ 2% in acetone. The crude product was purified by HPLC. Buffer A: 10 mM TEAB, Buffer B: 1mM TEAB. R_T = 20', 48%. 16 mg of nucleotide **12** were isolated (yield of 13%).

^{1}H NMR (400.13 MHz, D_2O) δ 1.78 (s, 3H), 2.70-2.73 (m, 2H), 2.89-2.94 (m, 2H), 3.93-3.96 (m, 2H), 4.01-4.03 (m, 1H), 4.29-4.38 (m, 2H), 4.54 (s, 1H), 5.58 (s, 1H), 7.57 (s, 1H).

^{31}P NMR (161.62 MHz, D_2O) δ -25.55 (t, 1P), -14.65 (d, 1P), -9.42 (d, 1P), -4.99 (s, 1P).

HRMS (ESI) for $C_{14}H_{20}N_3O_{18}P_4^-$ m/z calcd: 641.9698; found: 641.9696.

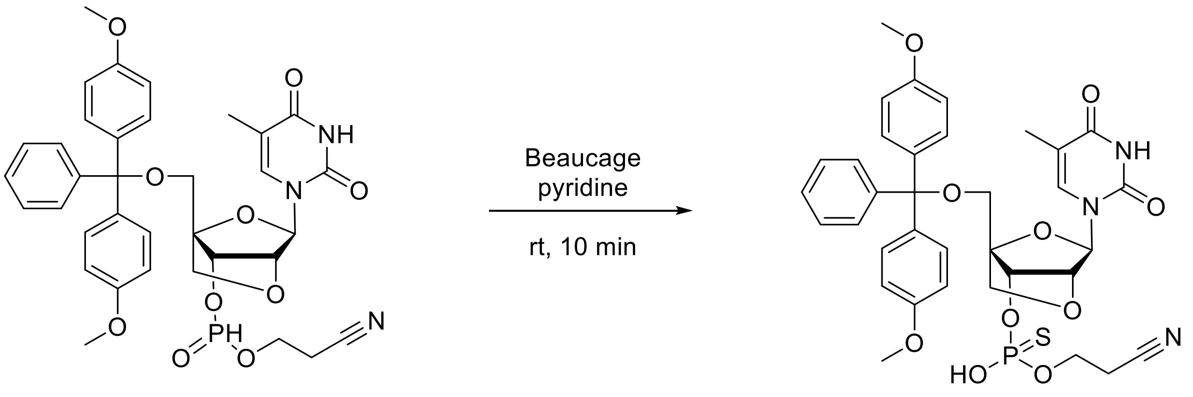

7 **13**

H-phosphonate **7** (150 mg, 0.194 mmol, 1eq) was dissolved in pyridine and the Beaucage's reagent was added (77 mg, 0.388 mmol, 2 eq). The mixture was stirred at room temperature for 10 minutes. Water was added to the reaction mixture (10 mL) and the product was extracted with DCM (3 x 20 mL). The organic phase was concentrated and nucleotide **13** was used without further purification.

^{31}P NMR (161.62 MHz, $CDCl_3$) δ 56.13 (s, 1P).

HRMS (ESI): $[C_{35}H_{35}N_3O_{10}PS]^-$: calcd: 720.1786; found: 720.1798.

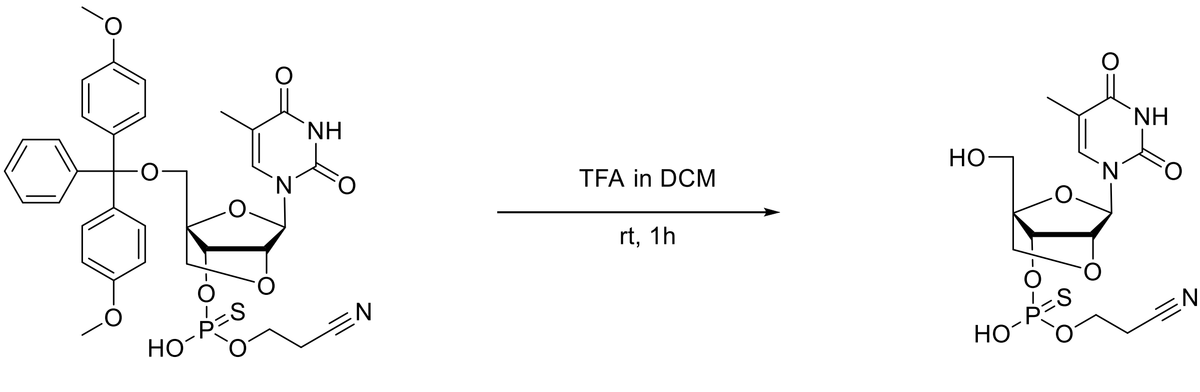

14

13

The starting material **13** was dissolved in DCM (3 mL) and TFA was added (0.5 mL). The reaction mixture was stirred at room temperature for 1h. The solvent was removed *in vacuo*. The crude was dissolved in water and washed with DCM. Nucleotide **14** was used without further purification.

^{1}H NMR (500.13 MHz, MeOD) δ 1.89 (s, 3H), 2.76-2.80 (m, 2H), 3.81 (d, *J* = 8.00 Hz, 1H), 3.91 (d, *J* = 13.20 Hz, 1H), 3.99-4.03 (m, 2H), 4.06-4.12 (m, 2H), 4.60-4.66 (m, 2H), 5.59 (d, *J* = 7.40 Hz, 1H), 7.78 (s, 1H).

^{13}C NMR (100.62 MHz, MeOD) δ 13.9, 21.2, 58.9, 63.4, 74.2, 74.8, 75.3, 80.9, 89.5, 112.3, 120.5, 137.9, 153.2, 167.7.

^{31}P NMR (161.62 MHz, MeOD) δ 54.07.

HRMS (ESI) for $C_{14}H_{17}N_3O_8PS^-$ m/z calcd: 418.0479; found: 418.0468.

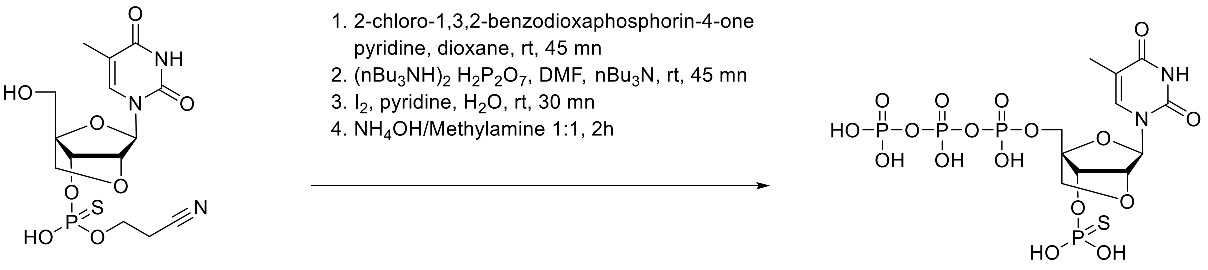

14 **15**

Starting material **14** was coevaporated twice with pyridine and dried under reduced pressure overnight before the reaction. Tributylammonium pyrophosphate was dried under reduced pressure overnight before the reaction. Starting material **14** (80 mg, 0.190 mmol, 1 eq) was dissolved in dry pyridine (0.5 mL) and dried dioxane (0.6 mL) at room temperature under inert atmosphere. To this solution, 2-chloro-1,3,2-benzodioxaphosphorin-4-one (55 mg, 0.267, 1.4 eq) was added and the reaction mixture was stirred for 45 min. A solution of tributylammonium pyrophosphate (135 mg, 0.247 mmol, 1.3 eq), in dry DMF (0.5 mL) and tributylamine (0.3 mL) was added dropwise and the reaction mixture was stirred for another 45 min. It was then oxidized by the addition of iodine (77 mg, 0.304 mmol, 1.6 eq) in pyridine (1 mL) and H_2O (0.4 mL). After 30 min of stirring, the excess of iodine was quenched with a sodium thiosulfate solution (10% w/v in water) and the solution was concentrated under reduced pressure at 30°C. NH_4OH (5 mL) and methylamine (5 mL) were added to the crude mixture and it was stirred at room temperature for 2h. The suspension was then concentrated under reduced pressure at 30°C. The residue was dissolved in H_2O and precipitated by the addition of $NaClO_4$ 2% in acetone. The crude product was purified by HPLC. Buffer A: 10 mM TEAB, Buffer B: 1mM TEAB. R_T = 21', 52%. Yield: 5% yield (over 4 steps).

^{1}H NMR (500.13 MHz, D_2O) δ 1.81 (s, 3H), 3.92-4.03 (m, 2H), 4.35-4.40 (m, 2H), 4.54 (s, 1H), 5.59 (s, 1H), 7.63 (s, 1H).

^{31}P NMR (500.13 MHz, D_2O) δ -25.30 (bs, 1P), -14.30 (bs, 1P), -10.45 (bs, 1P), 40.38 (s, 1P).

HRMS (ESI) for $C_{11}H_{17}N_2O_{17}P_4S^-$ m/z calcd: 604.9204; found: 604.9204.

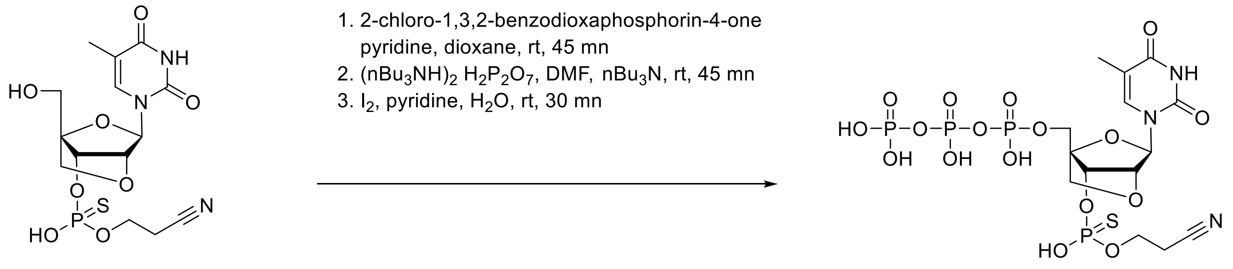

Starting material **14** was coevaporated twice with pyridine and dried under reduced pressure overnight before the reaction. Tributylammonium pyrophosphate was dried under reduced pressure overnight before the reaction. Starting material **14** (54 mg, 0.128 mmol, 1 eq) was dissolved in dry pyridine (0.4 mL) and dried dioxane (0.5 mL) at room temperature under inert atmosphere. To this solution, 2-chloro-1,3,2-benzodioxaphosphorin-4-one (37 mg, 0.179, 1.4 eq) was added and the reaction mixture was stirred for 45 min. A solution of tributylammonium pyrophosphate (91 mg, 0.166 mmol, 1.3 eq), in dry DMF (0.4 mL) and tributylamine (0.2 mL) was added dropwise and the reaction mixture was stirred for another 45 min. It was then oxidized by the addition of iodine (52 mg, 0.205 mmol, 1.6 eq) in pyridine (1 mL) and H_2O (0.3 mL). After 30 min of stirring, the excess of iodine was quenched with a sodium thiosulfate solution (10% w/v in water) and the solution was concentrated under reduced pressure at 30°C. The suspension was then concentrated under reduced pressure at 30°C. The residue was dissolved in H_2O and precipitated by the addition of $NaClO_4$ 2% in acetone. The crude product was purified by HPLC. Buffer A: 10 mM TEAB, Buffer B: 1mM TEAB. R_T = 22', 56%. 7% yield (over 4 steps)

^{1}H NMR (500.13 MHz, D_2O) δ 1.46-1.54 (m, 2H), 1.74 (s, 3H), 2.69-2.73 (m, 2H), 2.89-2.93 (m, 2H), 3.93-4.02 (m, 2H), 4.29 (bs, 1H), 4.34 (bs, 1H), 5.57 (bs, 1H), 7.60 (bs, 1H).

^{31}P NMR (161.62 MHz, D_2O) δ -25.46 (bs, 2P), -14.6 (bs, 2P), -9.43 (bs, 2P), 51.77 (s, 1P), 52.01 (s, 1P).

HRMS (ESI) for $C_{14}H_{20}N_3O_{17}P_4S^-$ m/z calcd: 657.9469; found: 657.9473.

5. Characterization of nucleosides and nucleotides

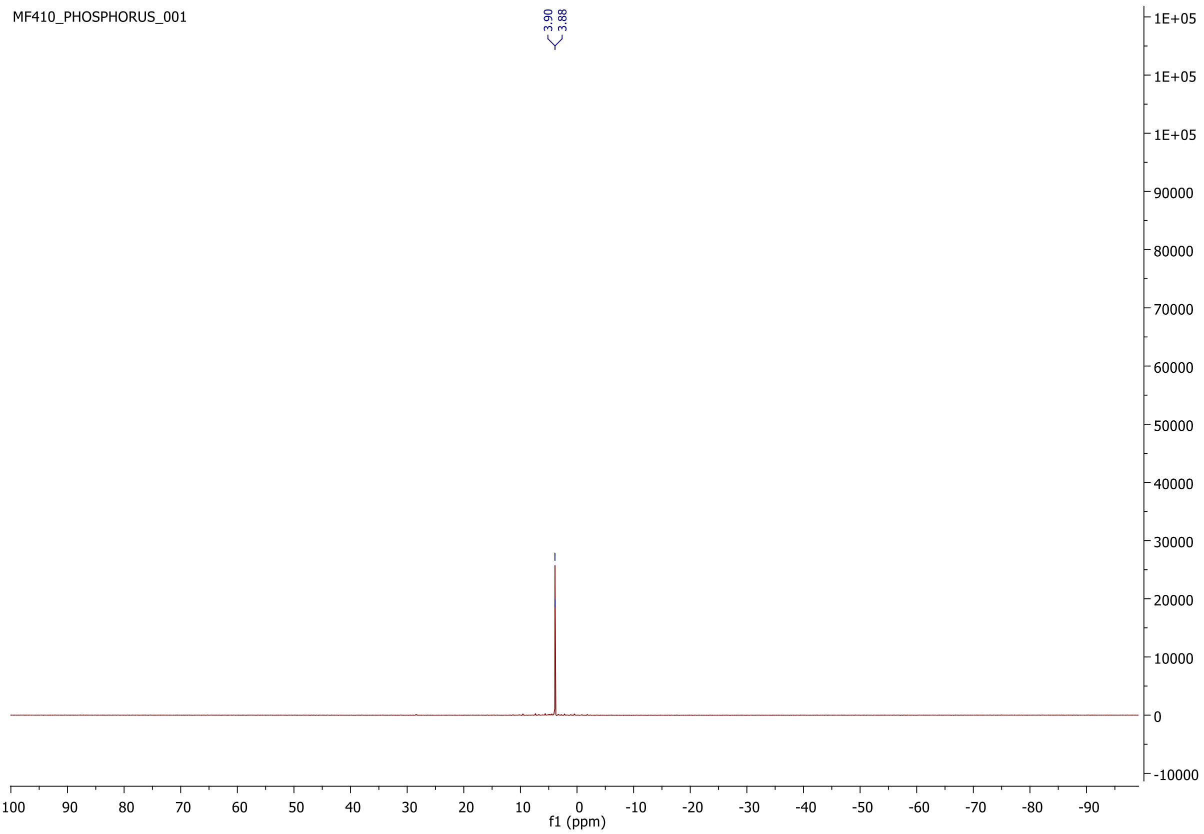

Supplementary Figure 46. ^{31}P NMR (161.62 MHz, $CDCl_3$) spectrum of compound **2**.

Analysis Info

Analysis Name	D:\Data\LH\mf410pos000001.d	Acquisition Date	07/04/2012 06:45:26
Method	TUNE POS wide.m	Operator	Nicolas Molinier
Sample Name	mf410pos	Instrument / Ser#	micrOTOF 51
Comment			

Acquisition Parameter

Source Type	ESI	Ion Polarity	Positive	Set Nebulizer	0.3 Bar
Focus	Not active			Set Dry Heater	180 °C
Scan Begin	150 m/z	Set Capillary	4500 V	Set Dry Gas	3.0 l/min
Scan End	1200 m/z	Set End Plate Offset	-500 V	Set Divert Valve	Source

#	RT [min]	Area
n.a.	0.0	n.a.

+MS

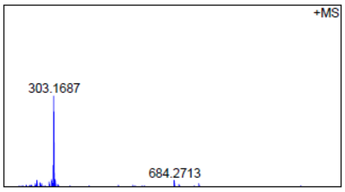

m/z	I	Res.
249.2322	1074	4854
288.1449	768	4559
297.2571	1143	4772
302.2654	3571	4122
303.1687	15059	2588
304.1720	5548	4266
307.7290	761	3855
307.7660	1193	4122
307.7914	1193	4122
684.2713	1048	5173

Supplementary Figure 47. HRMS analysis of compound **2**.

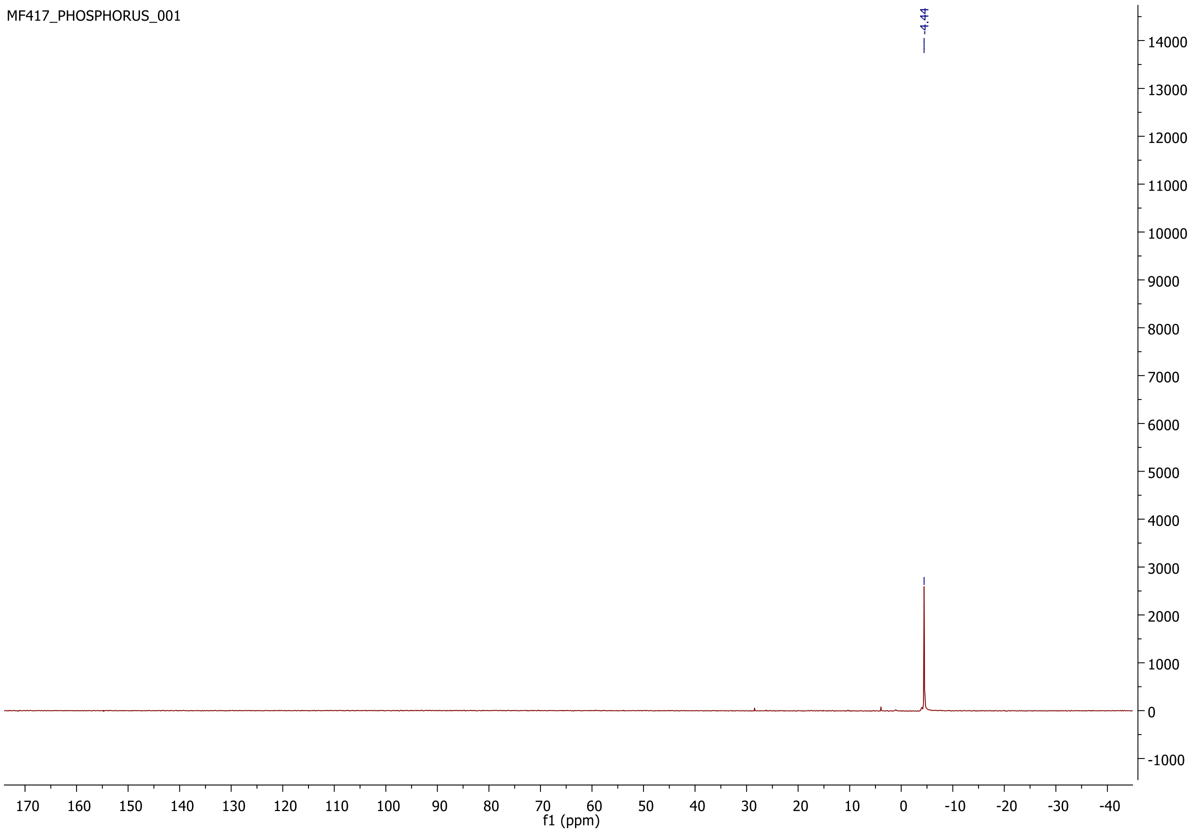

Supplementary Figure S48. ^{31}P NMR (161.62 MHz, $CDCl_3$) of compound **3**.

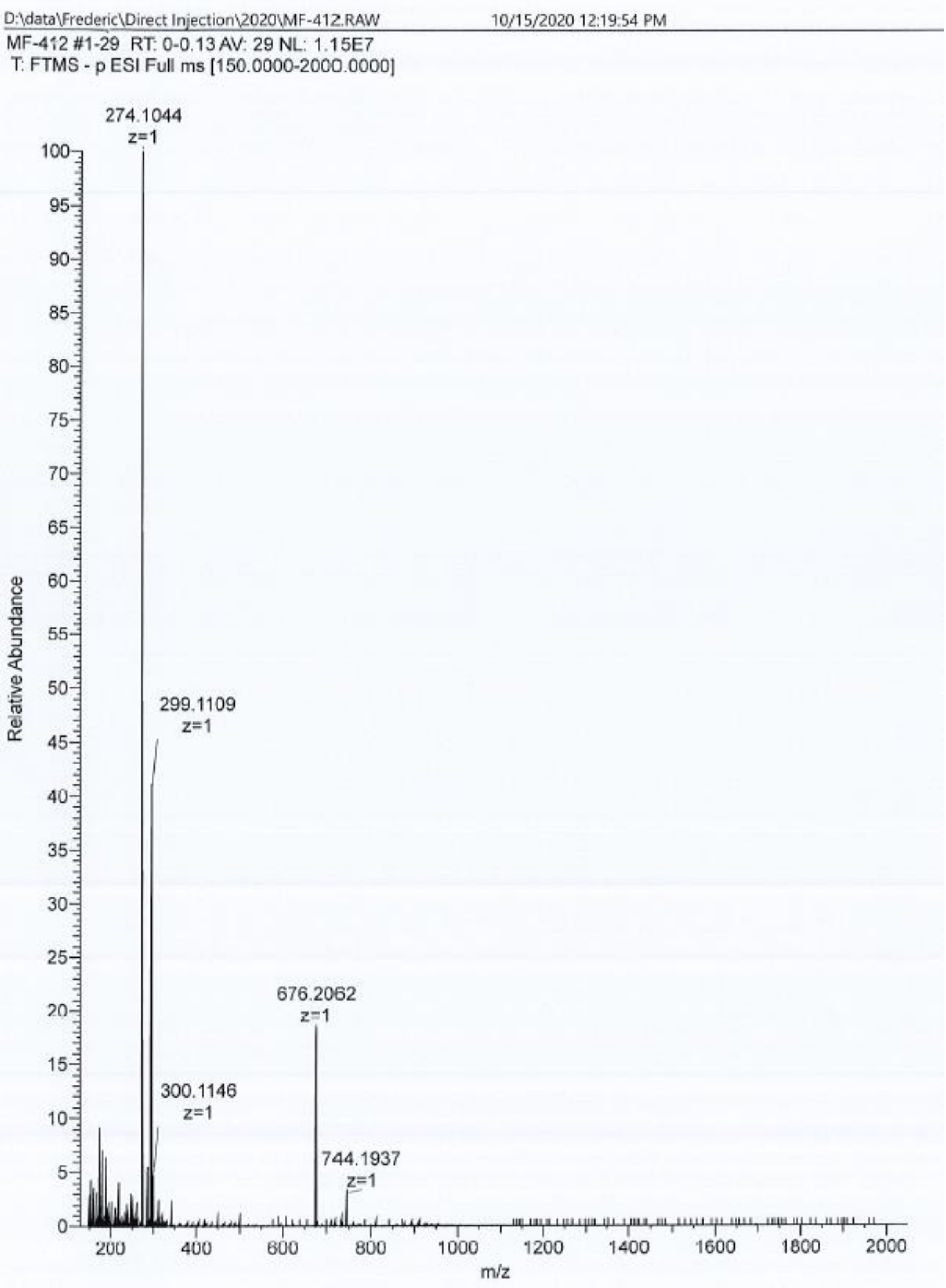
D:\data\Frederic\Direct Injection\2020\MF-412.RAW
10/15/2020 12:19:54 PM
MF-412 #1-29 RT: 0-0.13 AV: 29 NL: 1.15E7
T: FTMS - p ESI Full ms [150.0000-2000.0000]
274.1044
z=1
299.1109
z=1
300.1146
z=1
676.2062
z=1
744.1937
z=1
Relative Abundance
m/z

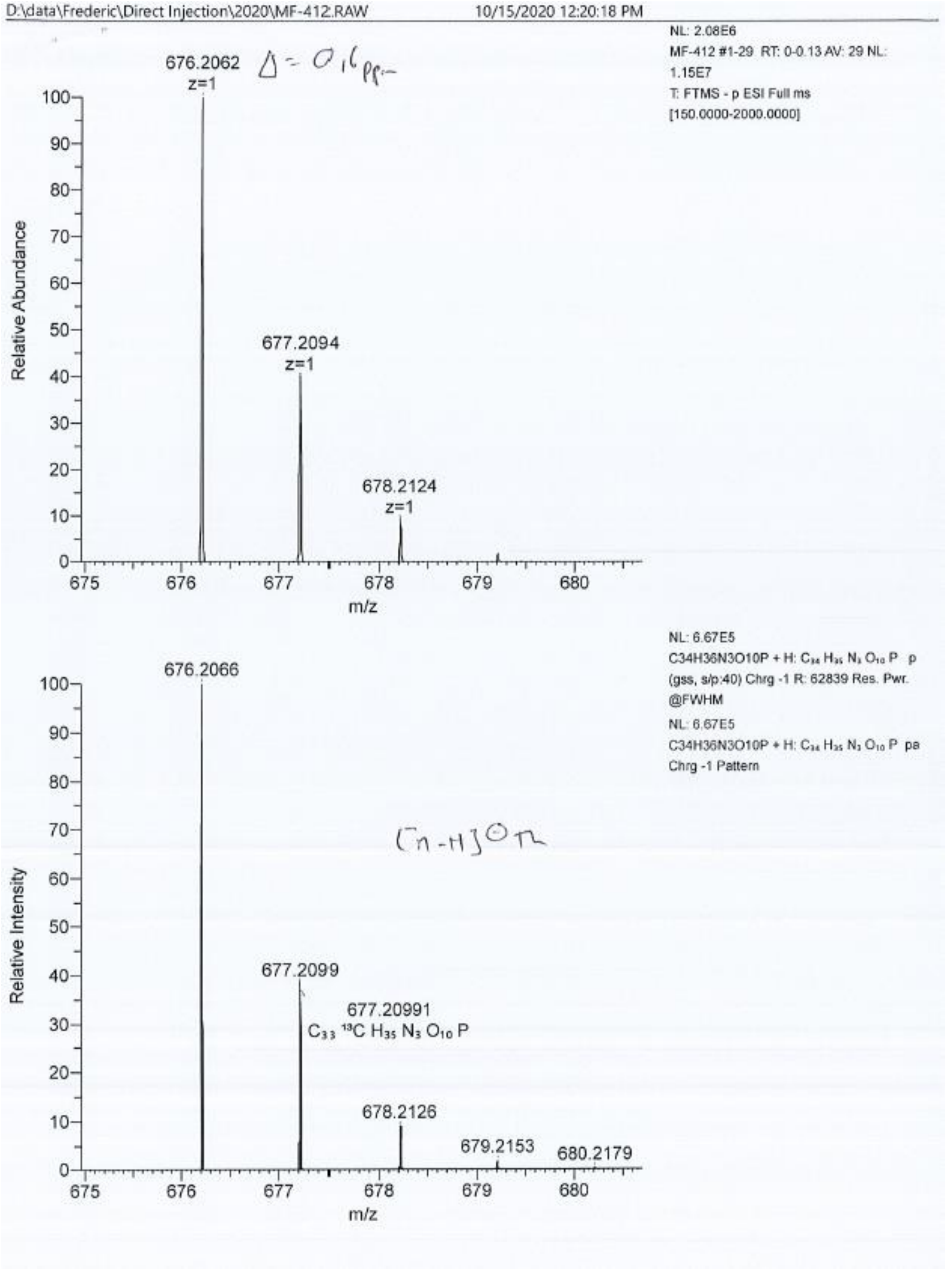

Supplementary Figure 49. HRMS analysis of compound **3**.

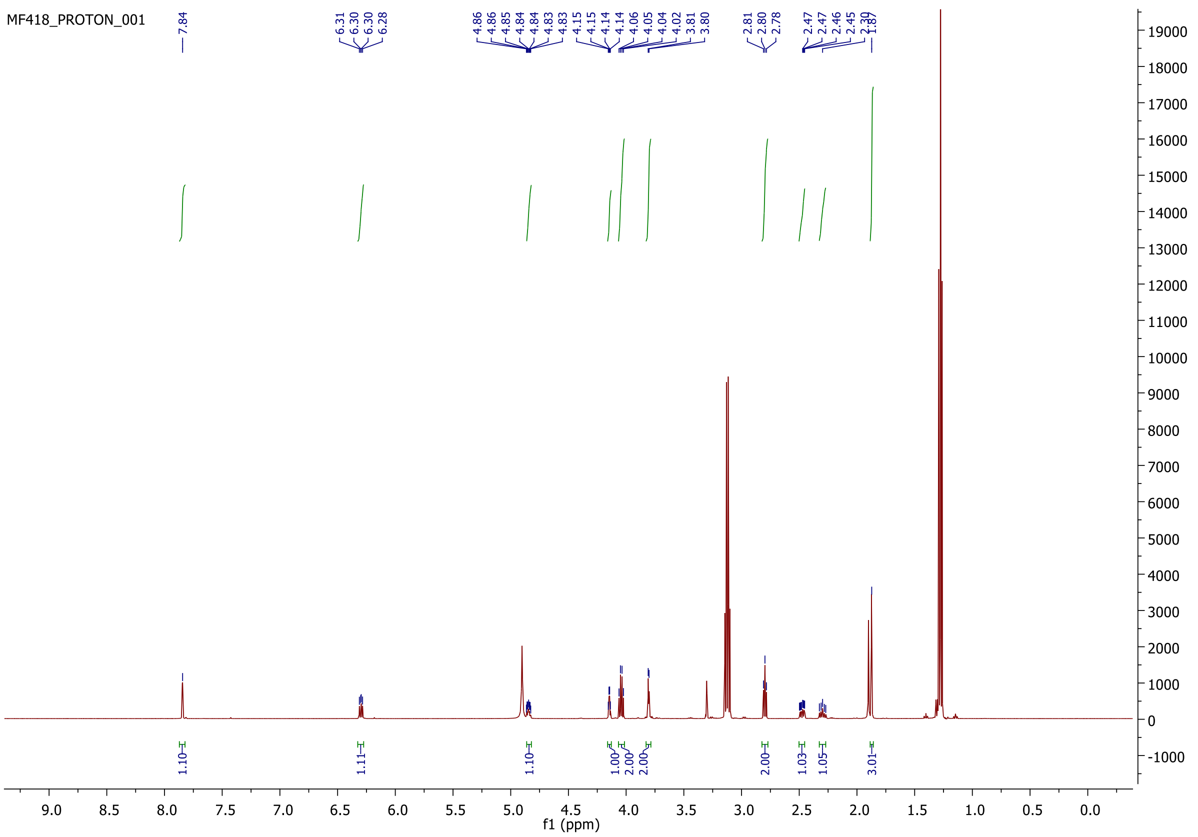

Supplementary Figure 50. ^{1}H NMR (500.13 MHz, MeOD) spectrum of compound **4**.

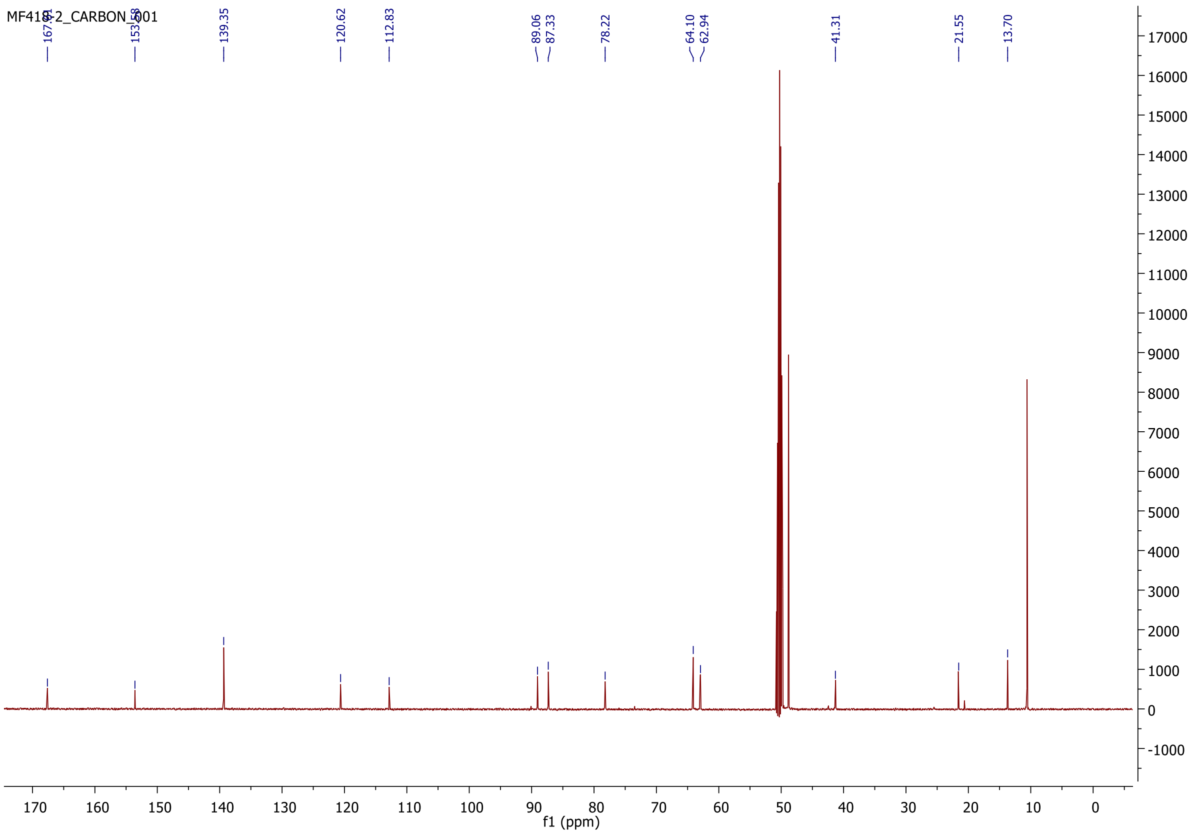

Supplementary Figure 51. ^{13}C NMR (100.62 MHz, MeOD) spectrum of compound **4**.

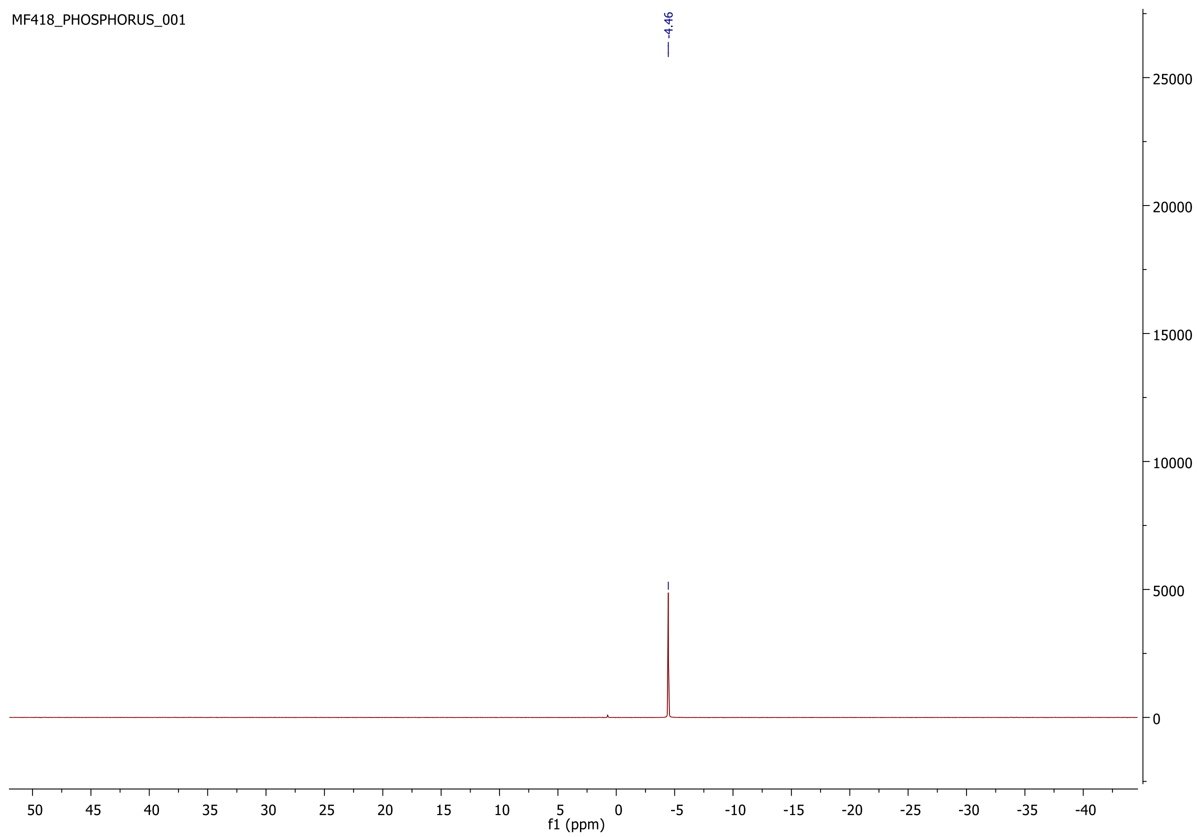

Supplementary Figure 52. ^{31}P NMR (161.62 MHz, MeOD) spectrum of compound **4.**

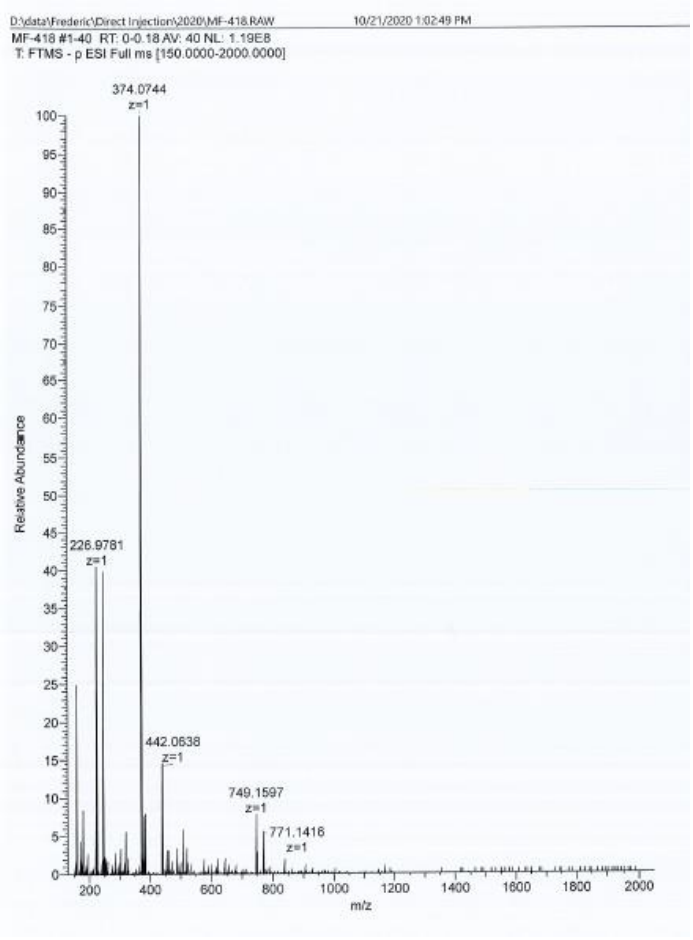

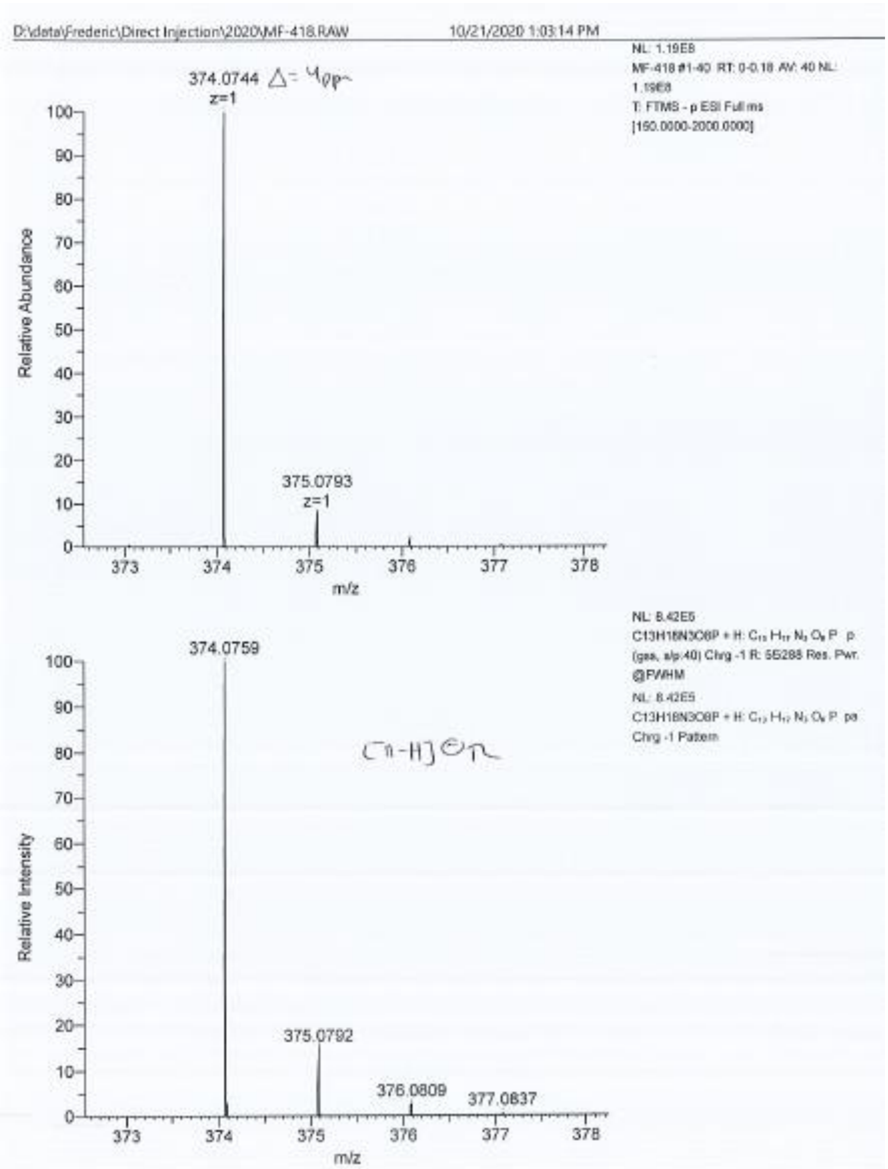

Supplementary Figure 53. HRMS analysis of compound **4**.

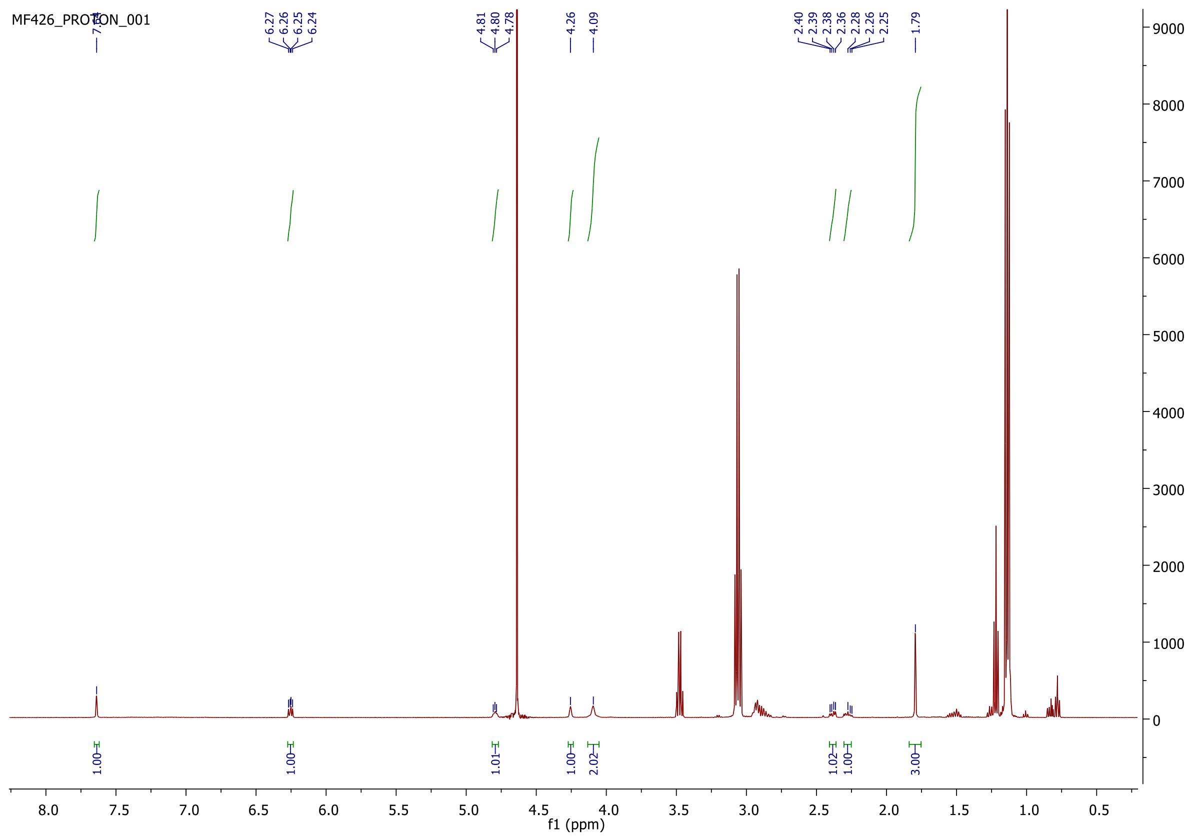

Supplementary Figure 54. ^{1}H NMR (400.13 MHz, D_2O) spectrum of compound **5**.

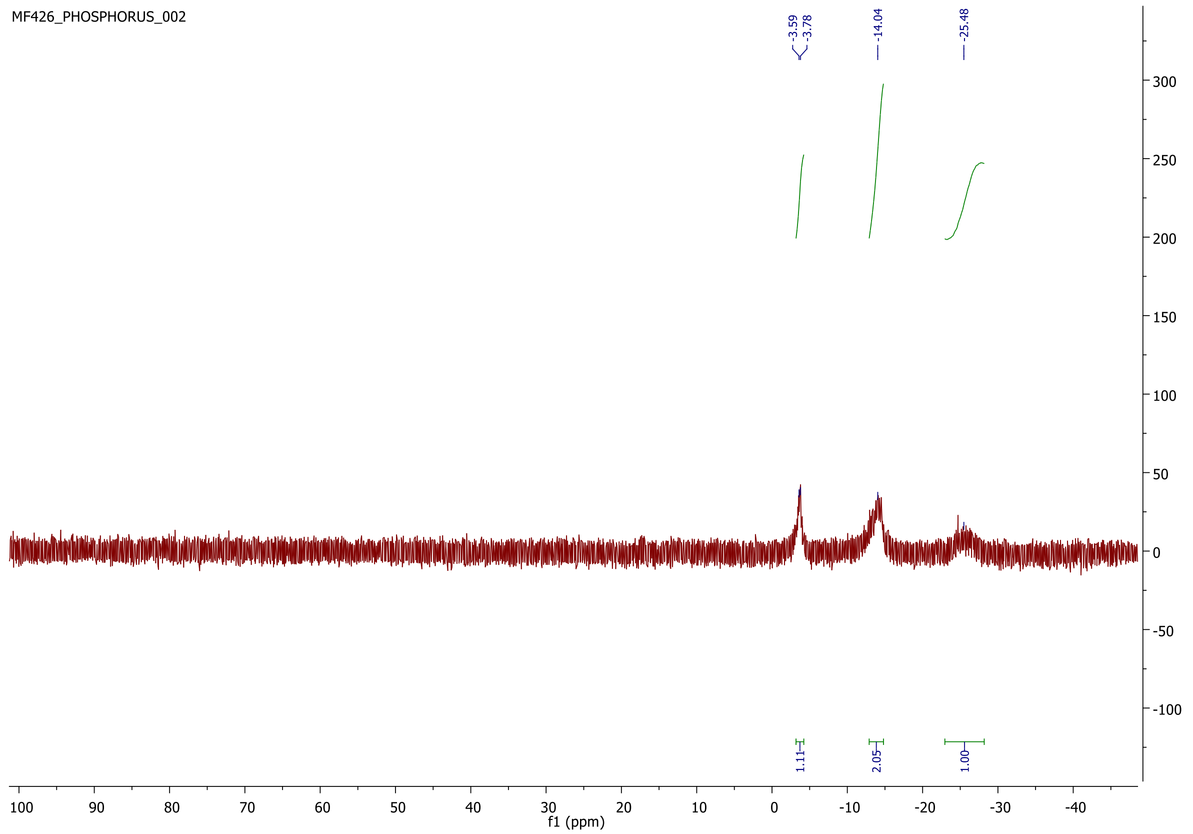

Supplementary Figure 55. ^{31}P NMR (161.62 MHz, D_2O) spectrum of compound **5**.

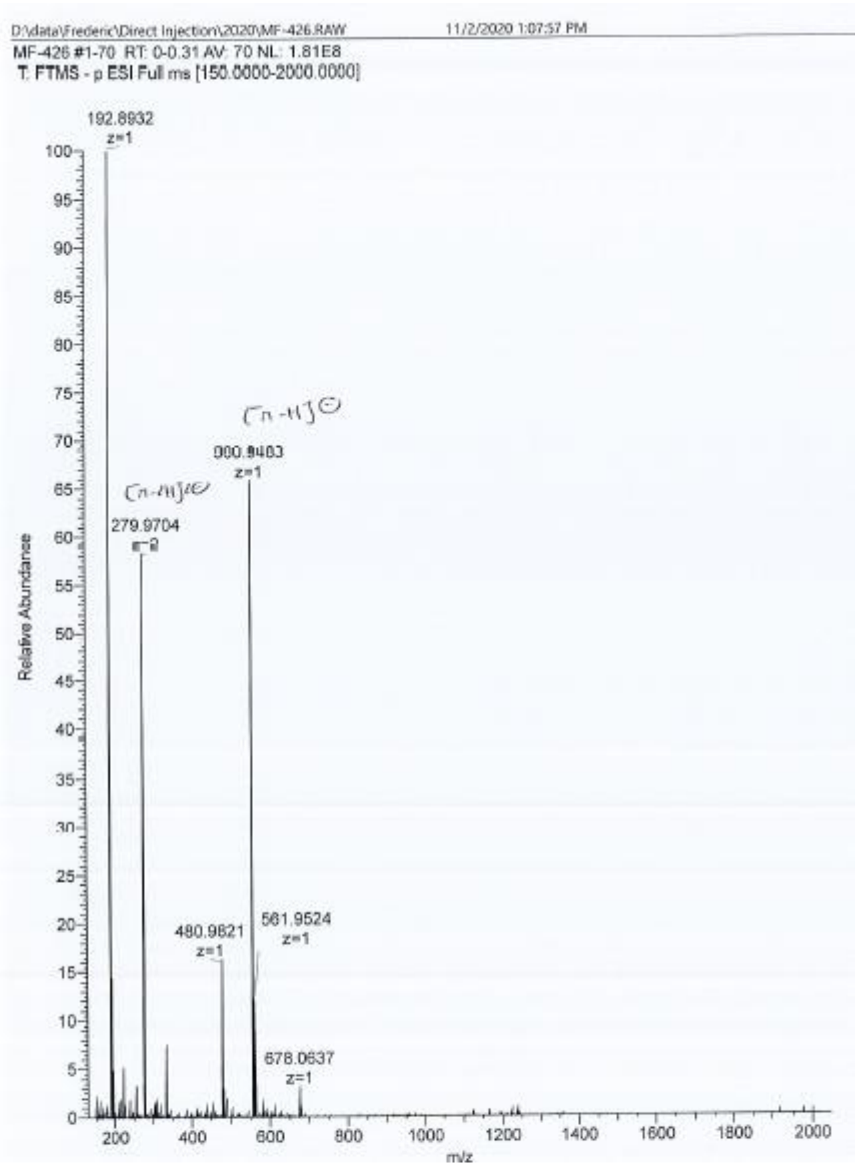
D:\data\Frederic\Direct Injection\2020\MF-426.RAW
11/2/2020 1:07:57 PM
MF-426 #1-70 RT: 0-0.31 AV: 70 NL: 1.81E8
T: FTMS - p ESI Full ms [150.0000-2000.0000]
192.8932
z=1
[n-H]⊖
560.9483
z=1
279.9704
z=2
480.9621
z=1
561.9524
z=1
678.0637
z=1
Relative Abundance
m/z

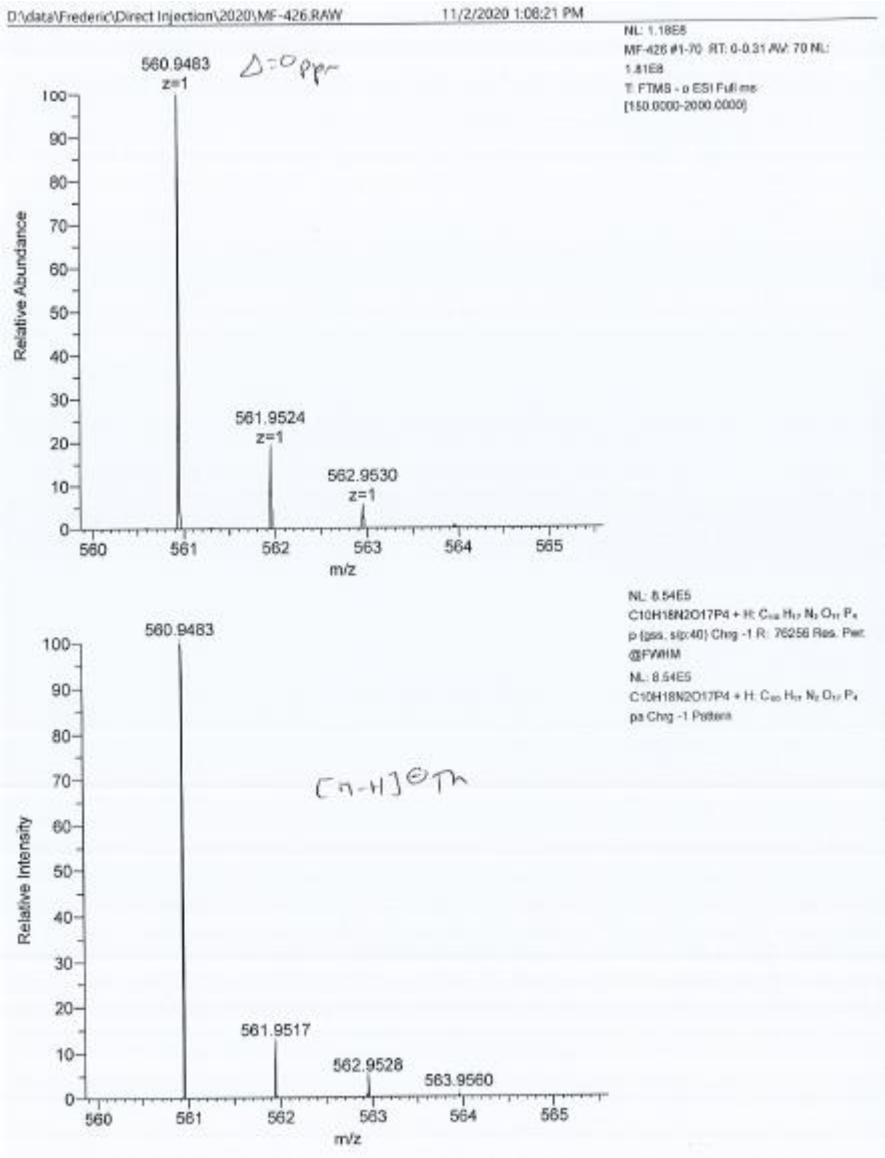

Supplementary Figure 56. HRMS analysis of compound **5**.

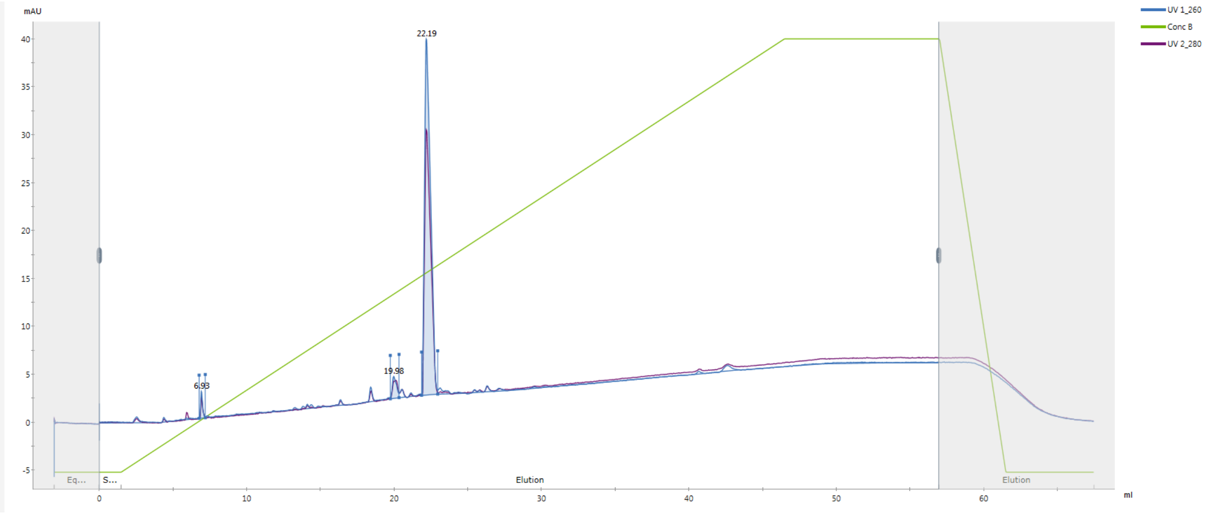

Supplementary Figure 57. HPLC (anion exchange) chromatogram of purified compound **5**.

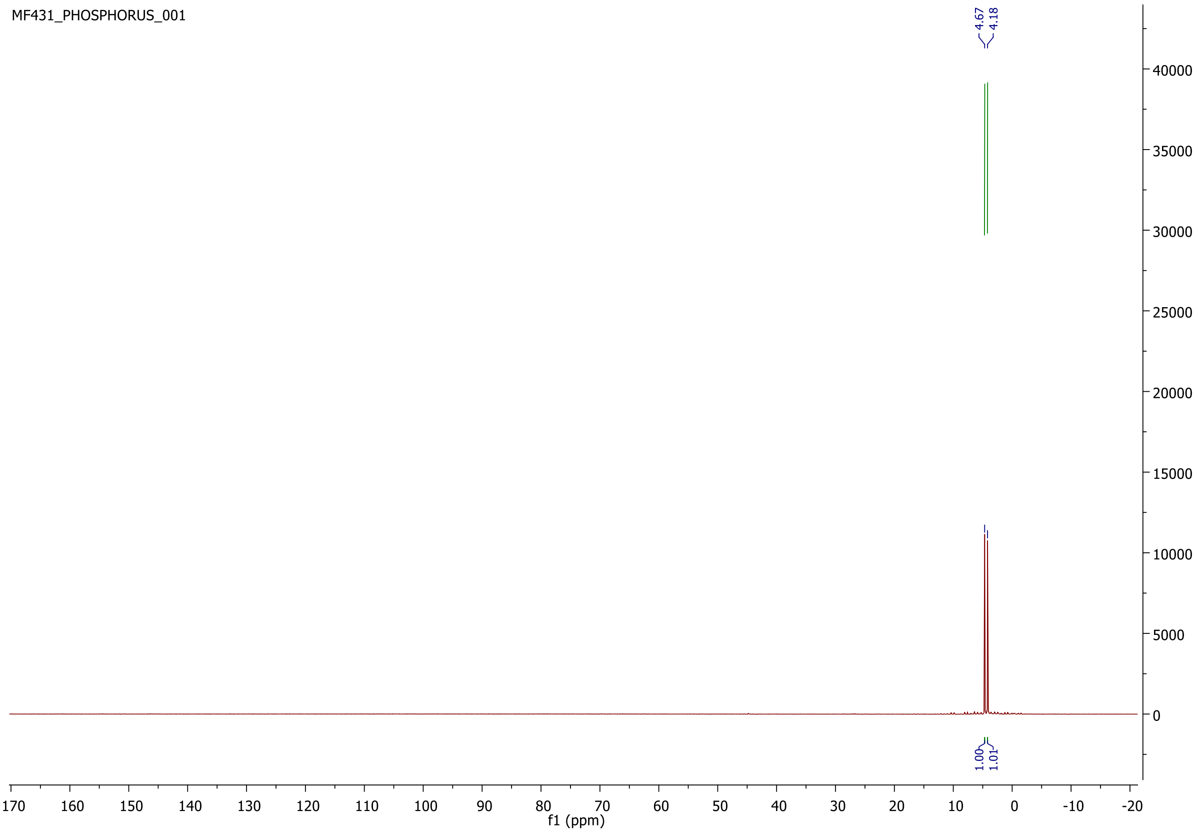

Supplementary Figure 58. ^{31}P NMR (161.62 MHz, $CDCl_3$) spectrum of compound **7**.

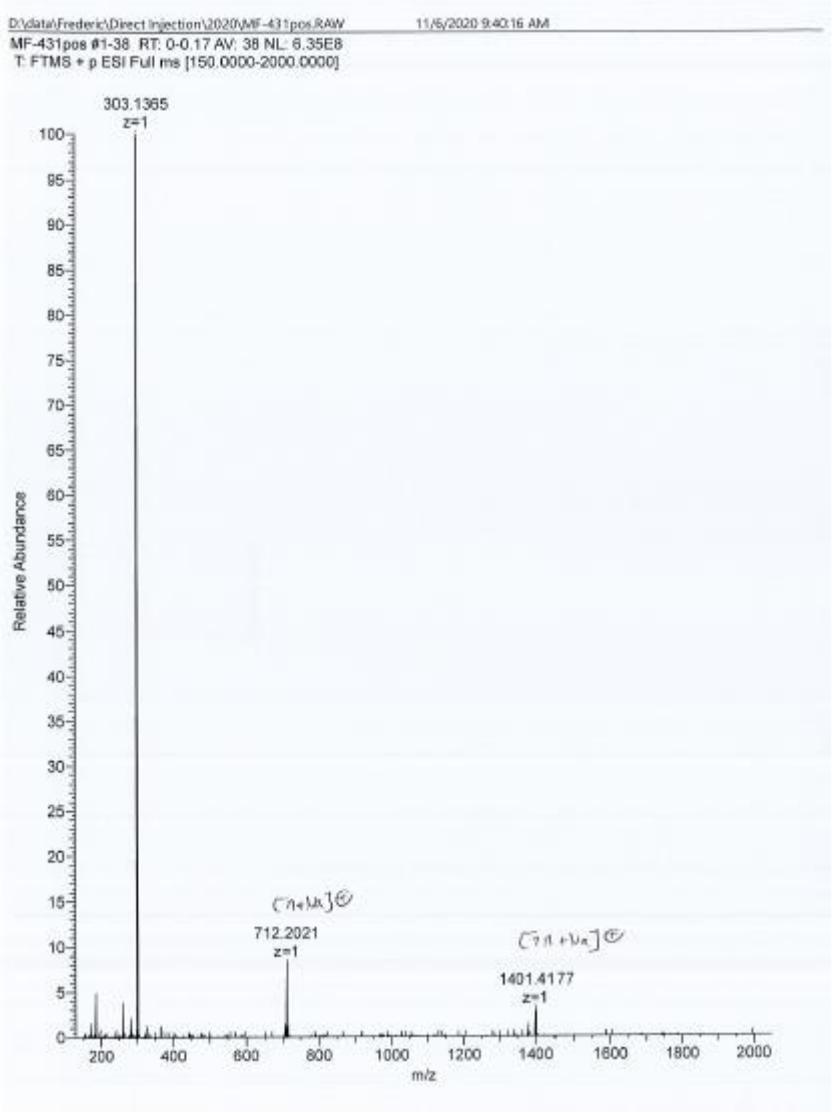

D:\data\Frederic\Direct Injection\2020\MF-431pos.RAW
11/6/2020 9:40:16 AM
MF-431pos #1-38 RT: 0-0.17 AV: 38 NL: 6.35E8
T: FTMS + p ESI Full ms [150.0000-2000.0000]
303.1365
z=1
712.2021
z=1
1401.4177
z=1
Relative Abundance
m/z

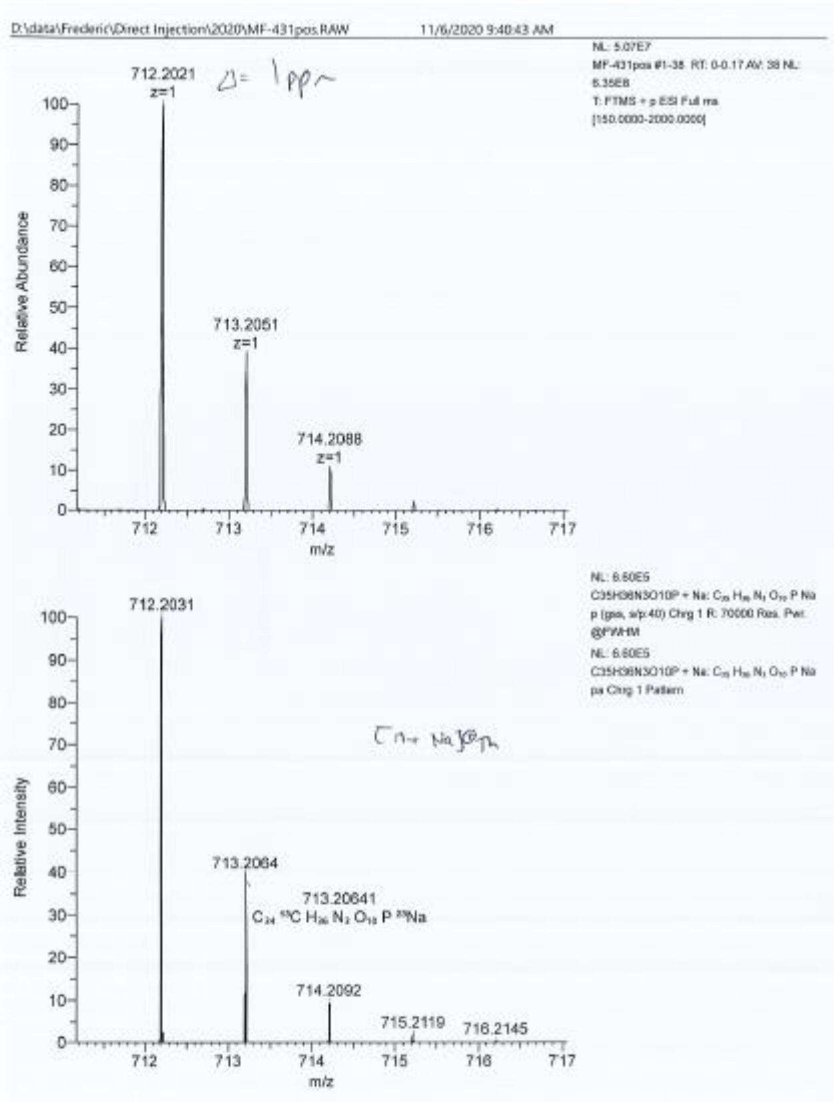

Supplementary Figure 59. HRMS analysis of compound **7**.

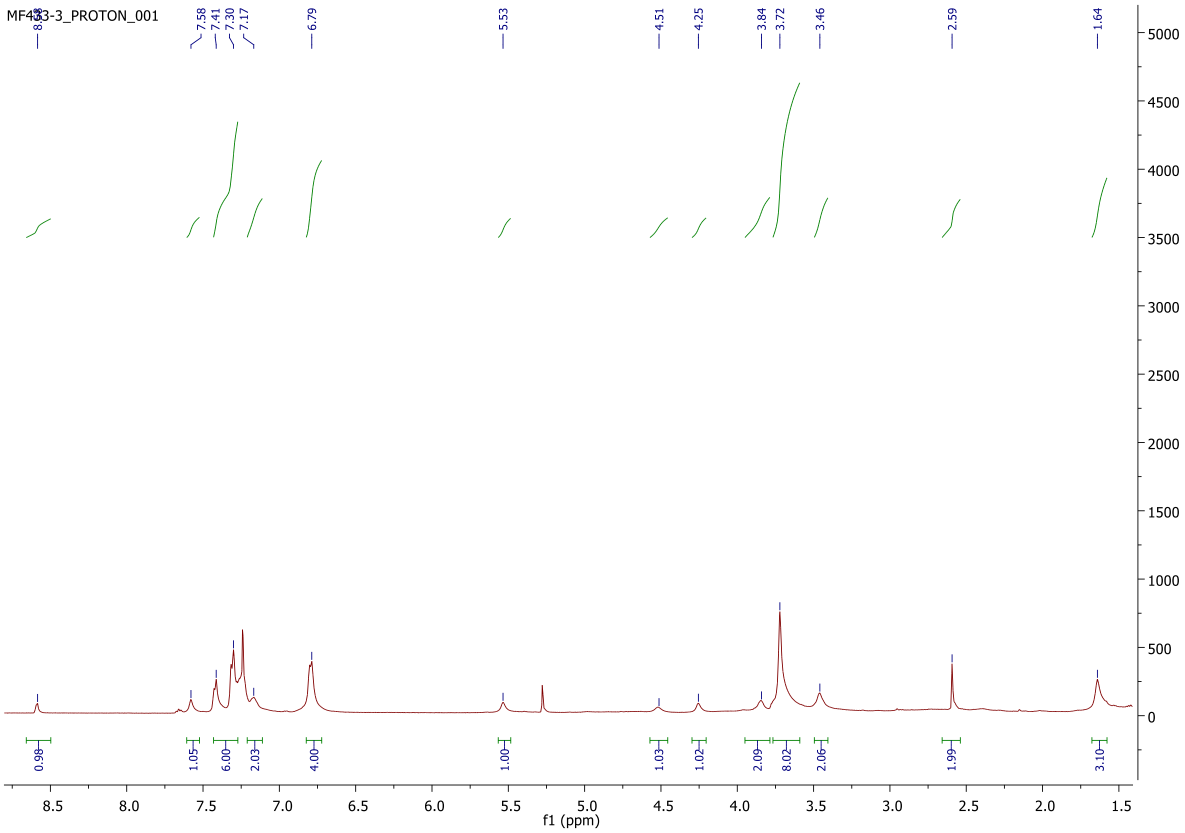

Supplementary Figure 60. ^{1}H NMR (500.13 MHz, $CDCl_3$) spectrum of compound **8**.

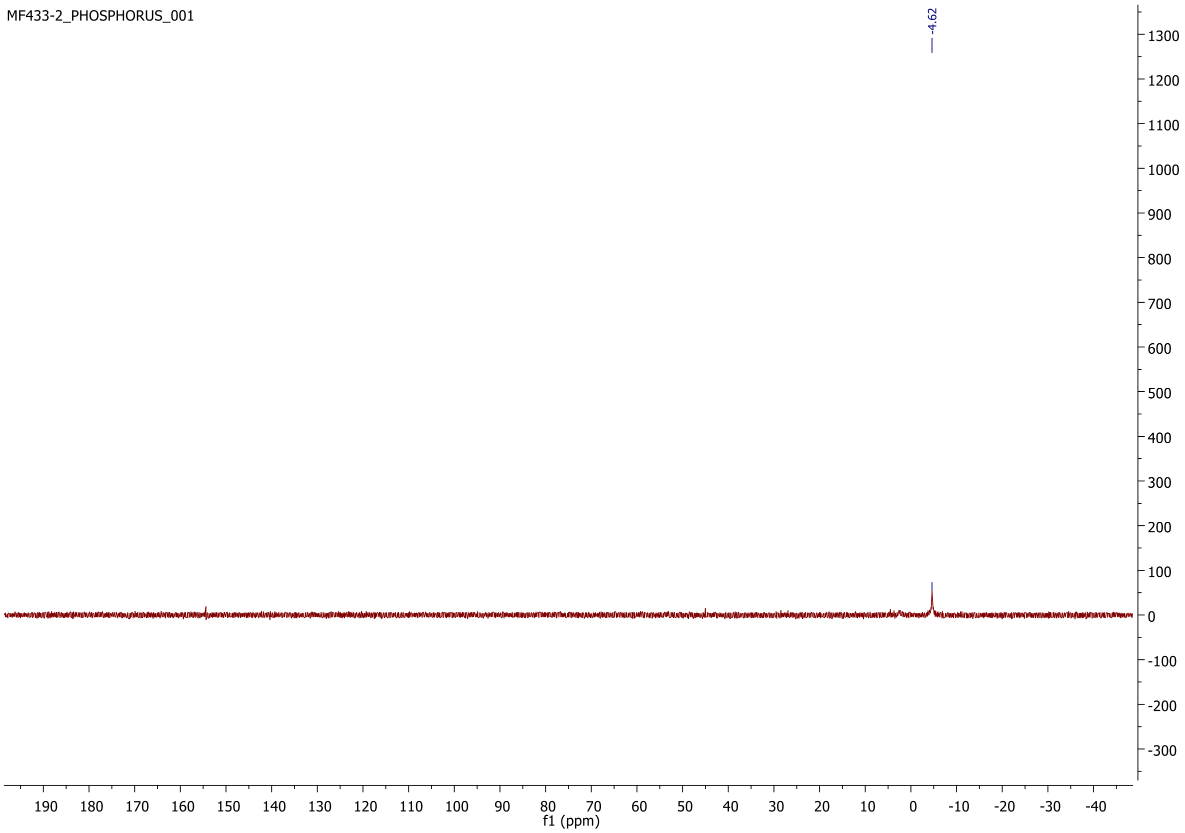

Supplementary Figure 61. ^{31}P NMR (161.62 MHz, $CDCl_3$) spectrum of compound **8**.

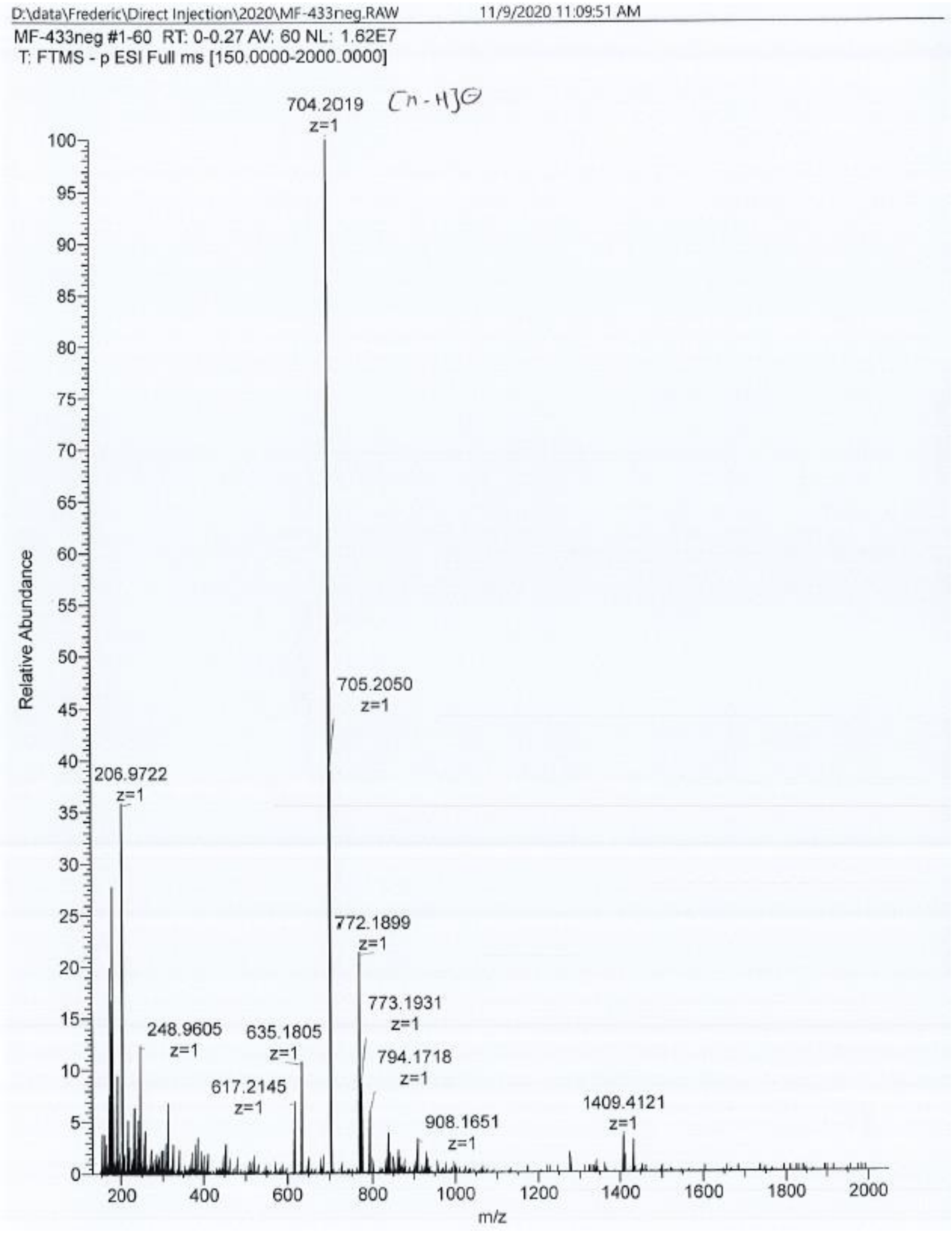

D:\data\Frederic\Direct Injection\2020\MF-433neg.RAW
11/9/2020 11:09:51 AM
MF-433neg #1-60 RT: 0-0.27 AV: 60 NL: 1.62E7
T: FTMS - p ESI Full ms [150.0000-2000.0000]
704.2019 z=1
[M-H]⊖
705.2050 z=1
206.9722 z=1
772.1899 z=1
773.1931 z=1
248.9605 z=1
635.1805 z=1
794.1718 z=1
617.2145 z=1
1409.4121 z=1
908.1651 z=1
Relative Abundance
m/z

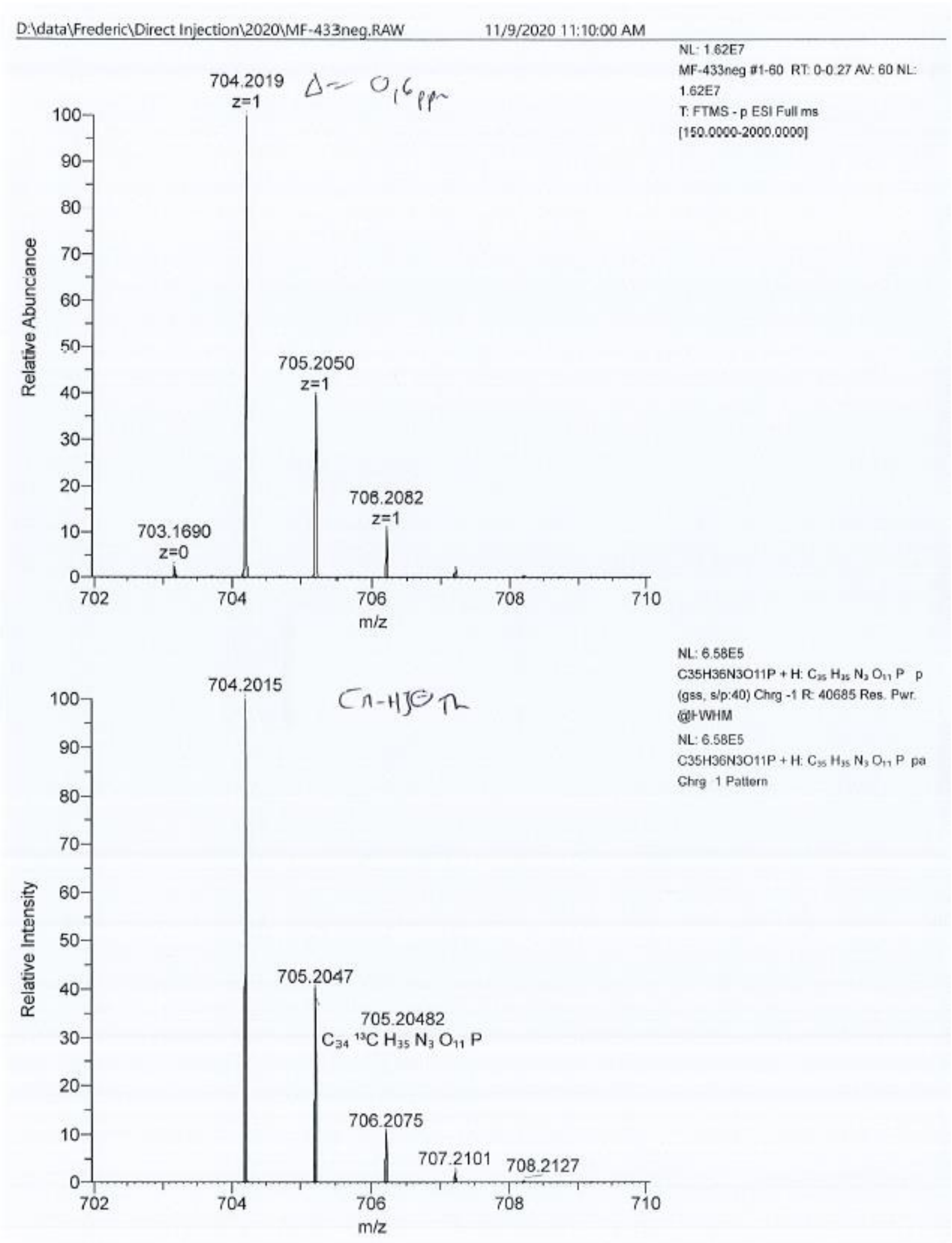

Supplementary Figure 62. HRMS analysis of compound **8**.

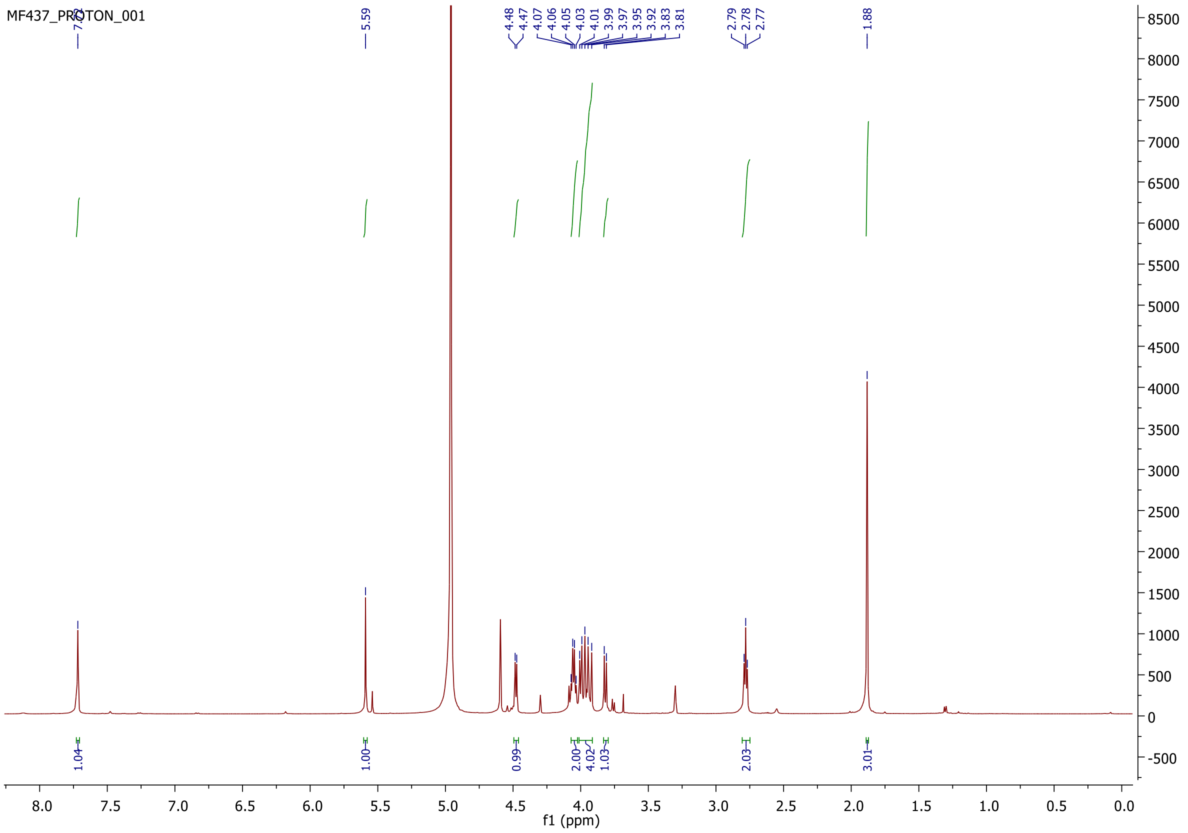

Supplementary Figure 63. ^{1}H NMR (500.13 MHz, MeOD) of compound **9**.

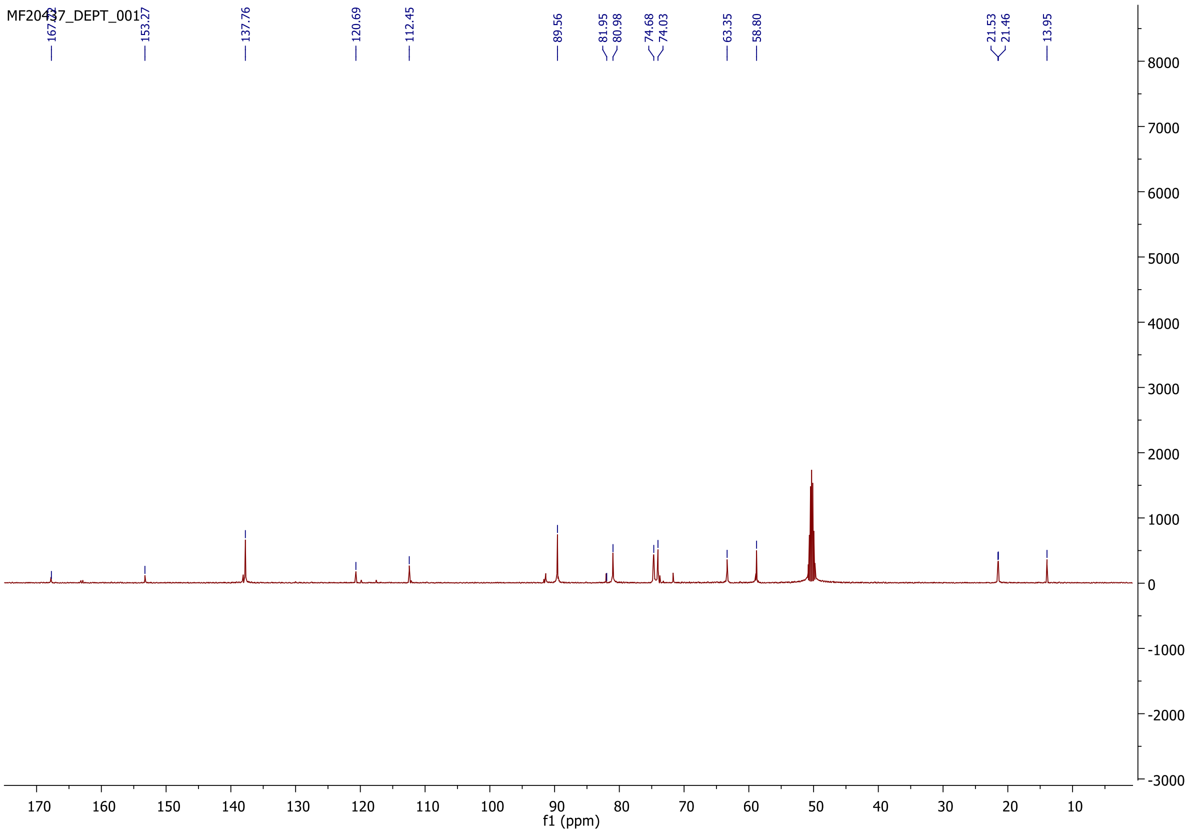

Supplementary Figure 64. ^{13}C NMR (100.62 MHz, MeOD) spectrum of compound **9**.

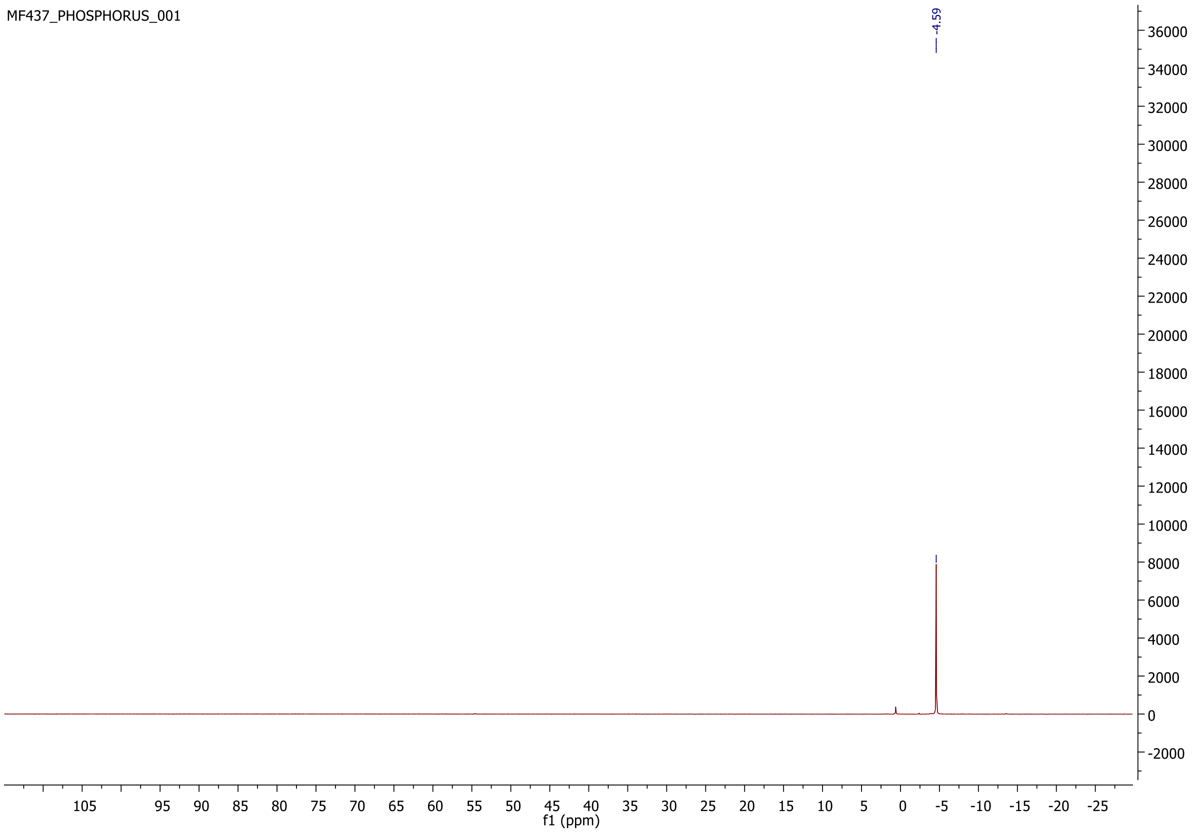

Supplementary Figure 65. ^{31}P NMR (161.62 MHz, MeOD) spectrum of compound **9**.

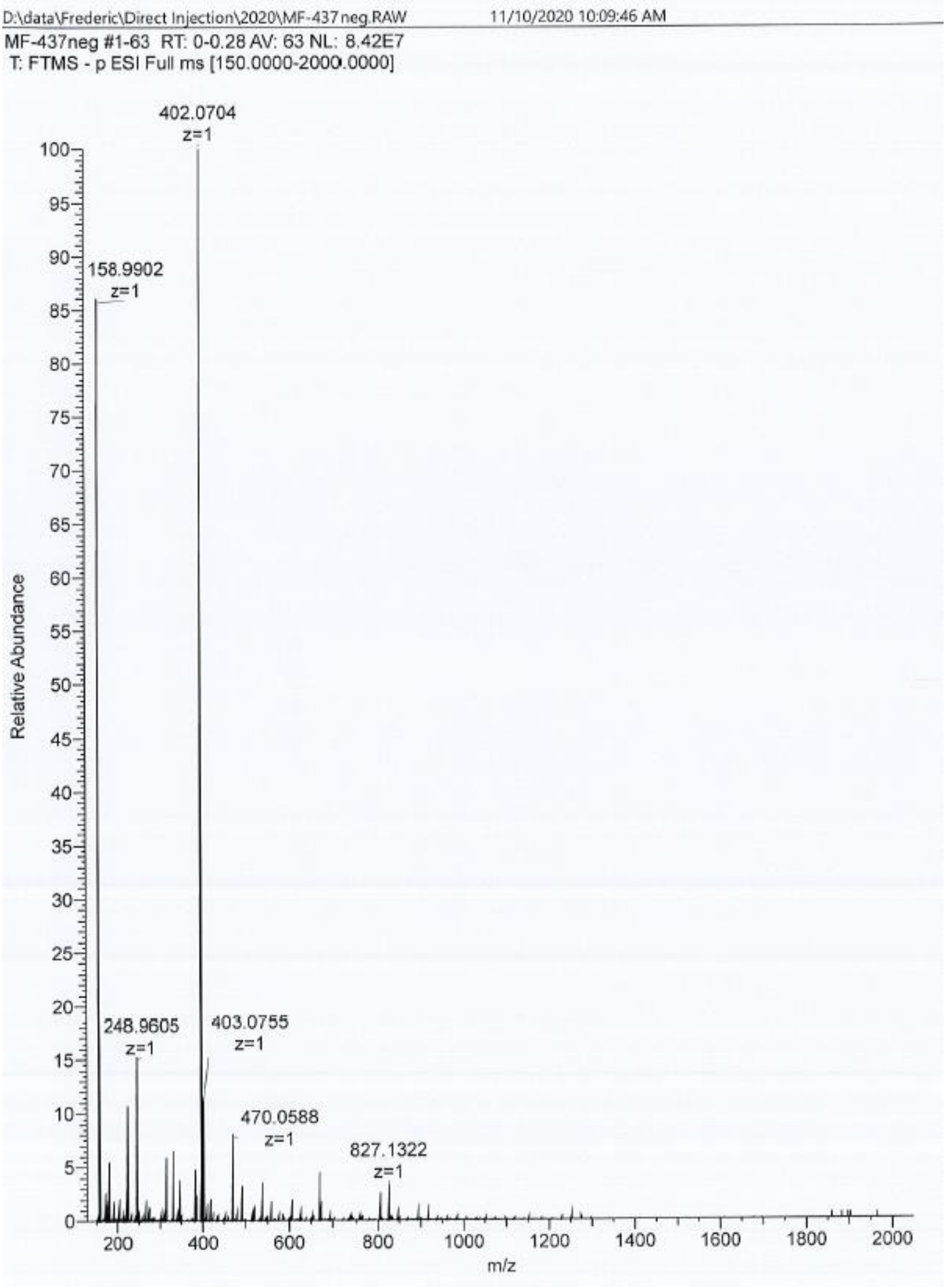

D:\data\Frederic\Direct Injection\2020\MF-437neg.RAW
11/10/2020 10:09:46 AM
MF-437neg #1-63 RT: 0-0.28 AV: 63 NL: 8.42E7
T: FTMS - p ESI Full ms [150.0000-2000.0000]
402.0704
z=1
158.9902
z=1
248.9605
z=1
403.0755
z=1
470.0588
z=1
827.1322
z=1
Relative Abundance
m/z

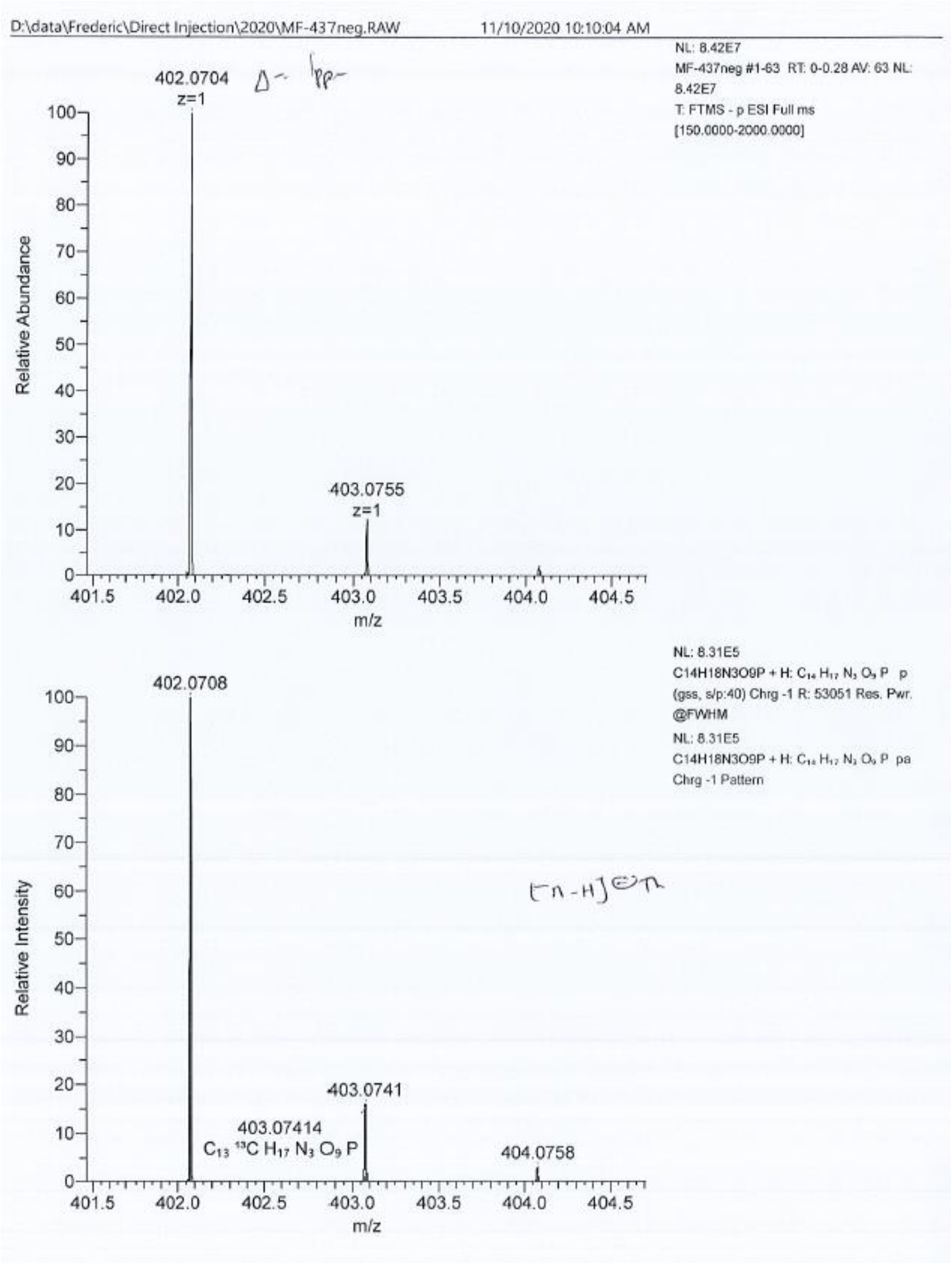

Supplementary Figure 66. HRMS analysis of compound **9**.

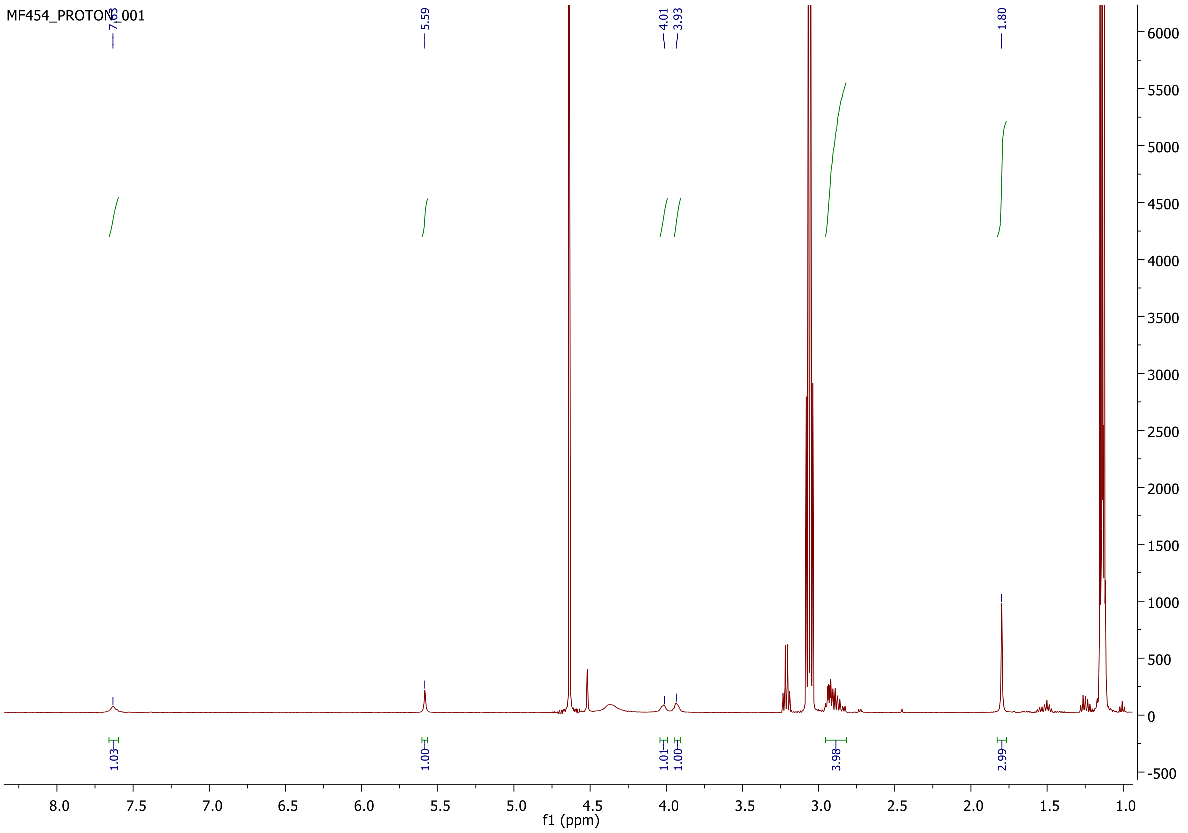

Supplementary Figure 67. ^{1}H NMR (400.13 MHz, D_2O) spectrum of compound **10**.

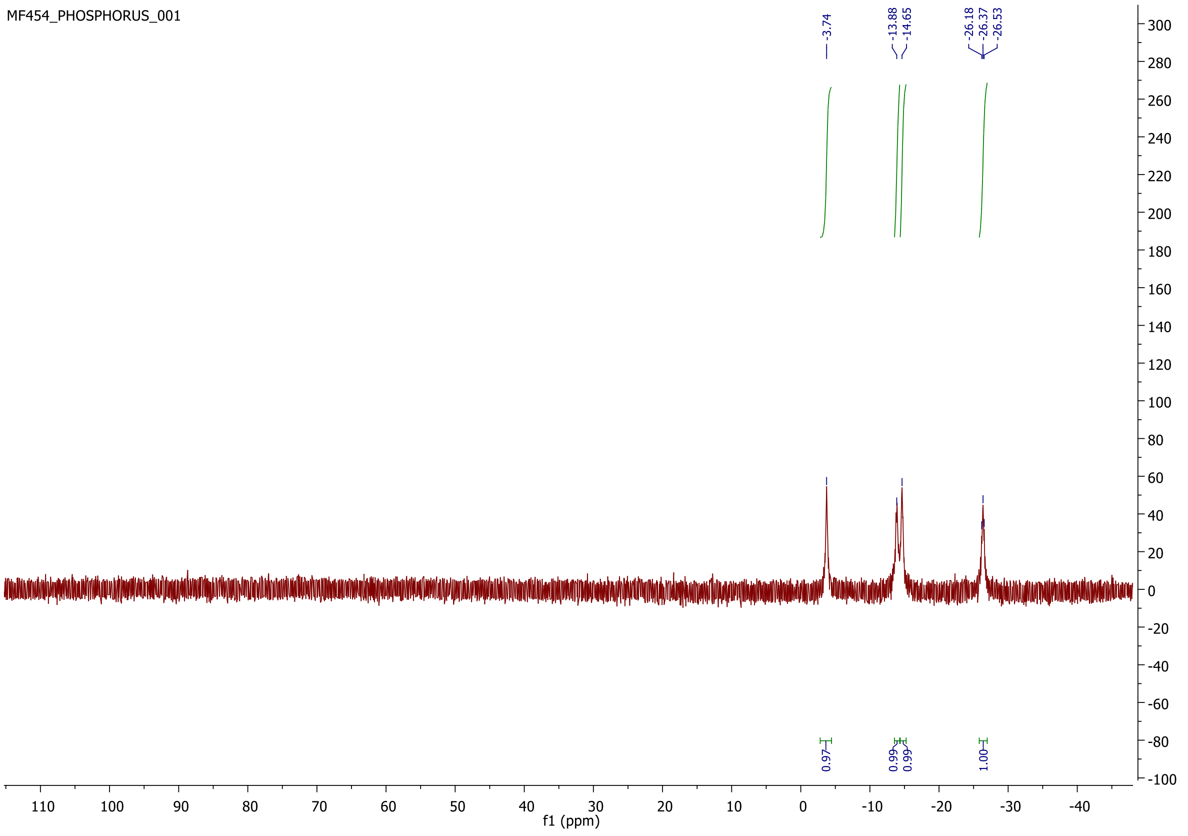

Supplementary Figure 68. ^{31}P NMR (161.62 MHz, D_2O) spectrum of compound **10**.

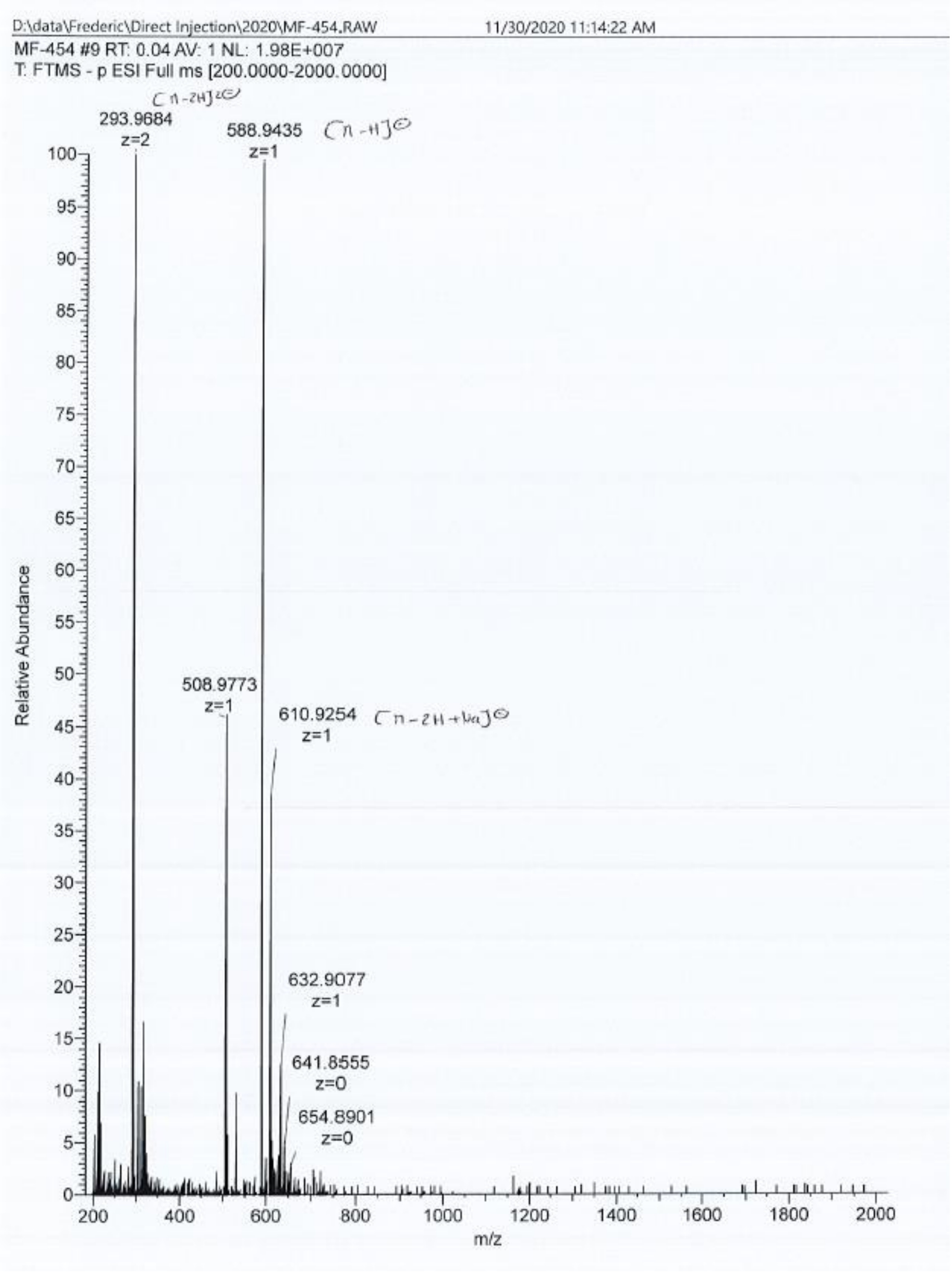
D:\data\Frederic\Direct Injection\2020\MF-454.RAW
11/30/2020 11:14:22 AM
MF-454 #9 RT: 0.04 AV: 1 NL: 1.98E+007
T: FTMS - p ESI Full ms [200.0000-2000.0000]
293.9684
z=2
588.9435
z=1
508.9773
z=1
610.9254
z=1
632.9077
z=1
641.8555
z=0
654.8901
z=0
Relative Abundance
m/z

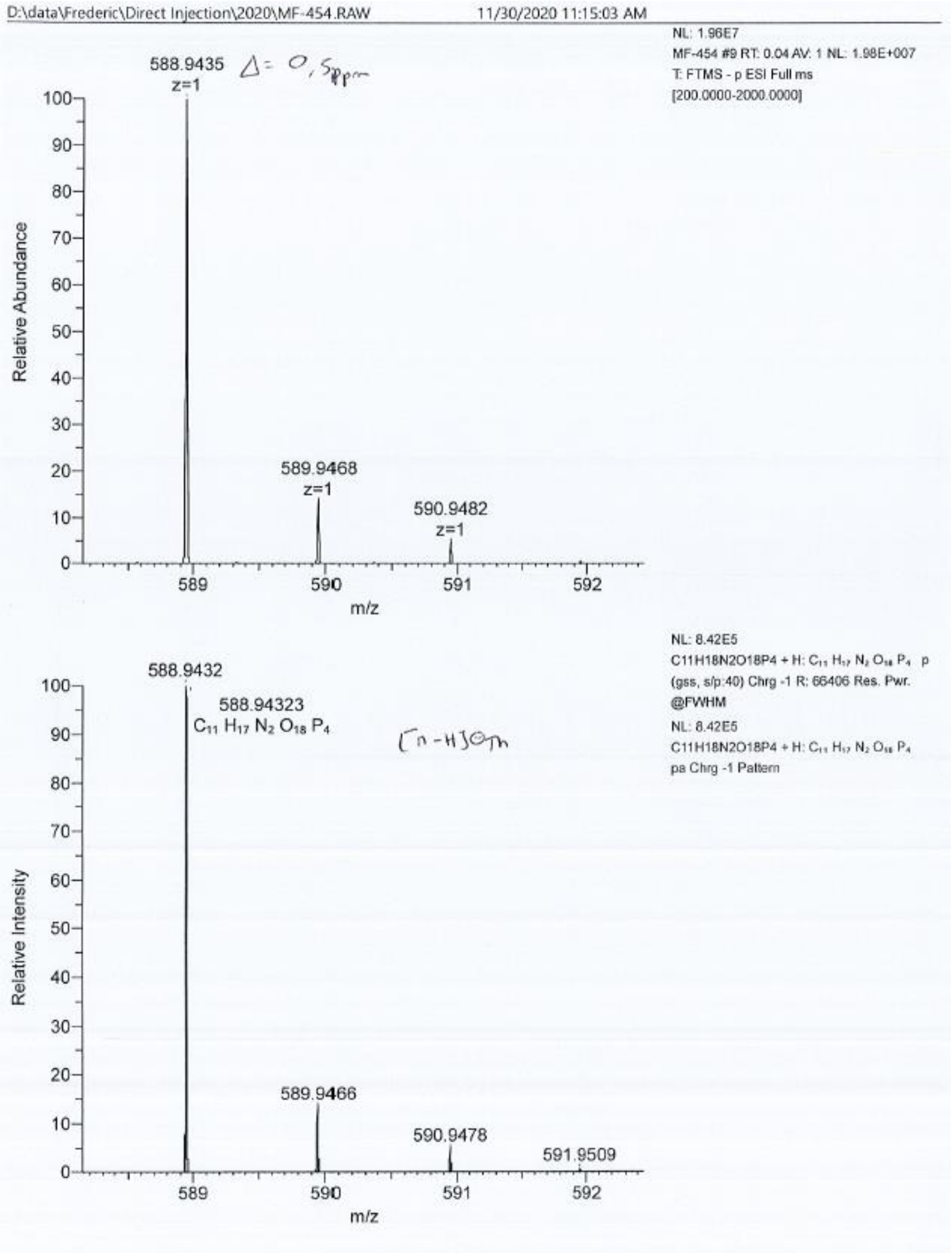

Supplementary Figure 69. HRMS analysis of compound **10**.

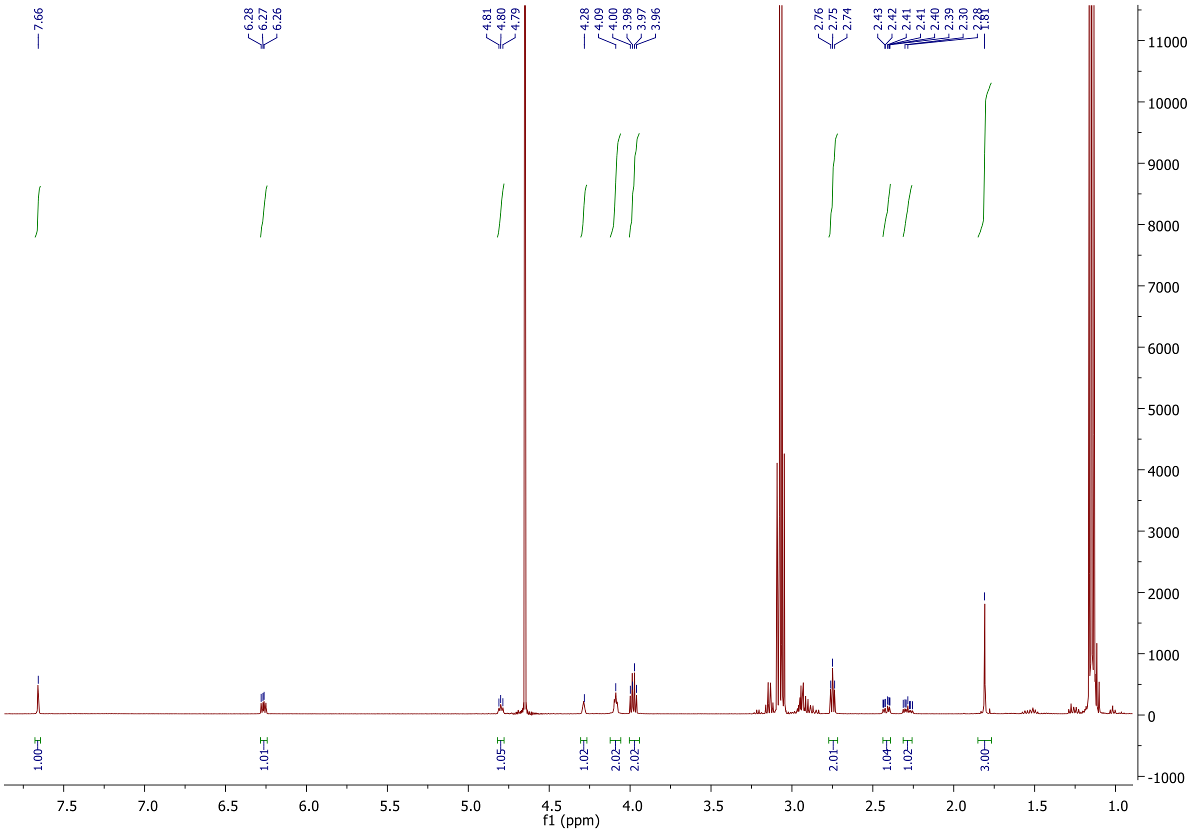

Supplementary Figure 70. ^{1}H NMR (400.13 MHz, D_2O) spectrum of compound **11**.

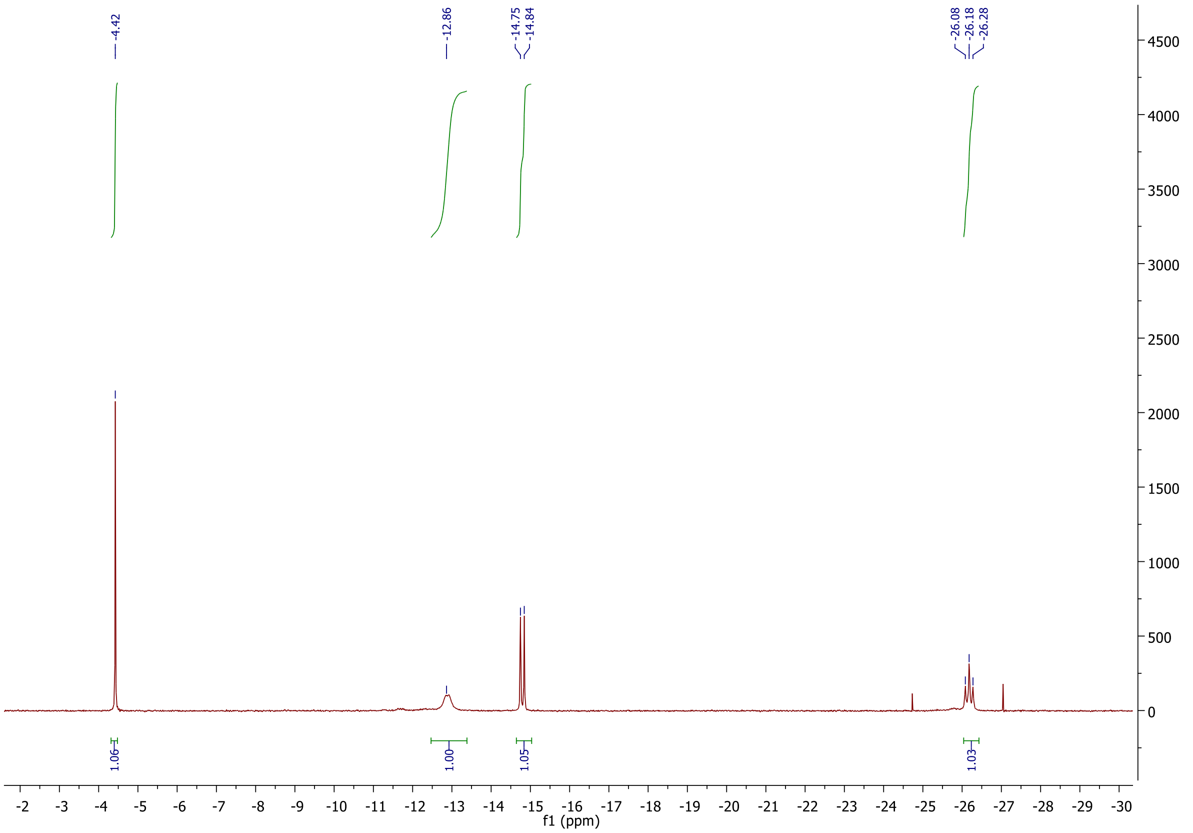

Supplementary Figure 71. ^{31}P NMR (161.62 MHz, D_2O) spectrum of compound **11**.

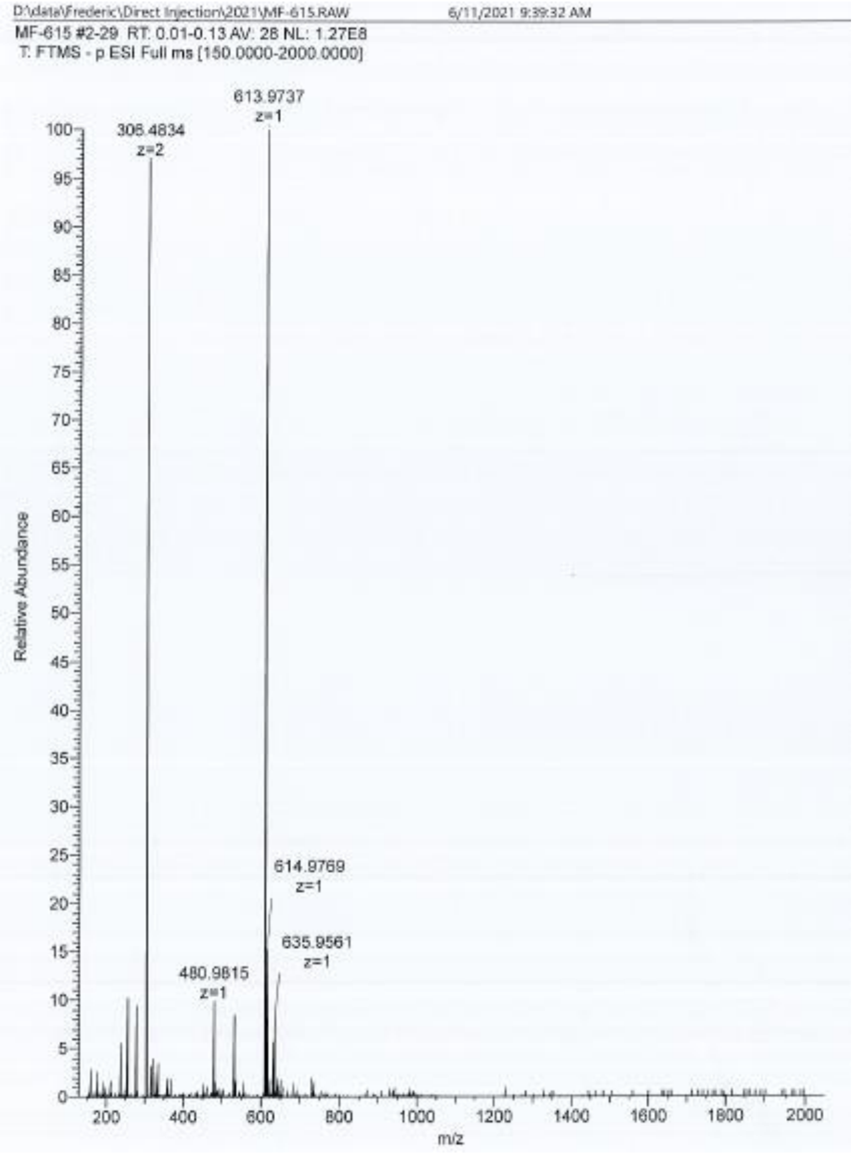
D:\data\Frederic\Direct Injection\2021\MF-615.RAW
6/11/2021 9:39:32 AM
MF-615 #2-29 RT: 0.01-0.13 AV: 28 NL: 1.27E8
T: FTMS - p ESI Full ms [150.0000-2000.0000]
613.9737
z=1
306.4834
z=2
614.9769
z=1
635.9561
z=1
480.9815
z=1
Relative Abundance
m/z

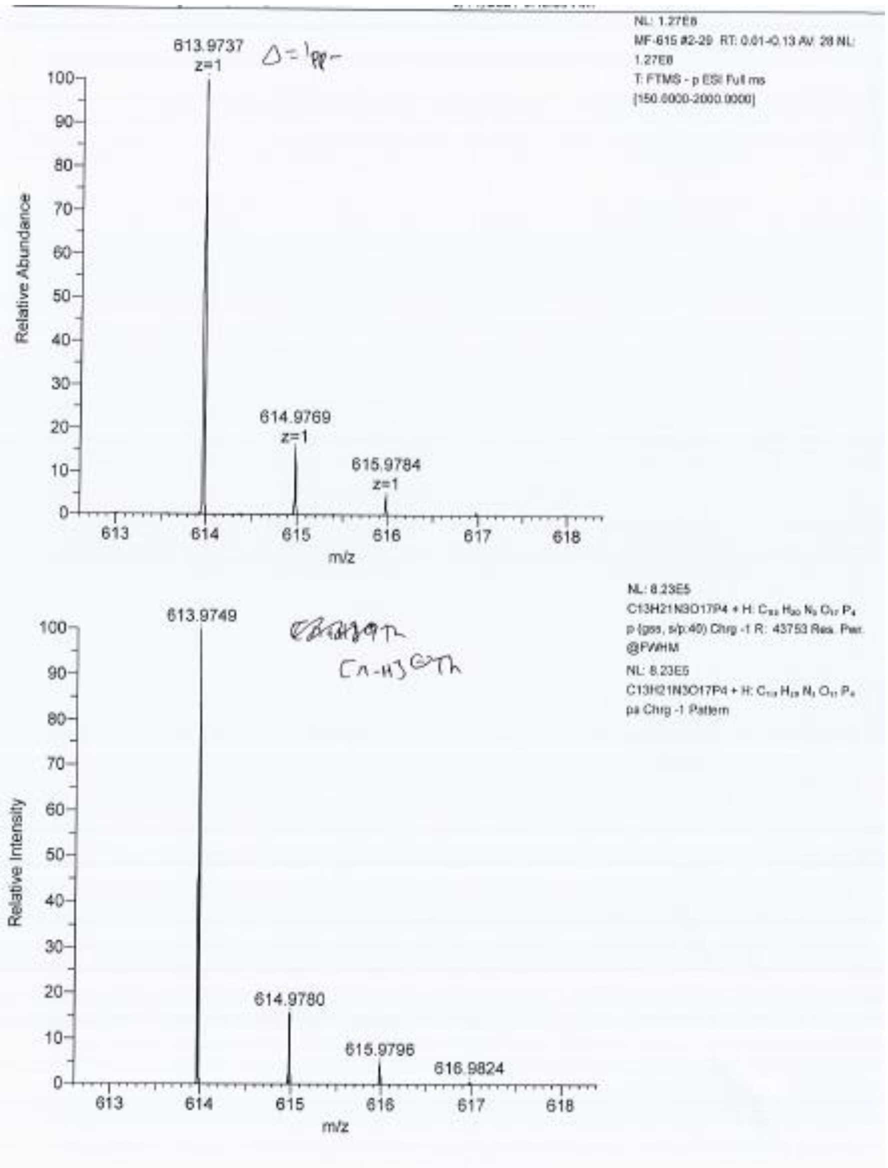

Supplementary Figure 72. HRMS analysis of compound **11**.

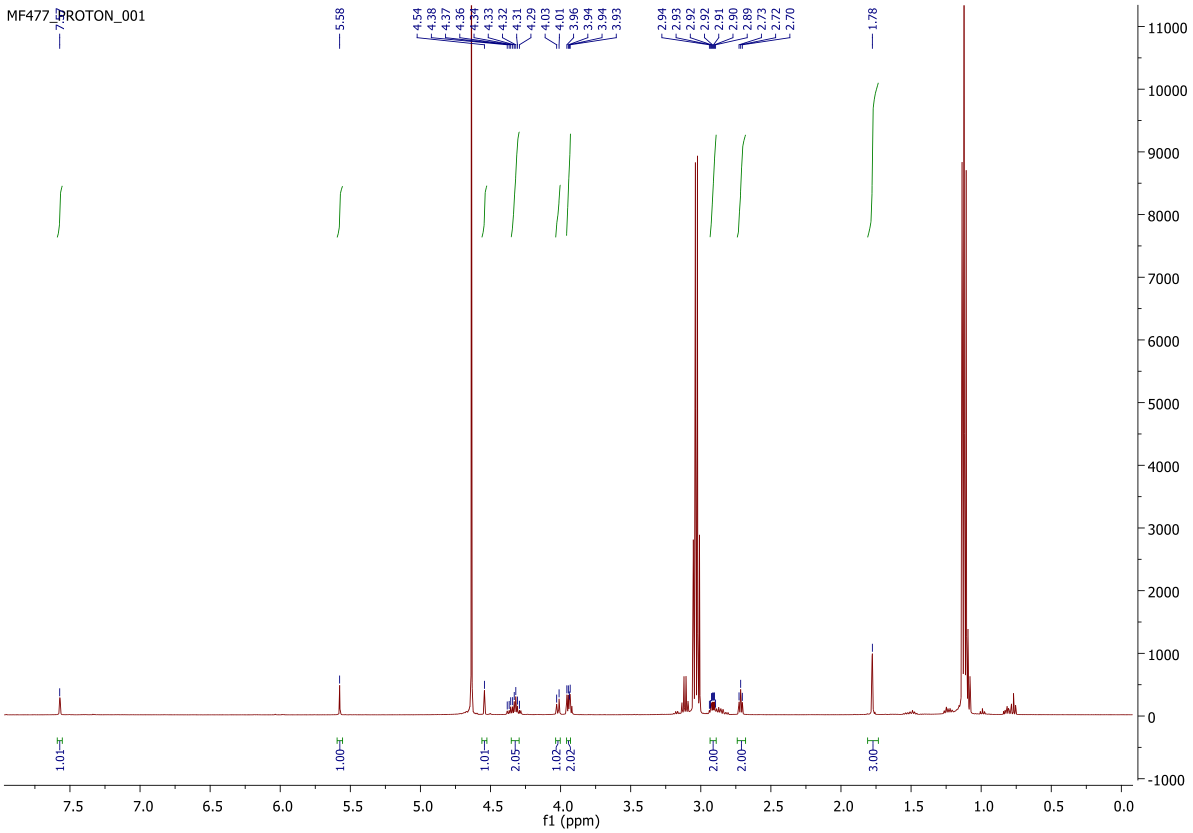

Supplementary Figure 73. ^{1}H NMR (400.13 MHz, D_2O) spectrum of compound **12**.

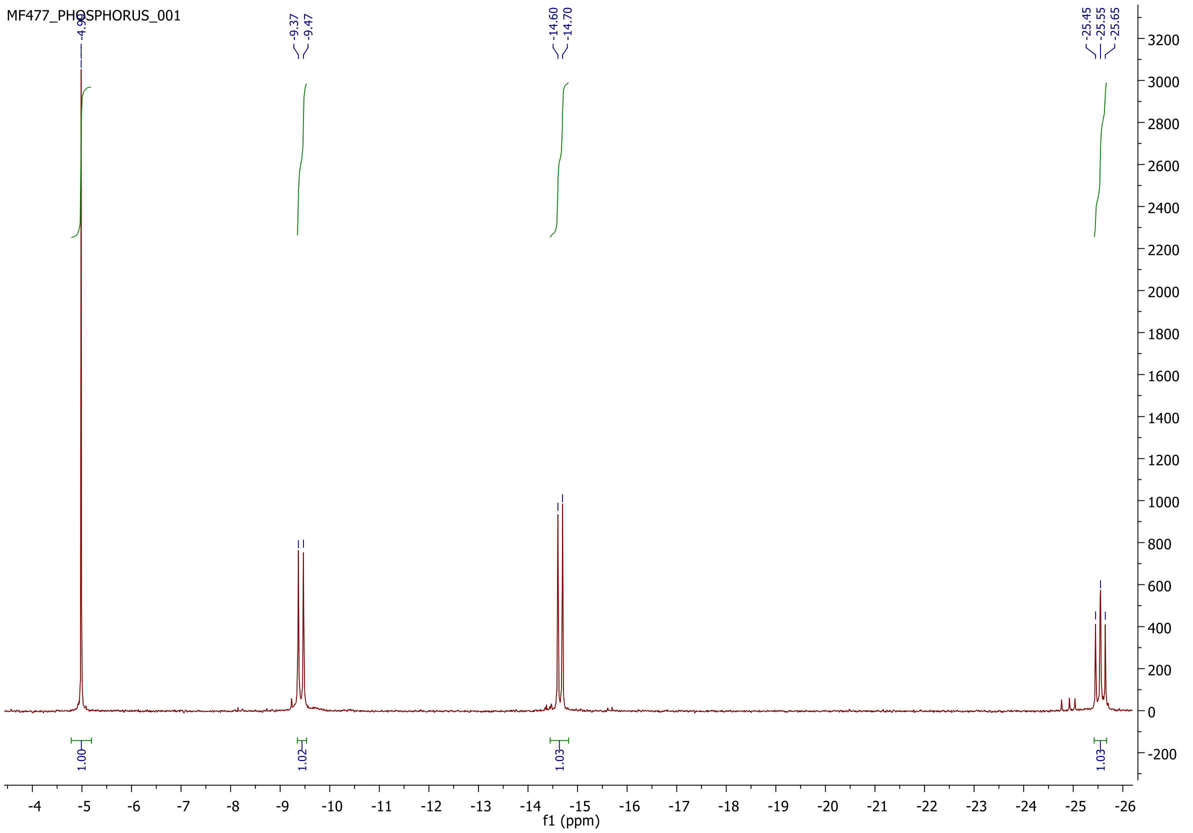

Supplementary Figure 74. ^{31}P NMR (161.62 MHz, D_2O) spectrum of compound **12**.

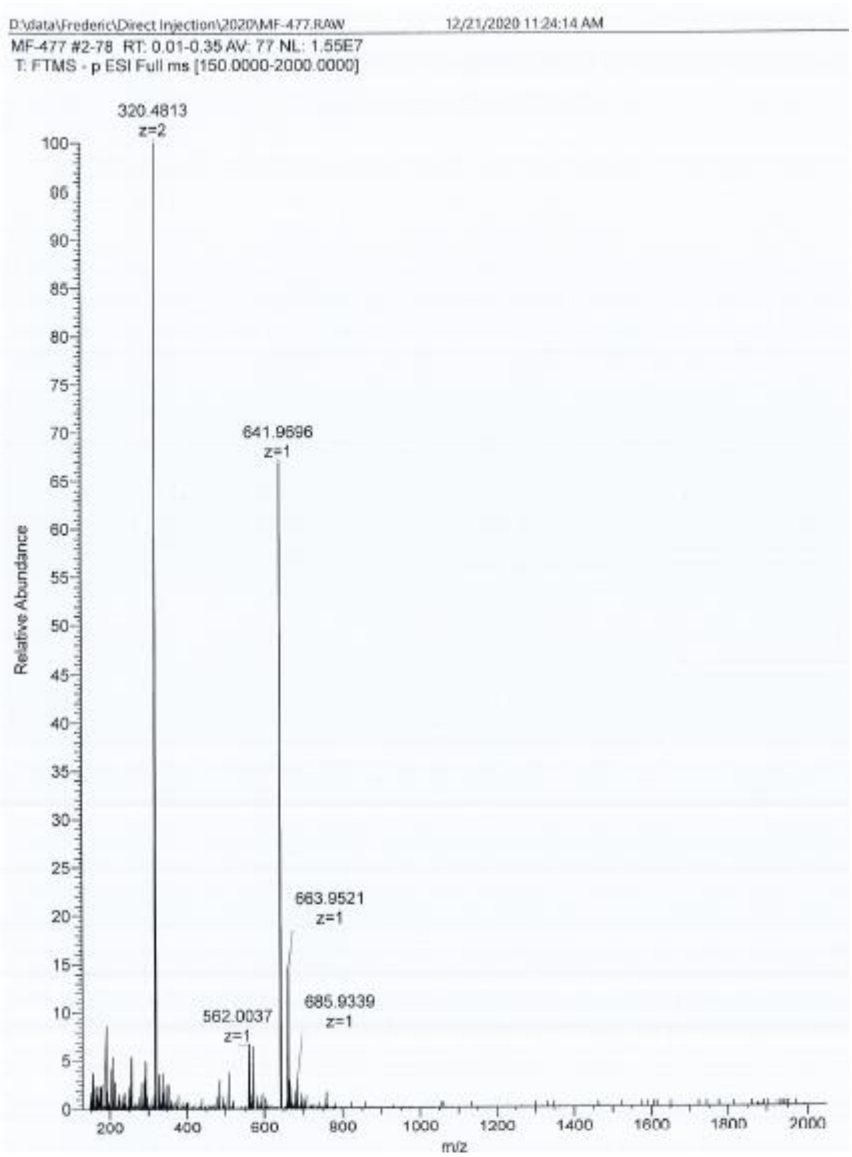
D:\data\Frederic\Direct Injection\2020\MF-477.RAW
12/21/2020 11:24:14 AM
MF-477 #2-78 RT: 0.01-0.35 AV: 77 NL: 1.55E7
T: FTMS - p ESI Full ms [150.0000-2000.0000]
320.4813
z=2
641.9696
z=1
663.9521
z=1
562.0037
z=1
685.9339
z=1
Relative Abundance
m/z

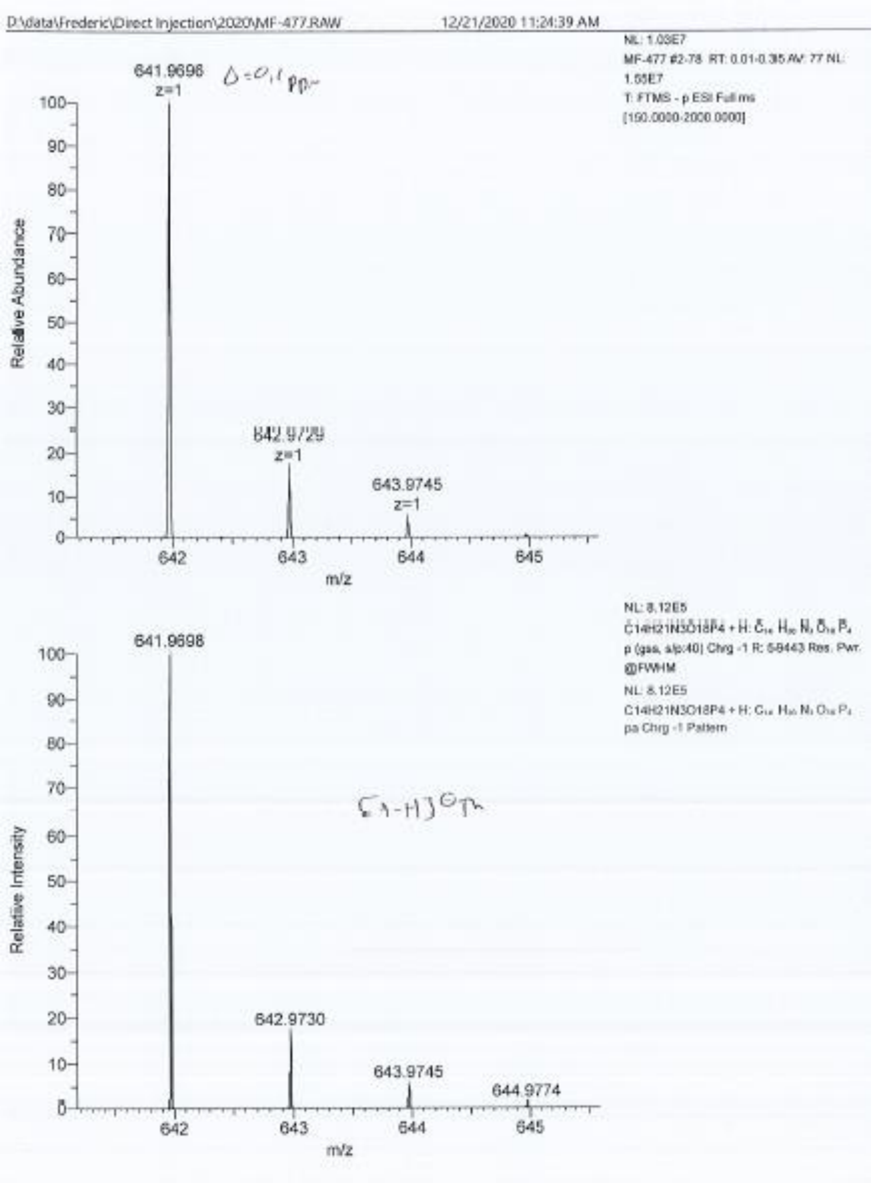

Supplementary Figure 75. HRMS analysis of compound **12**.

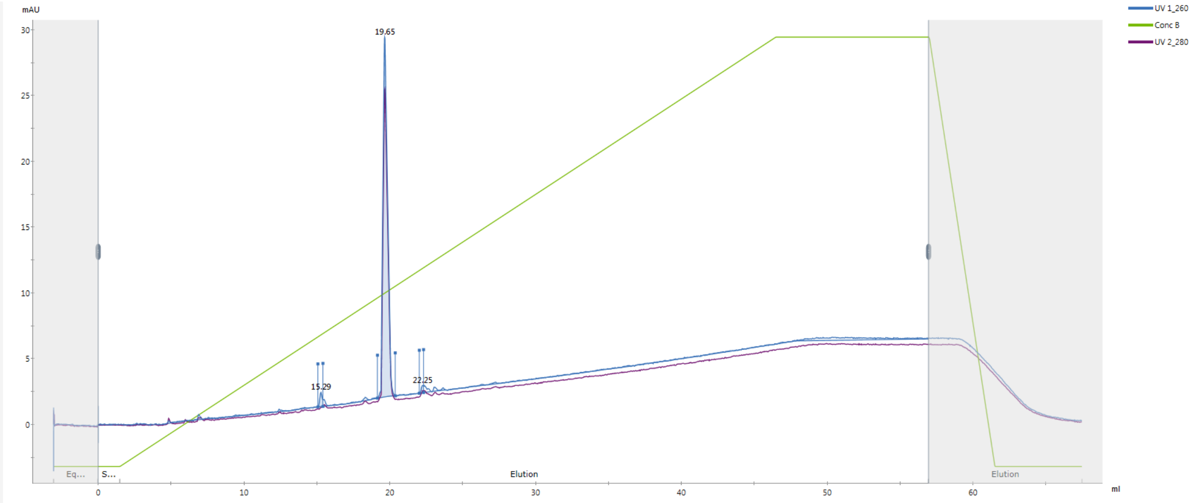

Supplementary Figure 76. HPLC (anion exchange) chromatogram of purified compound **12**.

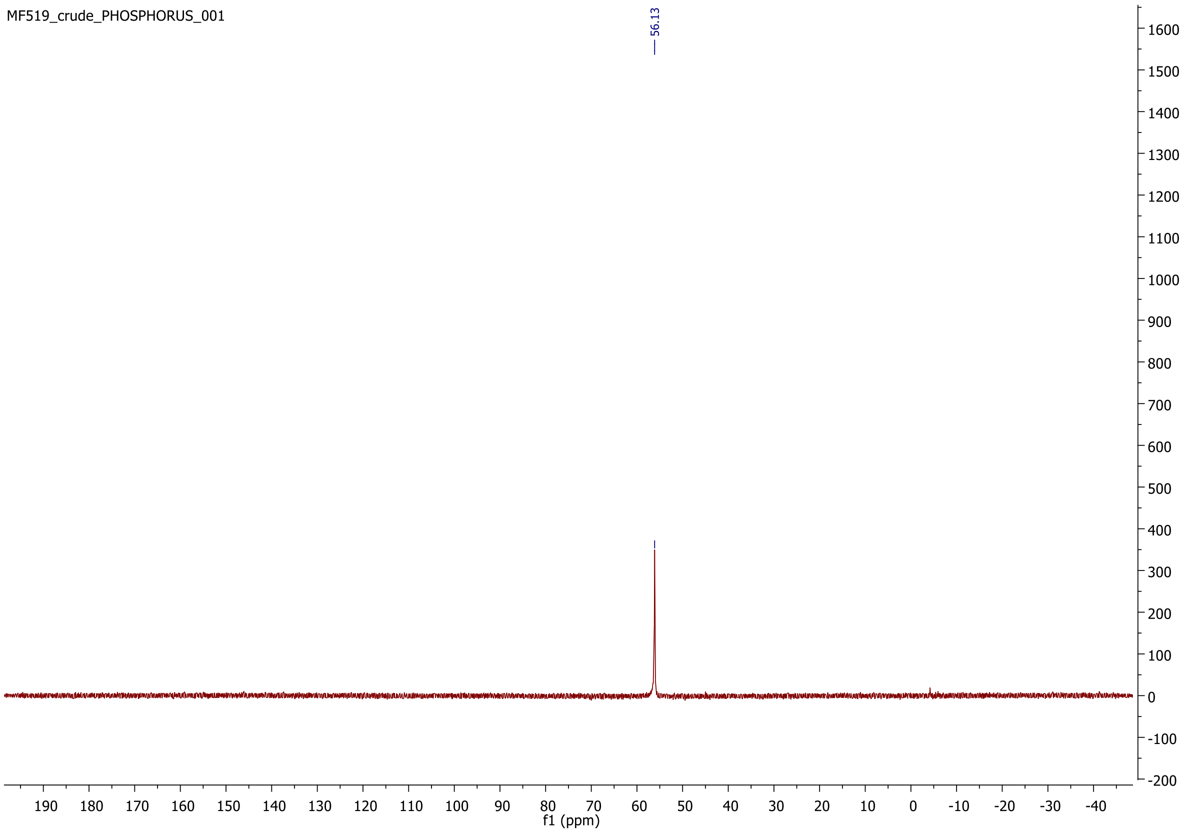

Supplementary Figure 77. ^{31}P NMR (161.62 MHz, $CDCl_3$) spectrum of compound **13**.

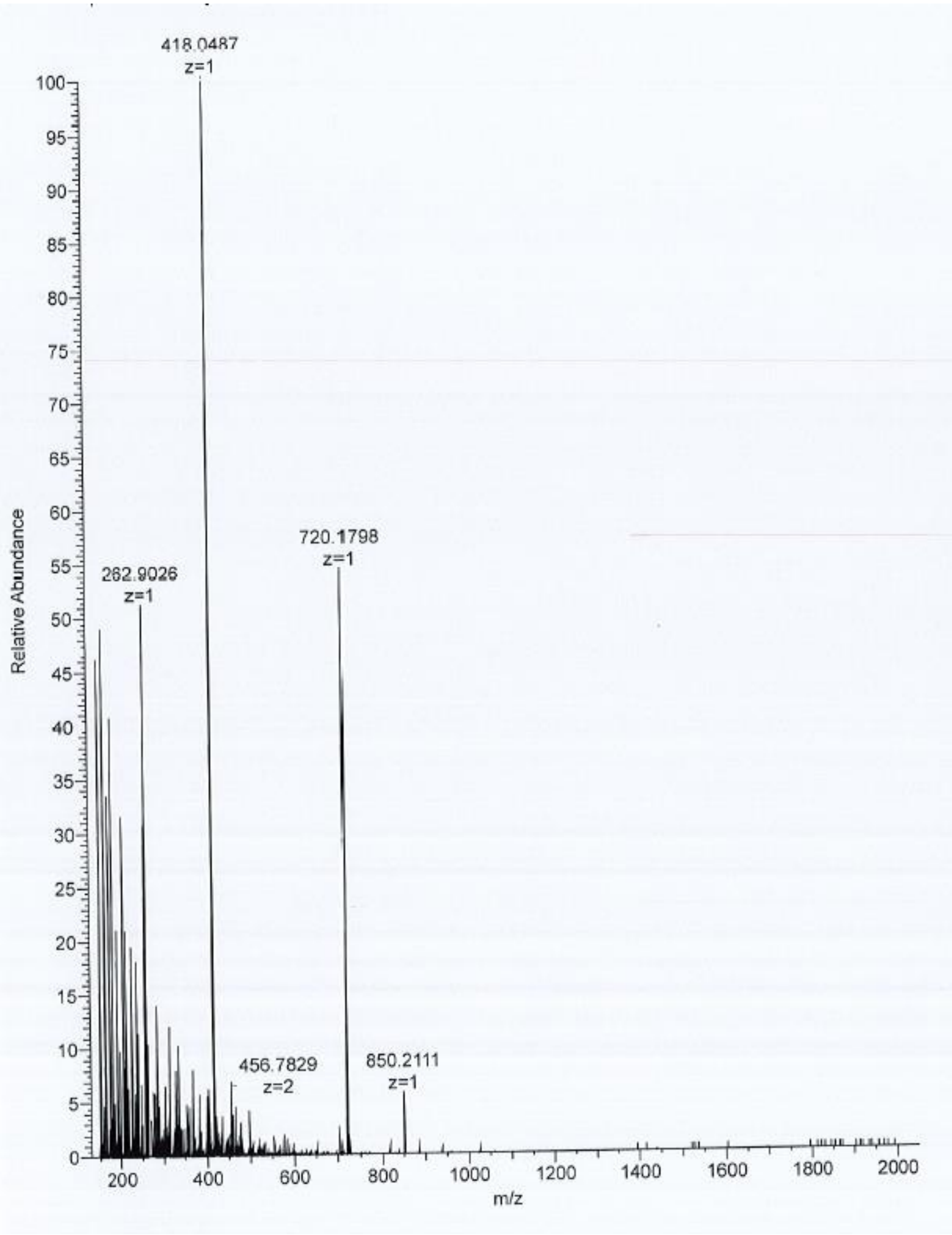

418.0487
z=1
720.1798
z=1
262.9026
z=1
456.7829
z=2
850.2111
z=1
Relative Abundance
m/z

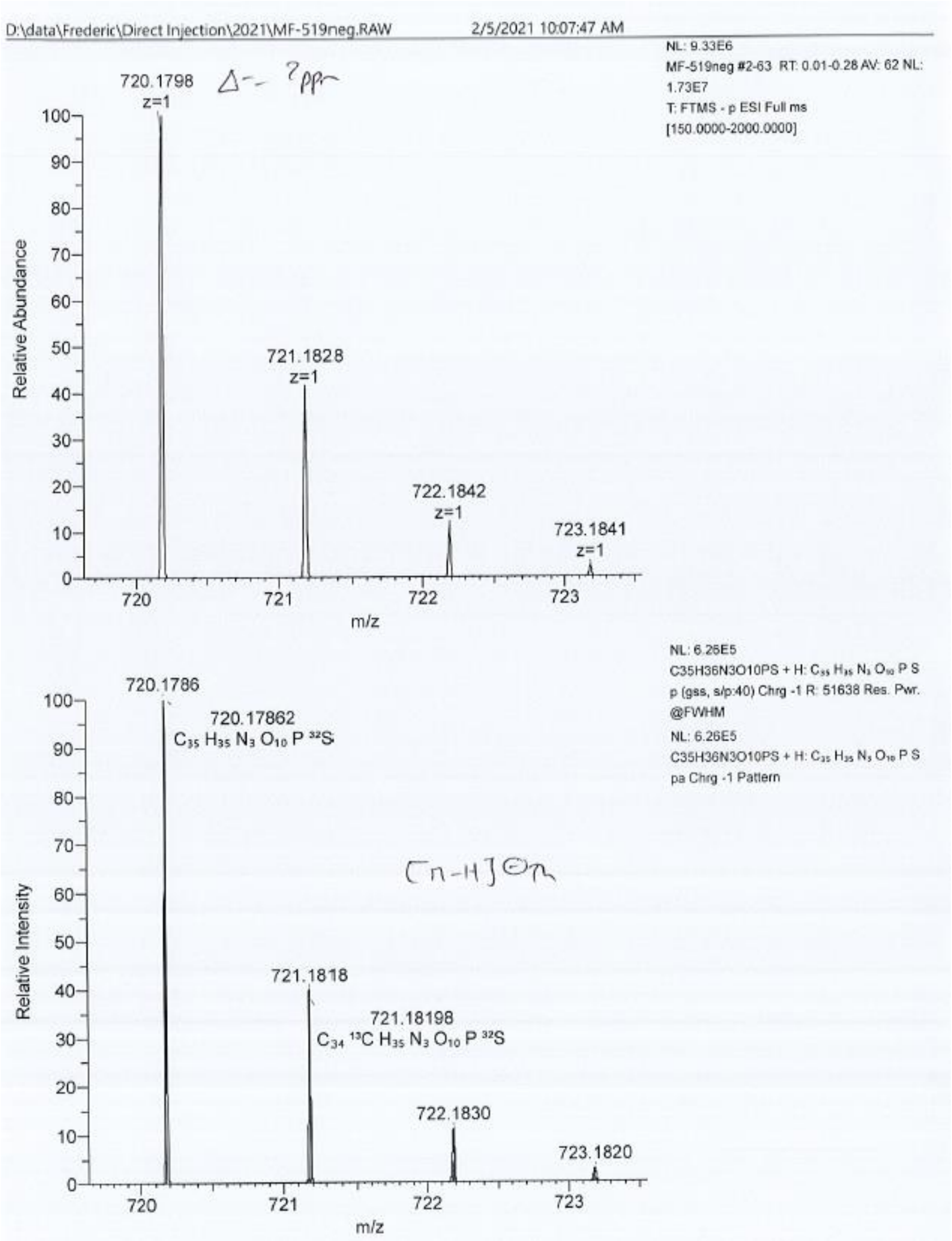

Supplementary Figure 78. HRMS analysis of compound **13**.

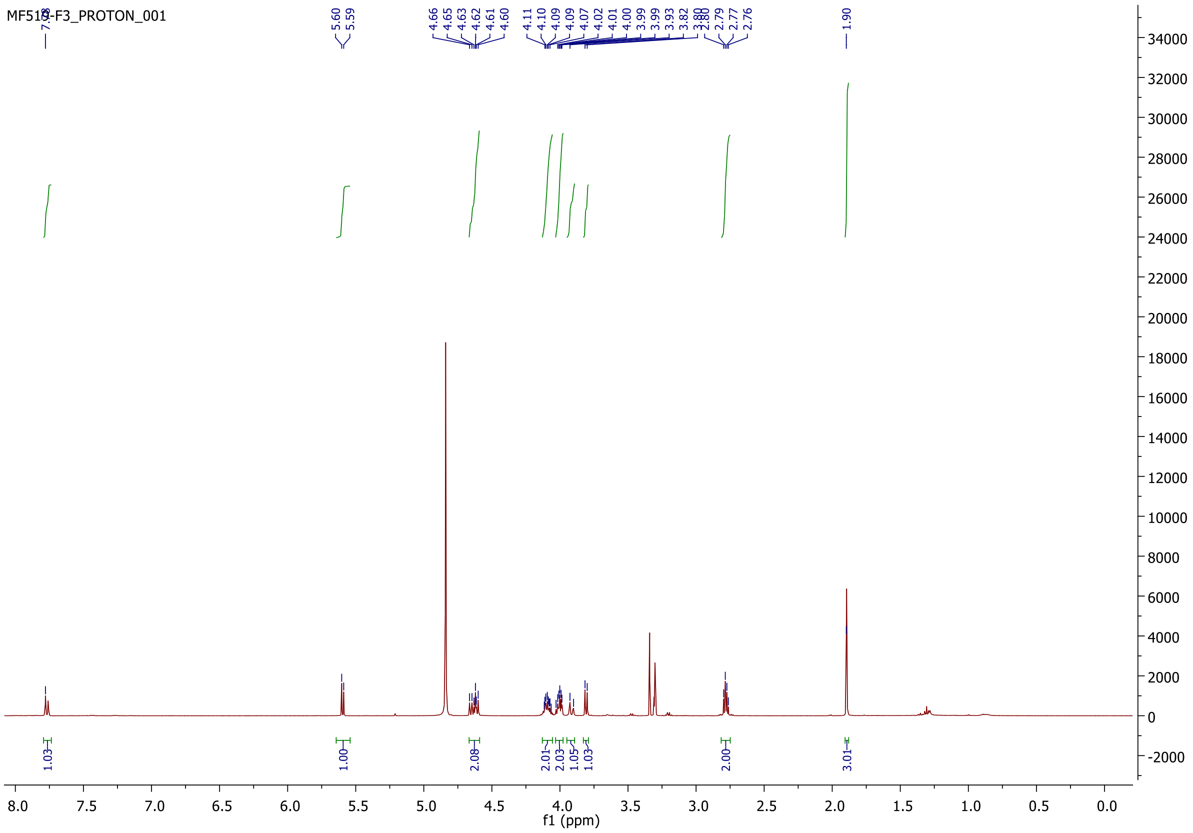

Supplementary Figure 79. ^{1}H NMR (500.13 MHz, MeOD) spectrum of compound **14**.

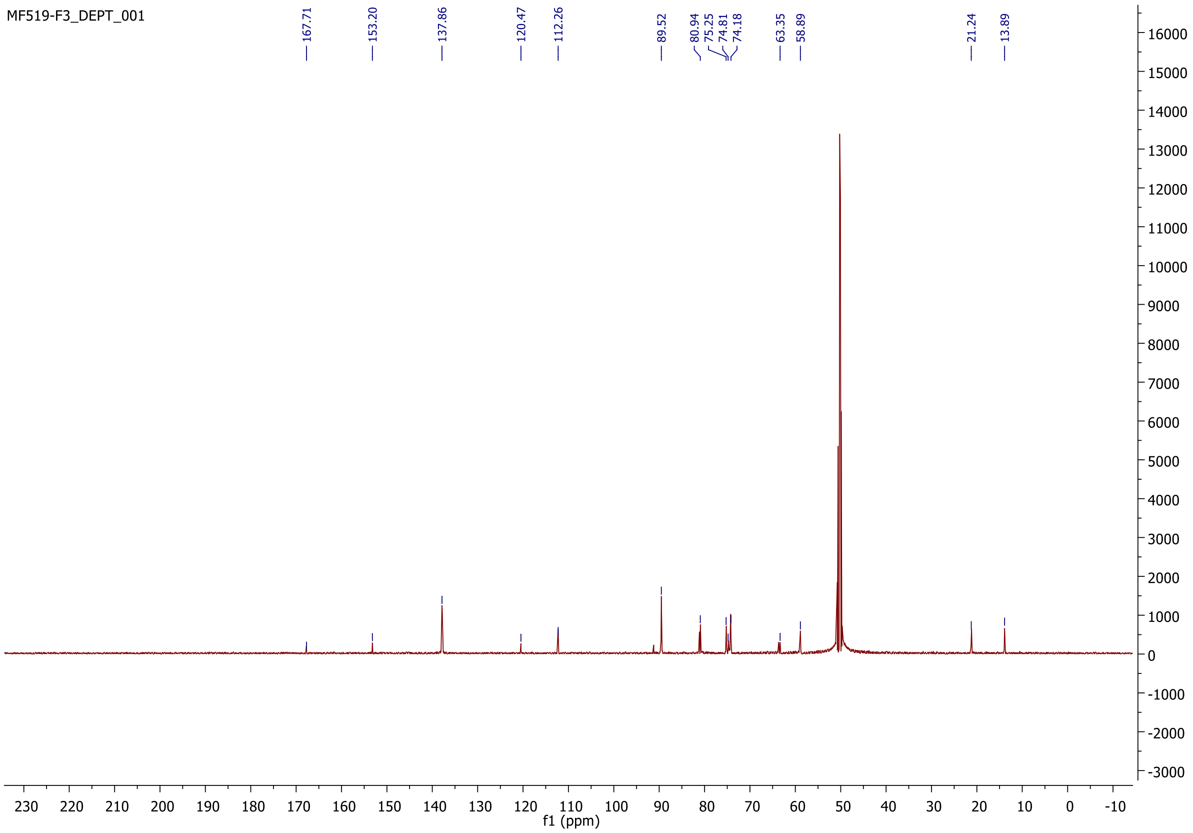

Supplementary Figure 80. ^{13}C NMR (100.62 MHz, MeOD) spectrum of compound **14**.

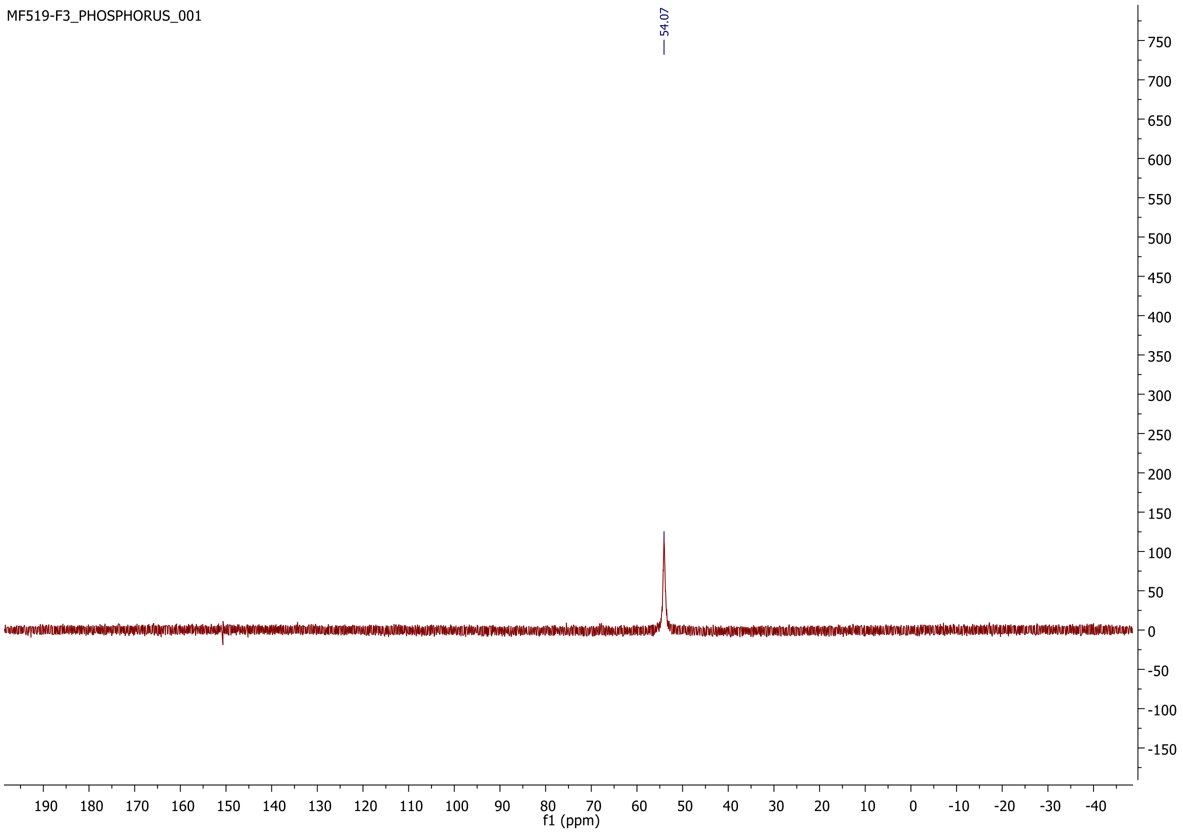

Supplementary Figure 81. ^{31}P NMR (161.62 MHz, MeOD) of compound **14**.

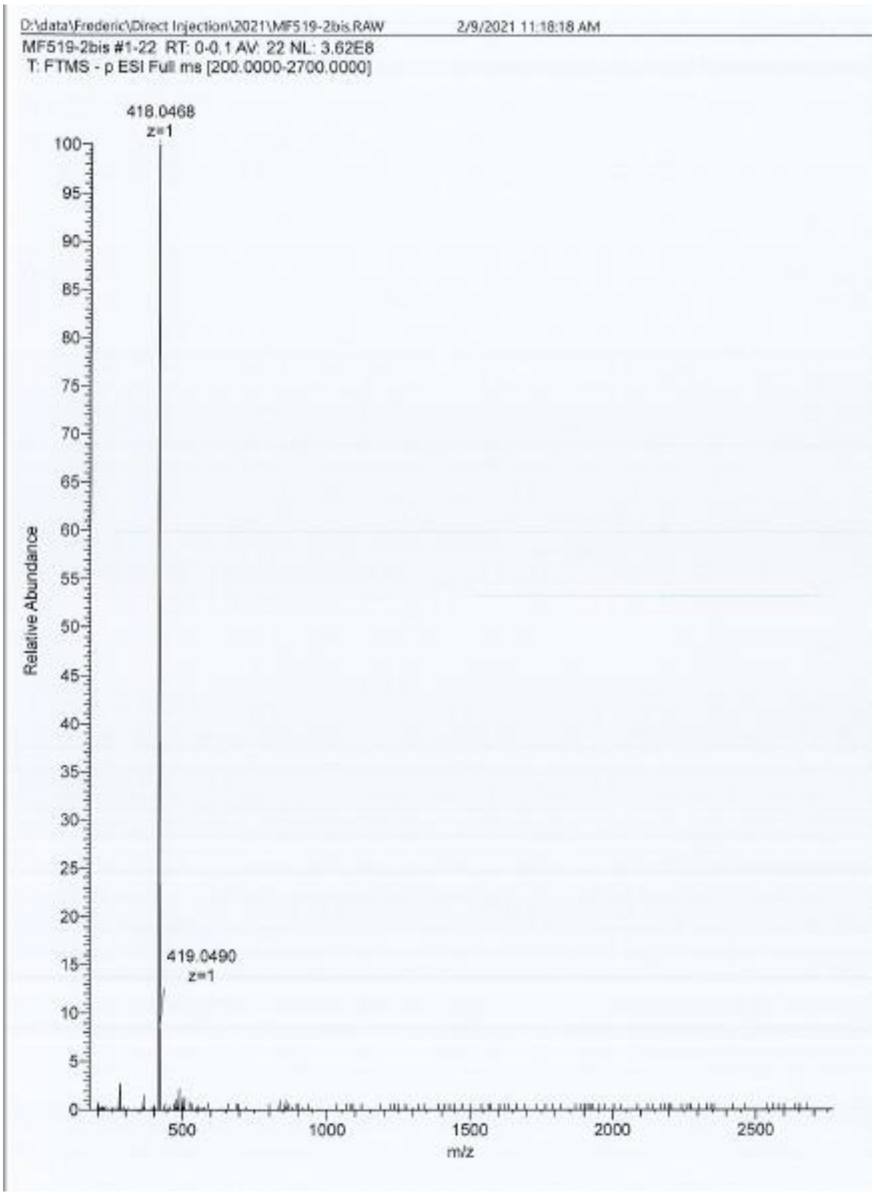
D:\data\Frederic\Direct Injection\2021\MF519-2bis.RAW
2/9/2021 11:18:18 AM
MF519-2bis #1-22 RT: 0-0.1 AV: 22 NL: 3.62E8
T: FTMS - p ESI Full ms [200.0000-2700.0000]
418.0468
z=1
419.0490
z=1
Relative Abundance
m/z

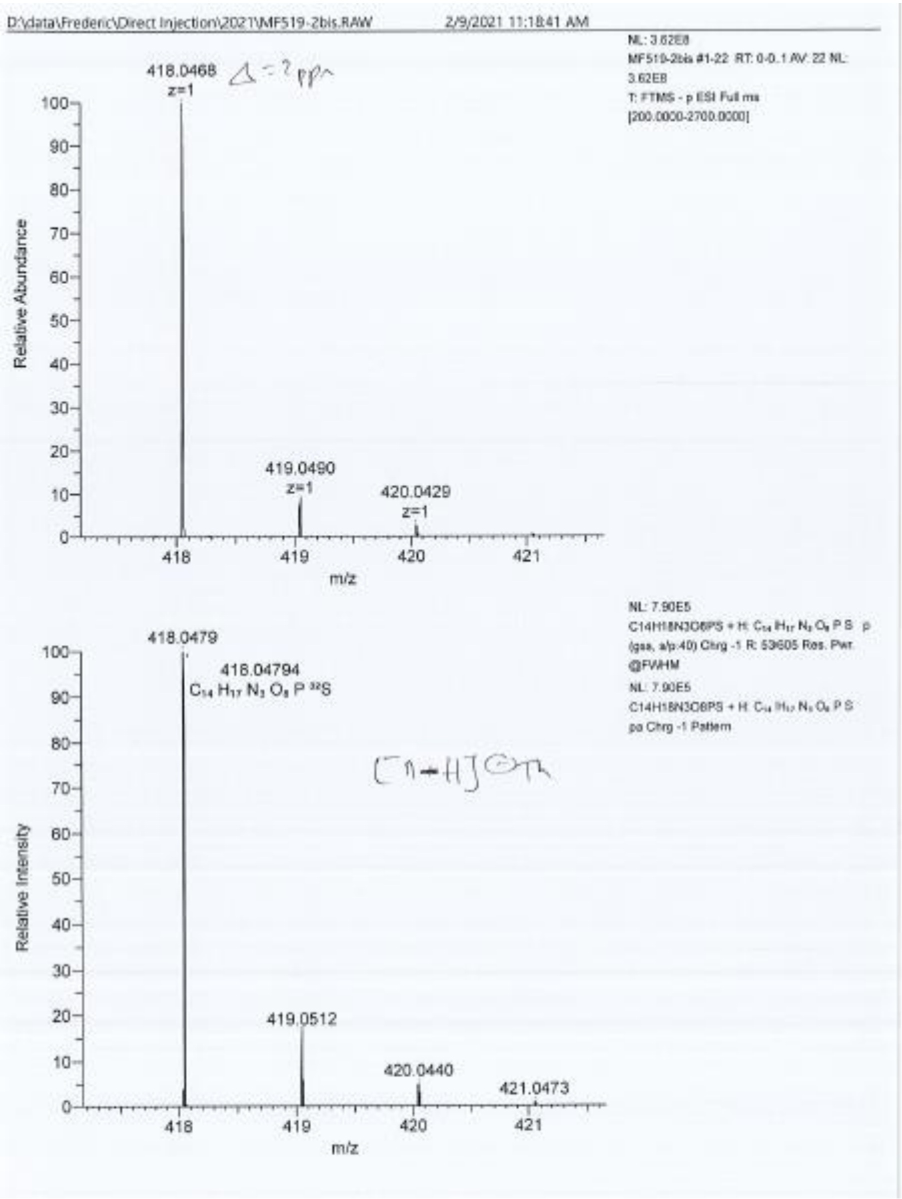

Supplementary Figure 82. HRMS analysis of compound **14**.

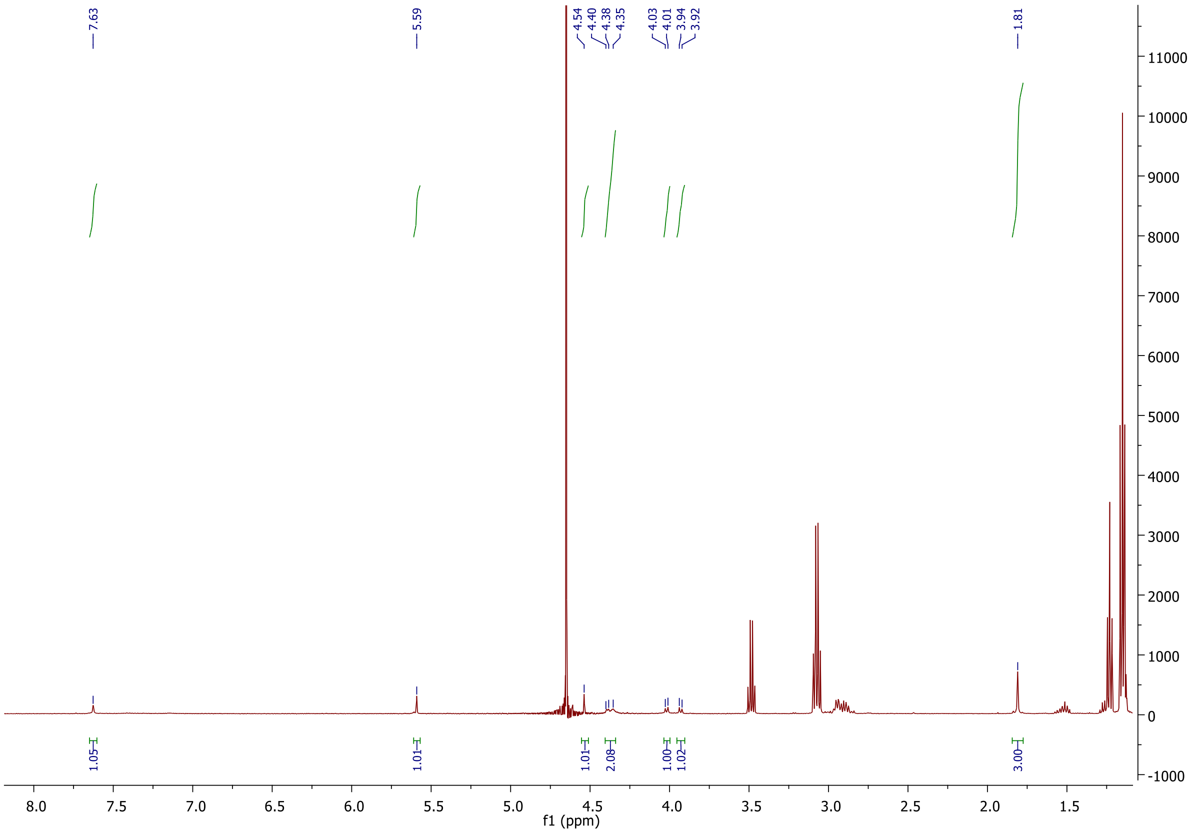

Supplementary Figure 83. ^{1}H NMR (500.13 MHz, D_2O) spectrum of compound **15**.

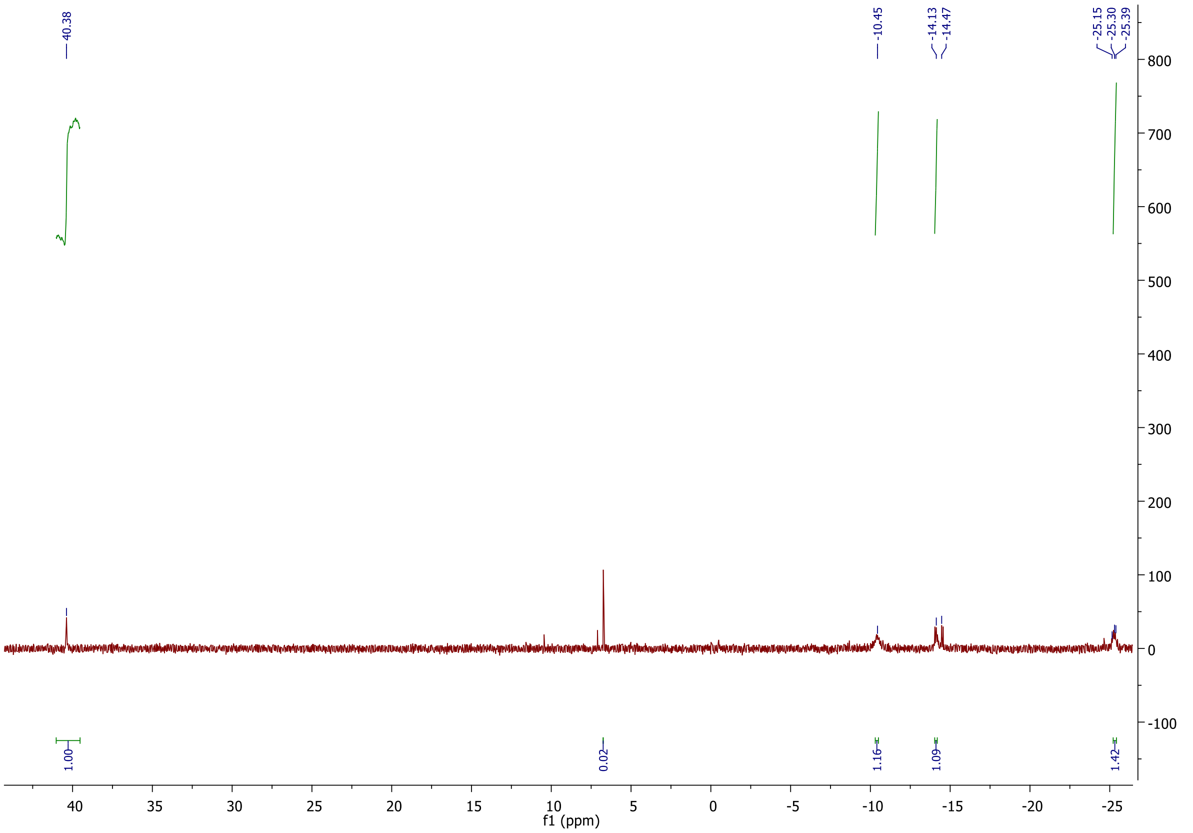

Supplementary Figure 84. ^{31}P NMR (500.13 MHz, D_2O) spectrum of compound **15**.

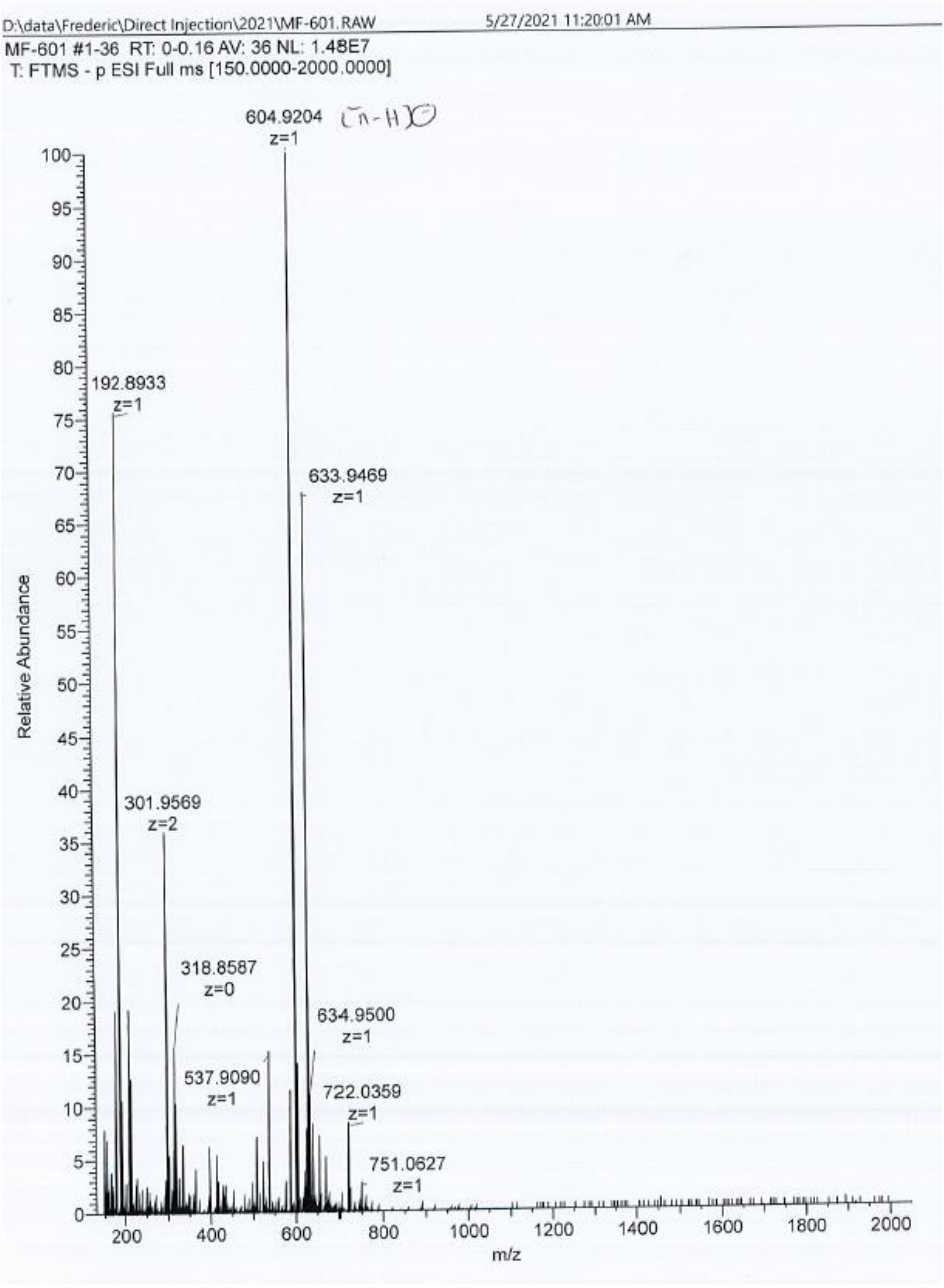
D:\data\Frederic\Direct Injection\2021\MF-601.RAW
5/27/2021 11:20:01 AM
MF-601 #1-36 RT: 0-0.16 AV: 36 NL: 1.48E7
T: FTMS - p ESI Full ms [150.0000-2000.0000]
604.9204 z=1
192.8933 z=1
633.9469 z=1
301.9569 z=2
318.8587 z=0
634.9500 z=1
537.9090 z=1
722.0359 z=1
751.0627 z=1
Relative Abundance
m/z

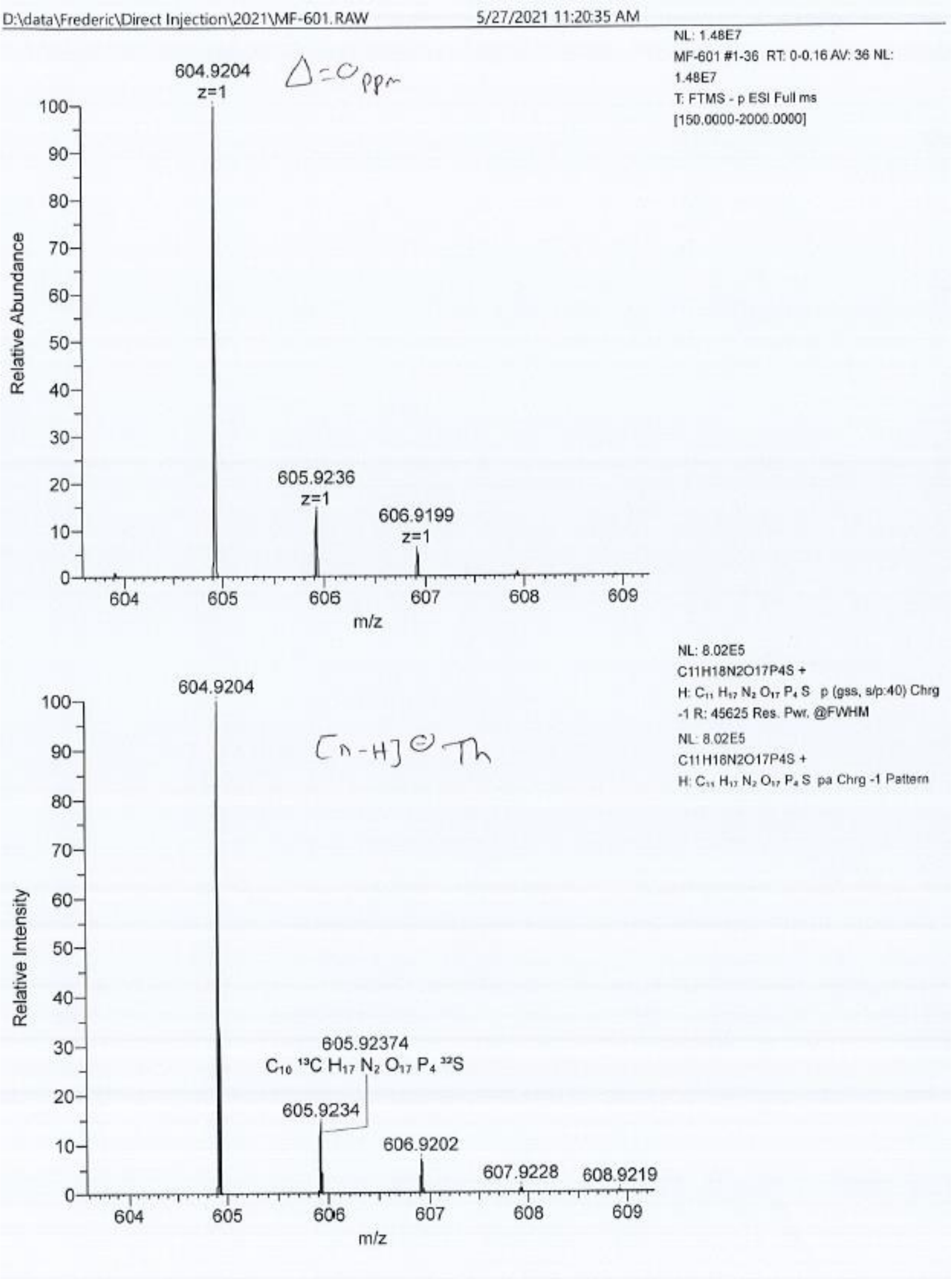

Supplementary Figure 85. HRMS analysis of compound **15**.

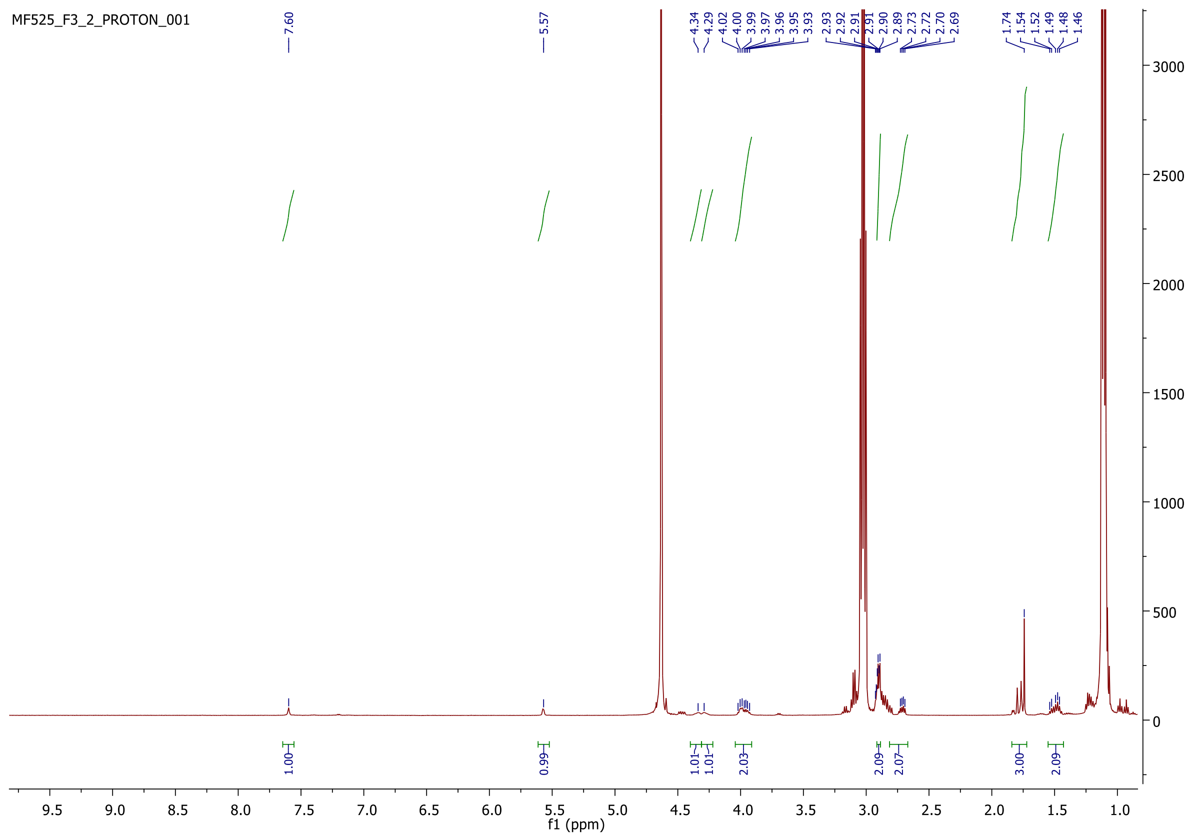

Supplementary Figure 86. ^{1}H NMR (500.13 MHz, D_2O) spectrum of compound **16**.

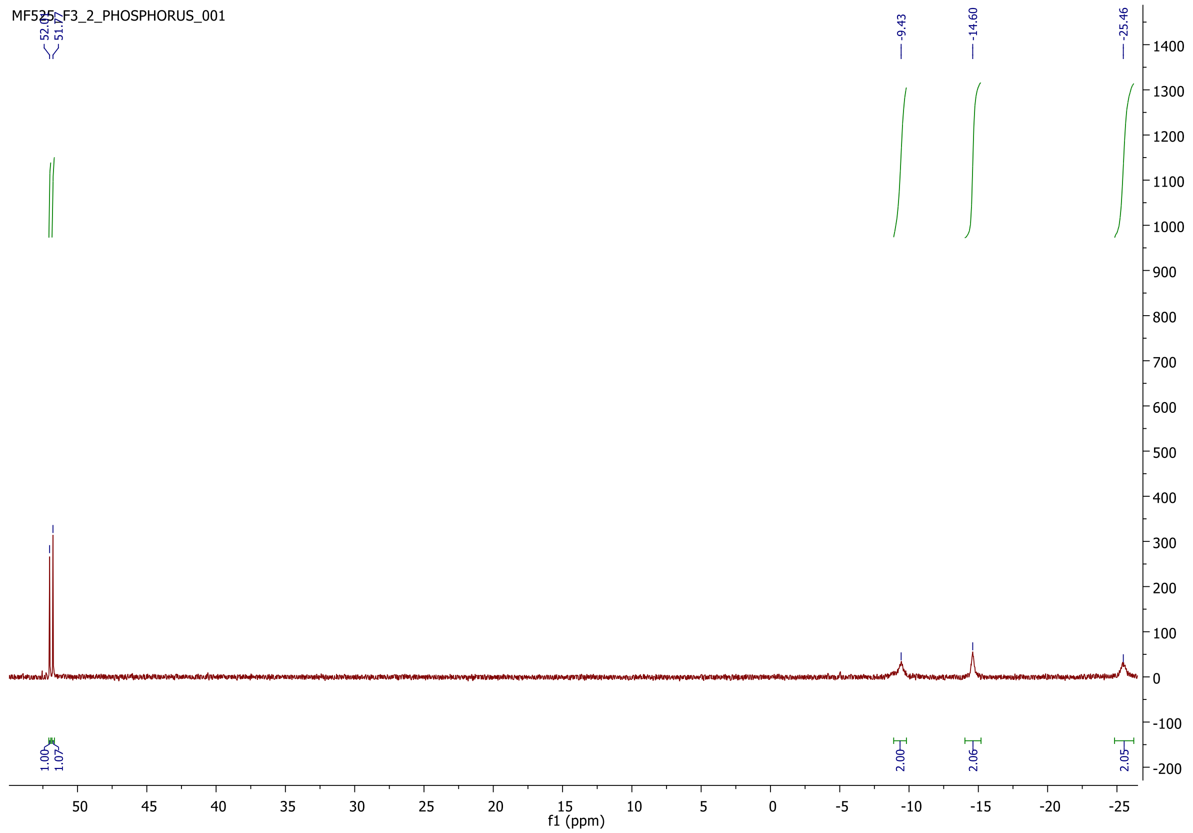

Supplementary Figure 87. ^{31}P NMR (161.62 MHz, D_2O) spectrum of compound **16**.

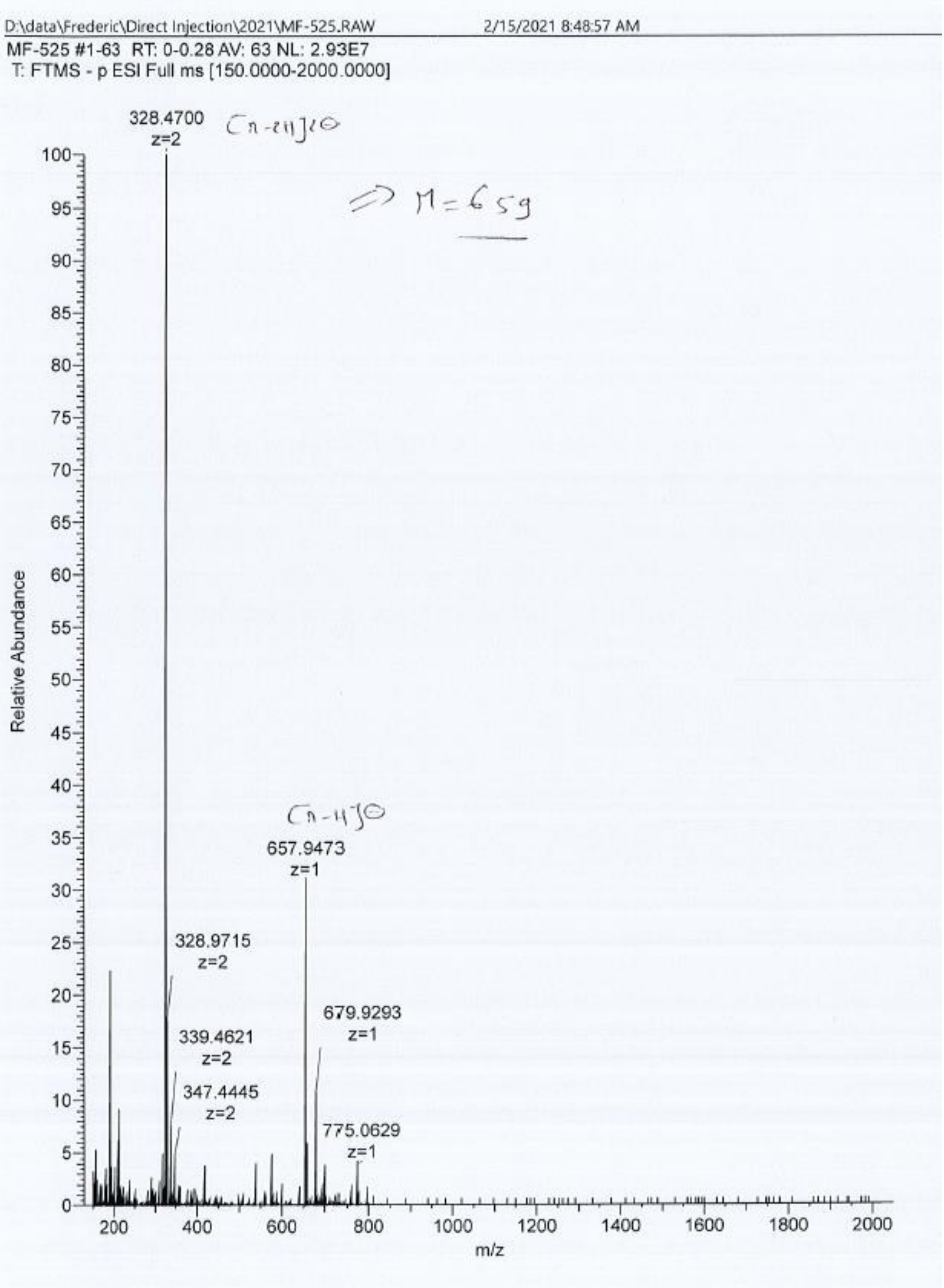
D:\data\Frederic\Direct Injection\2021\MF-525.RAW
2/15/2021 8:48:57 AM
MF-525 #1-63 RT: 0-0.28 AV: 63 NL: 2.93E7
T: FTMS - p ESI Full ms [150.0000-2000.0000]
328.4700
z=2
657.9473
z=1
328.9715
z=2
679.9293
z=1
339.4621
z=2
347.4445
z=2
775.0629
z=1
Relative Abundance
m/z

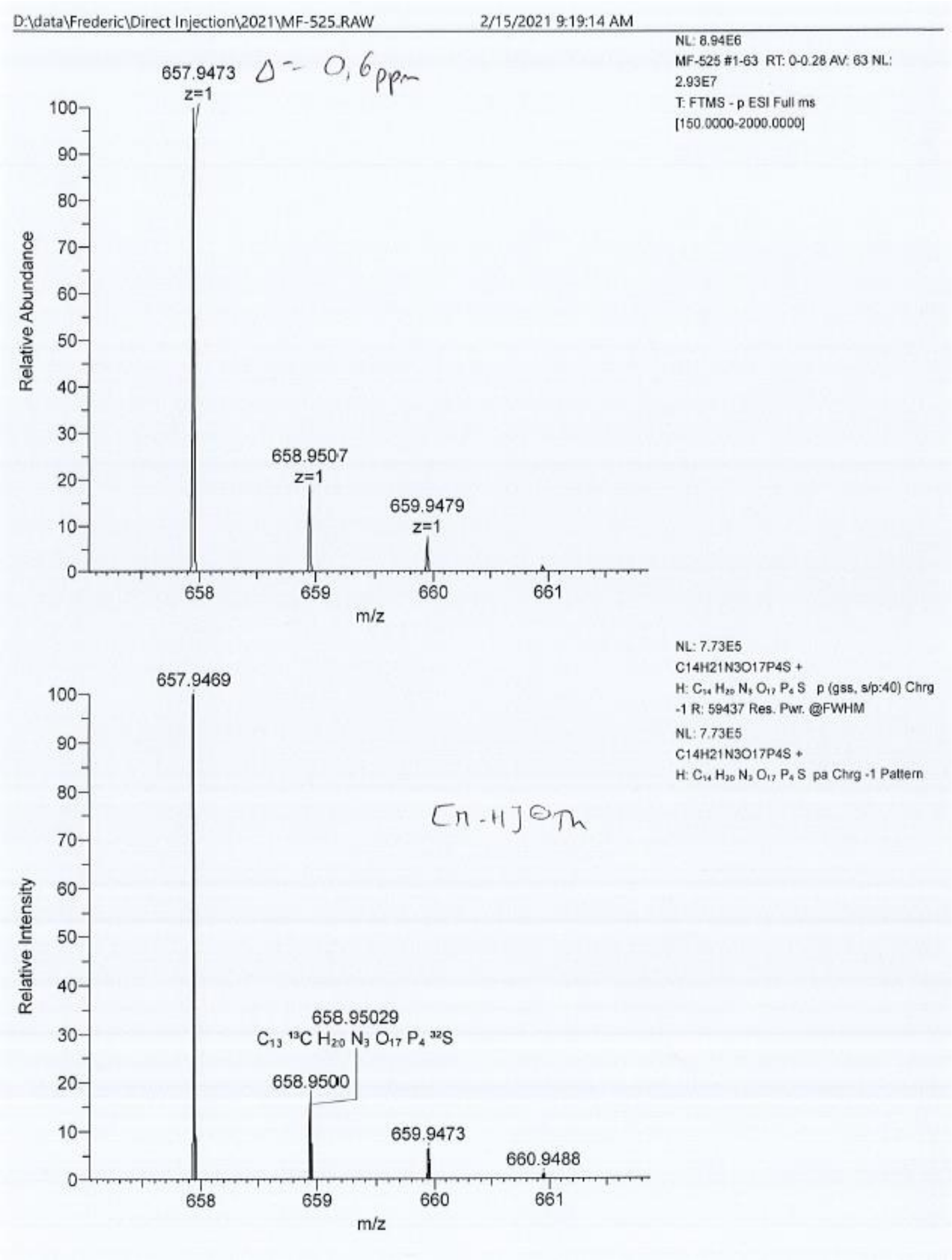

Supplementary Figure 88. HRMS analysis of compound **16**.

6. MS analysis of Klenow polymerase

Supplementary Note 2:

- 1 µL of Klenow fragment (5000 units/mL) was diluted in 59 µL of 0.15% formic acid (~ 0.083 units/µL).
- 50 µL (i.e., ~4.2 units) was loaded onto an ACQUITY UPLC BEH C4 Trap column (2.1 µm x 5 mm) equilibrated at room temperature in 5% acetonitrile, 0.15% formic acid and desalted for 2 min at 100 µL/min with 0.15 % formic acid. The protein was directly eluted into the mass spectrometer with a linear gradient of acetonitrile in 0.15% formic acid (from 5 to 90 % in two minutes at 60 µL/min).
- MS spectra were acquired on a Synapt G2-Si HDMS mass spectrometer in positive and resolution mode (400-2000 *m/z*). To ensure mass accuracy, a Glu-1-Fibrinopeptide B solution (200 fmol/µL in 50% acetonitrile, 0.1% formic acid) was continuously infused through the reference probe of the electrospray source.
- A protein control (Bet v 1; 10 pmoles on column, Expected average Mw = 17439.4315 Da) was injected to evaluate the performance of the instrument.

Results:

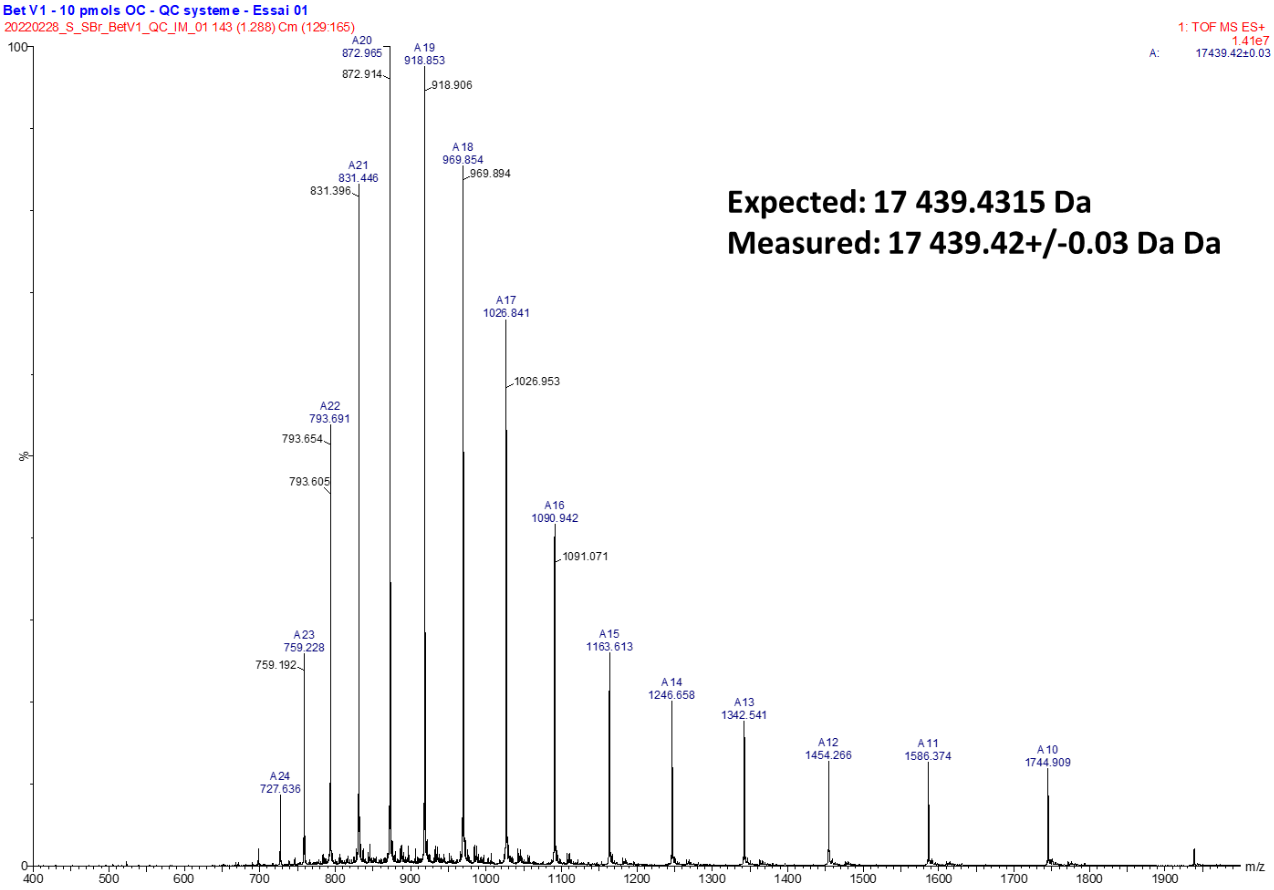

Supplementary Figure 89. MS analysis of protein control (Bet v 1).

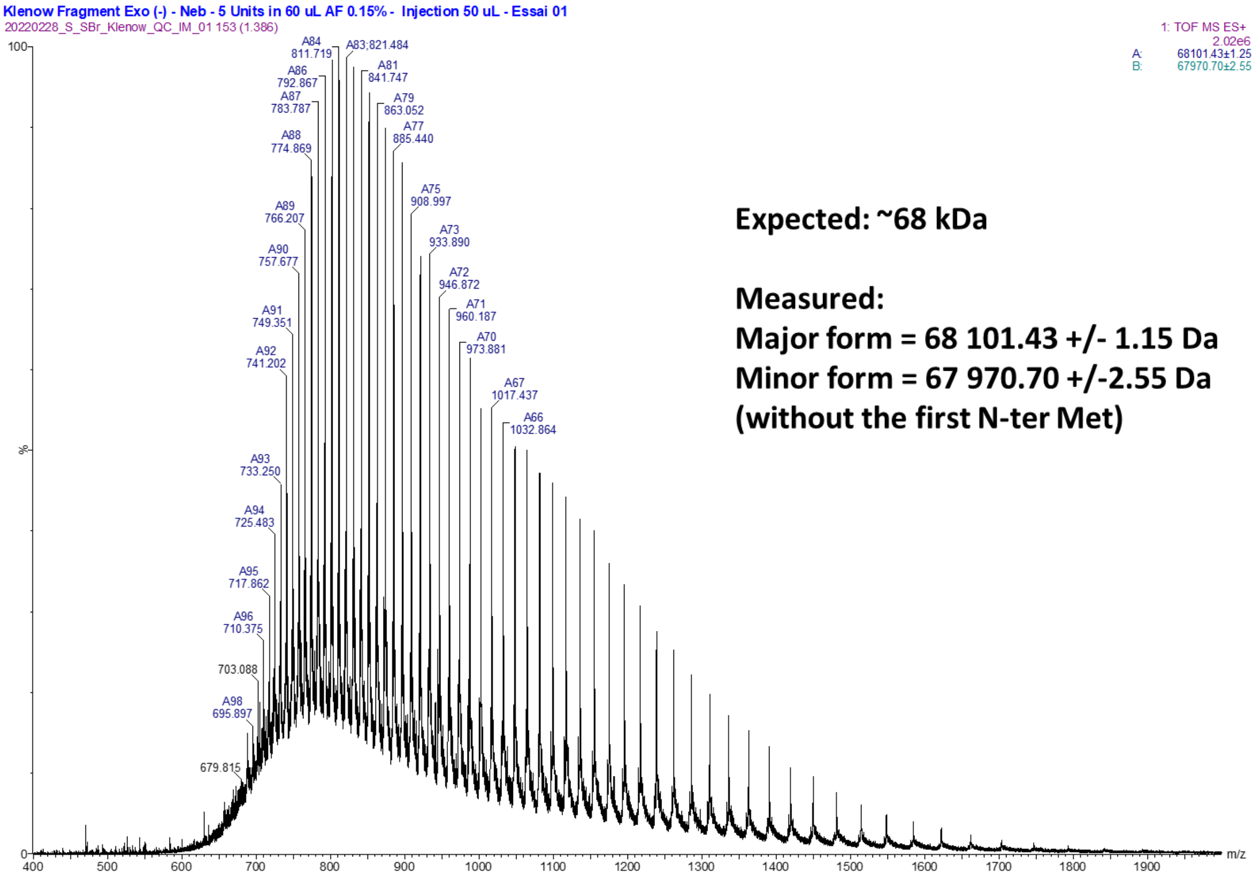

Supplementary Figure 90. MS analysis of Kf (*exo*⁻).

Supplementary Table 1. Summary of results of HDX-MS analysis.

Protein	Expected average MW (Da)	Measured average MW (Da)	Comments
Bet v 1	17 439.4315	**17 439.42 +/- 0.03 Da**	Δm = -0.0115 Da (-0.66 ppm)
Klenow fragment exo-	~ 68 kDa (a)	67 970.70 +/- 2.55 Da (Minor)<br>68 101.43 +/- 1.15 Da (Major0	Without first N-ter Met

(a) No sequence available

Supplementary Discussion:

The sample contains one unique protein Kf (*exo*⁻) population with the expected molecular weight. The Kf (*exo*⁻) fragment exists in two forms: without (minor form) and with (major form) the first *N*-terminal methionine residue. The sample is highly pure and no contaminant, including phosphatases, could be detected. This analysis confirms the certificate of analysis obtained from the company New England Biolabs.
